# Phase 1 evaluation of APN401, an autologous CBL-B-silenced PBMC therapy, in advanced solid tumors

**DOI:** 10.1016/j.omton.2026.201357

**Published:** 2026-10-05

**Authors:** Alexander Dohnal, Nina Worel, Thomas Bogenrieder, Thorsten Fuereder, Martin Burian, Dominik Wolf, Gerald Prager, Richard Greil, Anja Peterbauer-Scherb, Beate Schrenk, Stefan Bunka, Ernesto Abila, Felix Batrina, Maria Urban, Julia Buchberger, Alice Schwarzboeck, Bernhard Peball, Sandra Seidl, Hannes Muehleisen, Stefan Stranner, Mario Kuttke, Angela Halfmann, Sarah Bischof, Andreas Birbach, Romana Gugenberger

**Affiliations:** 1 InvIOs GmbH, Campus Vienna Biocenter 5, 1030 Vienna, Austria; 2 Medical University of Vienna, Department of Transfusion Medicine and Cell Therapy, Waehringer Guertel 18-20, 1090 Vienna, Austria; 3 Medical University of Vienna, Department of Medicine I, Division of Oncology, Waehringer Guertel 18-20, 1090 Vienna, Austria; 4 Ordensklinikum Linz GmbH, Barmherzige Schwestern, Seilerstaette 4, 4020 Linz, Austria; 5 Medical University Innsbruck, Department of Internal Medicine V, Hematology and Oncology, Anichstrasse 35, 6020 Innsbruck, Austria; 6 Salzburg Cancer Research Institute, Center for Clinical Cancer and Immunology Trials, Paracelsus Medical University Salzburg, Muellner Hauptstrasse 48, 5020 Salzburg, Austria; 7 Austrian Red Cross, Blood Transfusion Service for Upper Austria, Krankenhaustrasse 7, 4010 Linz, Austria

**Keywords:** APN401, CBL-B, adoptive cellular therapy, cancer immunotherapy, small interfering RNA (siRNA), peripheral blood mononuclear cells, PBMCs, intracellular immune checkpoint, T cell activation, solid tumors, point-of-care manufacturing

## Abstract

Intracellular immune checkpoints regulate activation thresholds in cytotoxic immune cells. Casitas B-lineage lymphoma proto-oncogene B (CBL-B) is an E3 ubiquitin ligase that negatively regulates antigen receptor signaling and T cell activation. APN401 is an autologous peripheral blood mononuclear cell (PBMC) therapy incorporating transient small interfering RNA (siRNA)-mediated CBL-B silencing. Two phase 1 studies evaluated escalating doses of APN401 in patients with advanced treatment-refractory solid tumors. APN401 was manufactured from leukapheresis-derived PBMCs using the EPiC point-of-care platform. Seventeen patients received APN401 across three dose levels. APN401 demonstrated a favorable safety profile, with no dose-limiting toxicities, cytokine release syndrome, immune effector cell-associated neurotoxicity syndrome, or grade ≥3 treatment-related adverse events. Manufacturing was reproducible across sites. Although no objective responses were observed, disease stabilization occurred in 5 of 10 Response Evaluation Criteria in Solid Tumors (RECIST)-evaluable patients (50.0%), corresponding to 29.4% of all 17 treated patients. Exploratory analyses identified treatment-associated systemic immune changes, including enrichment of circulating CD8^+^ T cells, induction of cytotoxic mediators, and expansion of dominant T cell receptor β (TCRβ) clonotypes. These changes were most pronounced in patients with stable disease but remain exploratory and hypothesis-generating. These findings support further evaluation of APN401 in larger, controlled studies.

## Graphical abstract

**Figure:**
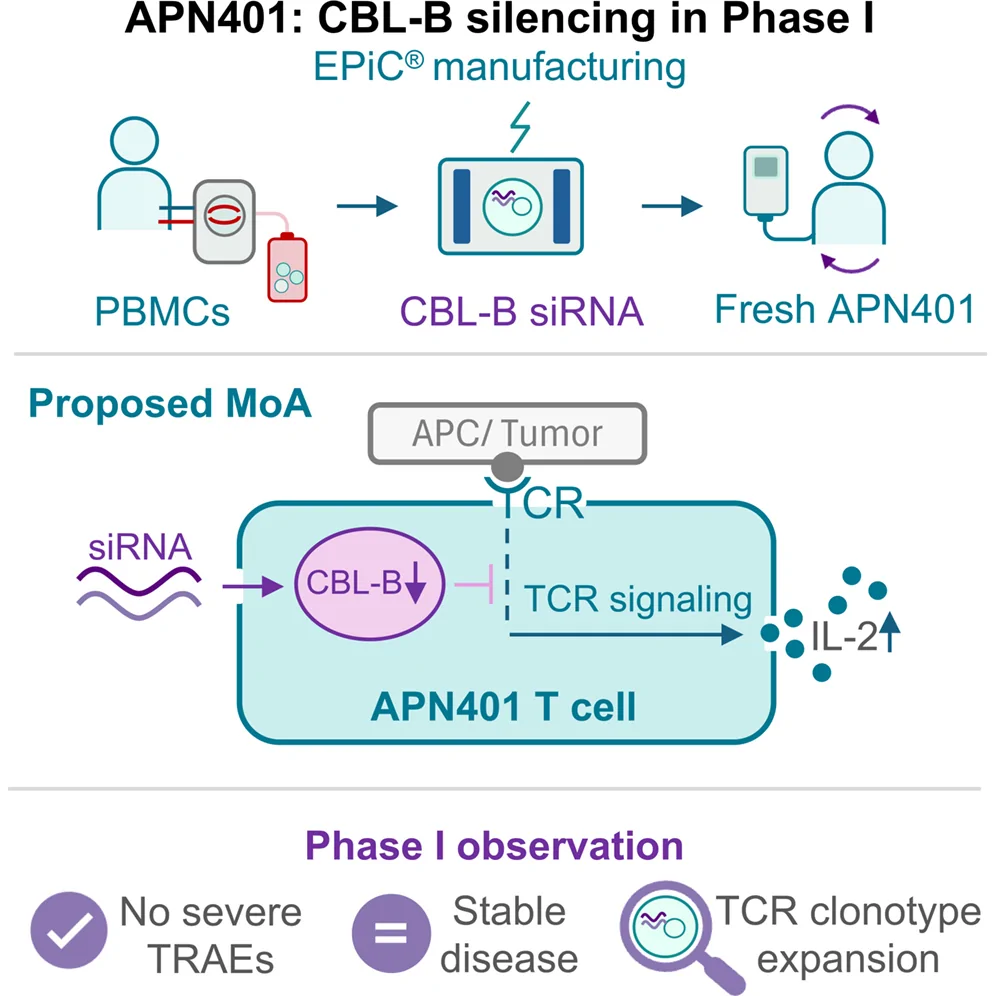

## Teaser

APN401 combines transient CBL-B silencing in autologous PBMCs with rapid point-of-care manufacturing. In two phase 1 studies, repeated APN401 administration was feasible and well tolerated. Exploratory analyses identified systemic immune modulation and T cell clonal expansion, supporting further investigation of transient intracellular checkpoint modulation in solid tumors.

## Introduction

Immunotherapy has transformed cancer treatment, with immune checkpoint inhibitors producing durable clinical benefit across multiple tumor types. However, many patients with advanced malignancies fail to achieve sustained responses due to immunosuppressive tumor microenvironments, antigen heterogeneity, and progressive dysfunction of tumor-reactive T cells.^1^^,^^2^^,^^3^^,^^4^^,^^5^ Additional immunotherapeutic approaches, including cellular therapies, have demonstrated activity in selected settings but remain limited in solid tumors by impaired tumor infiltration, antigen escape, and manufacturing complexity.^6^^,^^7^^,^^8^ These challenges highlight the need for complementary strategies that enhance intrinsic immune cell function.

Intracellular signaling pathways regulating activation thresholds represent an emerging target space for immune modulation. Casitas B-lineage lymphoma proto-oncogene B (CBL-B), an E3 ubiquitin ligase, is a key negative regulator of antigen receptor signaling that increases the threshold for T cell activation.^9^^,^^10^ Preclinical studies have shown that CBL-B inhibition enhances T cell activation and promotes antitumor immunity.^11^^,^^12^ Furthermore, CBL-B regulates both adaptive and innate immune-cell function, including T and natural killer (NK) cells, providing a rationale for therapeutic CBL-B inhibition across multiple immune-cell compartments.^9^^,^^13^

To translate this concept clinically, APN401 was developed as an autologous peripheral blood mononuclear cell (PBMC)-based therapy incorporating transient small interfering RNA (siRNA)-mediated silencing of CBL-B. Early phase 1 clinical evaluation of APN401 in patients with advanced solid tumors demonstrated the feasibility of *ex vivo* siRNA-mediated CBL-B silencing in autologous PBMCs and tolerability of APN401 administration.^14^^,^^15^ These findings provided the basis for evaluating repeated APN401 administration and a rapid point-of-care manufacturing approach in subsequent clinical development. Along these lines, APN401 was manufactured using the enhancement platform for immune cells (EPiC), enabling same-day production of modified, autologous PBMC products from leukapheresis-derived cells.

Here, we report results from two phase 1 studies evaluating APN401 in a broad population of patients with advanced treatment-refractory solid tumors, reflecting the safety and feasibility objectives of early-phase development rather than patient selection based on established tumor-specific sensitivity to CBL-B inhibition in immune cells. Hence, the studies focused on safety, manufacturing feasibility, and exploratory immune activity associated with transient intracellular checkpoint modulation.

## Results

### APN401 manufacturing and proposed mechanism of action

The APN401 manufacturing workflow and proposed mechanism of action are summarized in Figure 1. APN401 is generated from autologous leukapheresis-derived PBMCs using the EPiC point-of-care manufacturing platform (Figure 1A). Following PBMC purification, unfractionated PBMCs are electroporated with CBL-B-targeting siRNA, formulated as APN401, subjected to pre-infusion release testing, and reinfused into the patient within a vein-to-vein time of <24 h. Post-infusion functional characterization is performed on retained drug-product (DP) samples following T cell receptor (TCR) stimulation. Following reinfusion, antigen encounter and TCR activation are expected to induce CBL-B expression as part of physiological negative-feedback signaling. The transfected CBL-B-specific siRNA limits this activation-induced CBL-B expression, thereby transiently reducing negative regulation of TCR signaling and enhancing T cell activation, cytokine production, proliferation, and cytotoxic function (Figure 1B).

**Figure 1:**
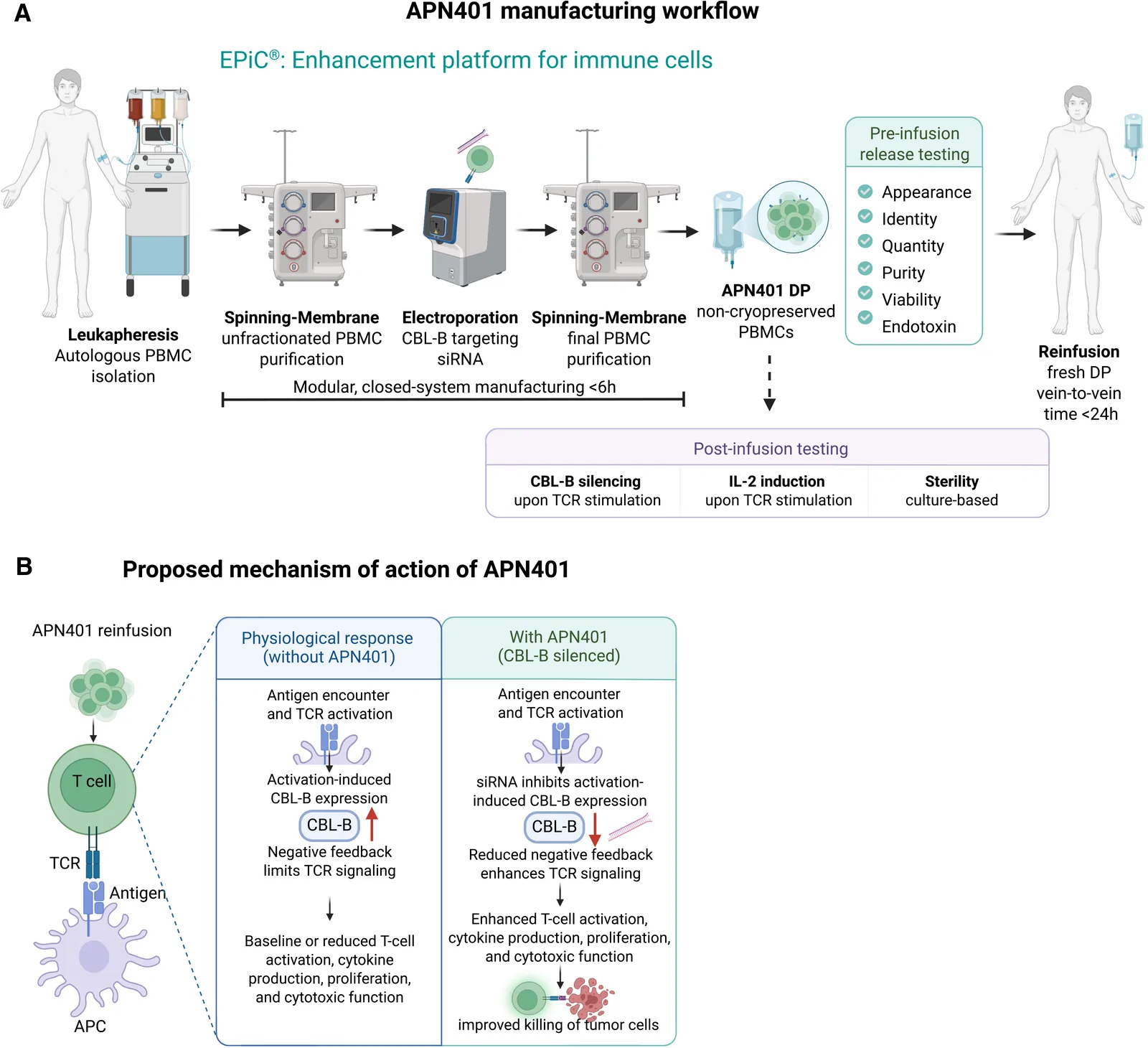
APN401 manufacturing workflow and proposed mechanism of action (A) APN401 is manufactured from autologous peripheral blood mononuclear cells (PBMCs) collected by leukapheresis. Following closed-system PBMC purification, unfractionated PBMCs are electroporated with CBL-B-targeting siRNA, followed by removal of cell debris and residual reagents and final formulation of APN401. The drug-product (DP) undergoes pre-infusion release testing for appearance, identity, quantity (cell count), purity, viability, and endotoxin before reinfusion, with a vein-to-vein time of <24 h. Sterility and functional characterization of retained DP samples is performed post-infusion and includes assessment of *CBLB* mRNA knockdown and CBL-B protein downregulation and IL-2 induction following T cell receptor (TCR) stimulation. (B) Proposed mechanism of action of APN401. Following reinfusion, antigen encounter, and TCR activation are expected to induce CBL-B expression as part of physiological negative-feedback signaling. CBL-B-specific siRNA introduced during APN401 manufacturing limits activation-induced CBL-B expression, thereby transiently reducing negative regulation of TCR signaling and enhancing T cell activation, cytokine production, proliferation, and cytotoxic function, which may improve recognition and killing of tumor cells. APC, antigen-presenting cell; CBL-B, Casitas B-lineage lymphoma proto-oncogene B; siRNA, small interfering RNA; TCR, T cell receptor. Created in BioRender. Kuttke, M. (2026) https://BioRender.com/qzshtzm.

### Patients and treatments

Nineteen patients were screened across APN401-103 and APN401-104, and 17 patients received at least one APN401 infusion and comprised the pooled safety population (Table 1; Tables S1 and S2). Patients represented a heterogeneous population of advanced treatment-refractory solid tumors and had received at least two prior lines of systemic therapy. In APN401-104, patients received a median of 4 prior lines of therapy (range, 3–8), and 6 patients had prior exposure to immune checkpoint inhibitors. Patients received escalating doses ranging from 5 × 10^6^ to 4.5 × 10^7^ PBMCs/kg and completed a median of 3 treatment cycles (range, 1–6). Dose escalation in APN401-103 evaluated dose levels 1 (5 × 10^6^ PBMCs/kg) and 2 (1.5 × 10^7^ PBMCs/kg), whereas APN401-104 evaluated dose levels 2 and 3 followed by expansion at dose level 3 (4.5 × 10^7^ PBMCs/kg), which was subsequently selected as the recommended phase 2 dose (RP2D). Treatment discontinuation primarily reflected disease progression or clinical deterioration in this heavily pretreated population. Additional reasons included withdrawal in the patient’s best interest (*n* = 4), patient withdrawal (*n* = 1) of consent, and out-of-specification (OOS, *n* = 2) manufacturing batches identified before infusion (Table S2). Overall, 51 of 56 manufactured batches met pre-infusion release specifications (Figure S1). APN401 was manufactured and administered as a fresh, non-cryopreserved cell product without an intervening cryopreservation and thawing step. Of the five OOS batches, two did not meet the predefined identity specification and three did not meet the predefined quantity specification. These events did not result in modification of the APN401 manufacturing process but informed subsequent narrowing of the acceptance specification for the leukapheresis starting material. Post-electroporation DP viability was 76.4% ± 7.2% of viable PBMCs, and functional characterization demonstrated reduction of *CBLB* mRNA by 56.0% ± 14.2% and reduction of CBL-B protein expression by 91.4% ± 10.7% relative to matched controls.

**Table 1:** Patient demographics and disease characteristics

Parameter	APN401-103	APN401-104
**Number of patients receiving APN401 (*n*)**	6	11
**Age (years)**		
Minimum-maximum	59–71	50–79
Mean (SD)	64.7 (4.23)	62.5 (8.21)
Median	65.5	60.0
**Race/ethnicity (*n*)**		
White	6	11
Asian	0	0
Other	0	0
**Sex (*n*)**		
Male	4	6
Female	2	5
**ECOG performance status at baseline (*n*)**		
0–2 (eligibility criterion)	6	11
**Previous lines of systemic therapy**		
≥2 prior lines (eligibility criterion)	6 (100.0)	11 (100.0)
**Tumor type (*n*)**		
Colorectal carcinoma	1	1
Head and neck squamous cell carcinoma (HNSCC)	2	3
Pancreatic carcinoma	2	0
Appendiceal carcinoma	1	0
Non-small-cell lung cancer (NSCLC)	0	1
Small-cell lung cancer (SCLC)	0	1
Cholangiocarcinoma	0	1
Uveal melanoma	0	3
Melanoma	0	1

### Safety and tolerability

All treated patients (*n* = 17) were included in the safety population. APN401 demonstrated a favorable safety profile, with no dose-limiting toxicities (DLTs), cytokine release syndrome, immune effector cell-associated neurotoxicity syndrome (ICANS), grade ≥3 treatment-related adverse events (TRAEs), treatment-related serious adverse events (AEs), or treatment-related deaths. AEs reported in ≥3 patients are summarized in Table 2. Most events were grade 1–2 and attributable to study procedures or underlying disease. TRAEs primarily included transient infusion-associated symptoms such as fatigue, pyrexia, nausea, dizziness, and headache. No patients discontinued treatment because of treatment-related toxicity.

**Table 2:** Adverse events reported in ≥3 patients

Adverse event	Patients, *n* (%)
Implant site hematoma	11 (64.7%)
Fatigue	10 (58.8%)
Abdominal pain	6 (35.3%)
General physical health deterioration	5 (29.4%)
Ascites	4 (23.5%)
Constipation	4 (23.5%)
Nausea	4 (23.5%)
Dyspepsia	4 (23.5%)
Implant site hemorrhage	3 (17.6%)
Implant site pain	3 (17.6%)
Pyrexia	3 (17.6%)
Arthralgia	3 (17.6%)
Dyspnea	3 (17.6%)
Myalgia	3 (17.6%)
Anemia	3 (17.6%)

Adverse events are shown irrespective of relationship to APN401.

### Determination of the RP2D

Three dose levels were evaluated: dose level 1 (*n* = 3), dose level 2 (*n* = 7), and dose level 3 (*n* = 7) (Table S2). No DLTs occurred at any dose level. Following Bayesian optimal interval (BOIN) analysis and isotonic regression adjustment, the estimated probability of toxicity remained below the prespecified target toxicity rate of 25% across all dose levels. Based on the favorable tolerability profile, dose level 3 (4.5 × 10^7^ PBMCs/kg) was selected as the RP2D. A maximum tolerated dose was not reached within the dose range evaluated. RP2D selection was based on the phase 1 dose-escalation and safety assessment and was not based on clinical response rates.

### Clinical activity

Best overall response (BOR) according to RECIST v.1.1^16^ was evaluable in 10 of 17 treated patients (Tables S3 and S2). Seven patients were not evaluable for BOR because of early treatment termination, including two following OOS manufacturing batches, four best-interest withdrawals, and one patient withdrawal. No objective responses were observed. Stable disease (SD) occurred in 5 of 10 evaluable patients, corresponding to a disease control rate (DCR) of 50.0%, or 29.4% when calculated across all 17 treated patients, whereas the remaining RECIST-evaluable patients experienced progressive disease (PD). Disease stabilization was most frequently observed at dose level 2. Given the small number of RECIST-evaluable patients within each dose level, these findings do not establish a dose-response relationship for clinical activity. Tumor response kinetics and treatment exposure are summarized in Figures 2 and 3. Most treatment discontinuations and deaths were attributable to disease progression.

**Figure 2:**
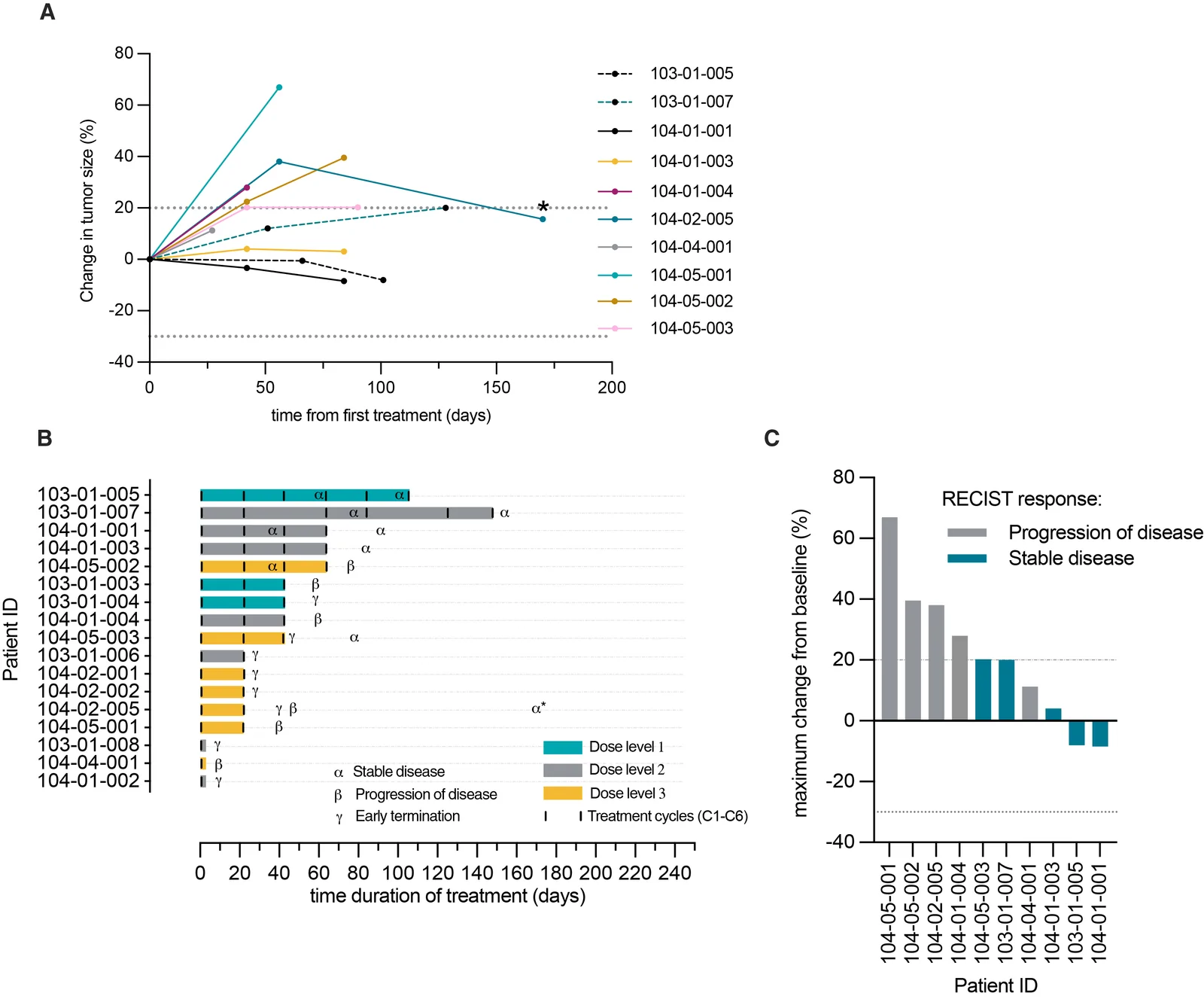
Tumor response and treatment exposure following APN401 therapy (A) Longitudinal percentage change in target-lesion size from baseline in patients with available target-lesion measurements (*n* = 10). Dashed lines indicate RECIST v.1.1 thresholds for partial response (−30%) and progressive disease (+20%). (B) Swimmer plot showing treatment exposure and clinical course in the safety population (*n* = 17). Each bar represents an individual patient and indicates the duration of APN401 treatment. Symbols indicate stable disease (α), progressive disease (β), or early termination (γ). ∗Reduction in target lesion measurements observed during follow-up. (C) Maximum percentage change in target-lesion size from baseline in patients with available target-lesion measurements (*n* = 10). Each bar represents an individual patient; colors indicate stable disease or progressive disease according to RECIST v.1.1. BOR, best overall response; RECIST, Response Evaluation Criteria in Solid Tumors.

**Figure 3:**
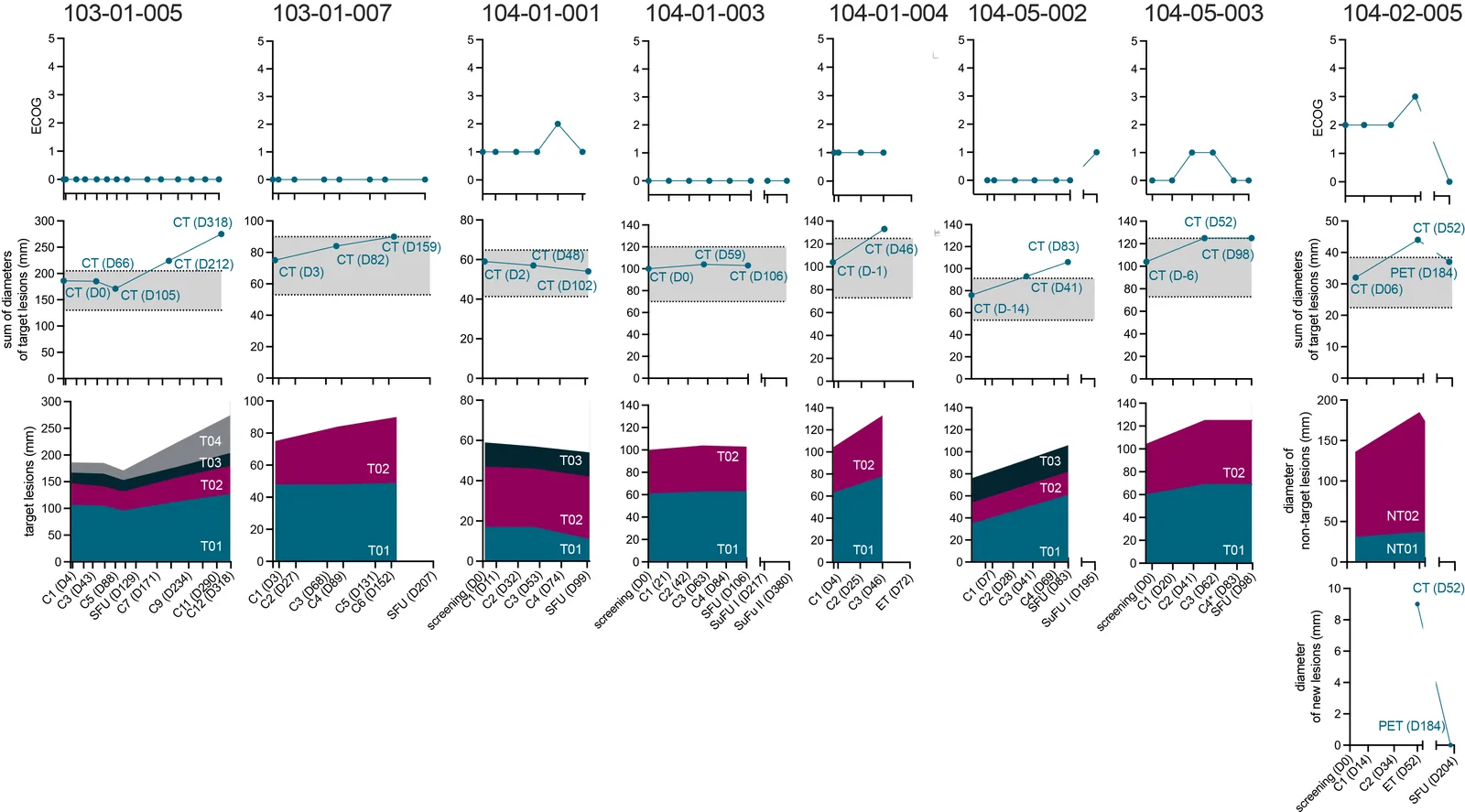
Clinical and radiographic assessments in selected APN401-treated patients Clinical status and radiographic tumor assessments were evaluated longitudinally in selected patients with sufficient follow-up to assess longitudinal tumor kinetics. ECOG performance status and target lesion measurements were collected at screening, during treatment cycles, and during follow-up assessments. Stacked plots show the sum of target lesion (T) measurements over time. Non-target lesions (NT) and new lesions are indicated where applicable. Tumor assessments were performed according to RECIST v.1.1 criteria. Patient 104-05-003 did not receive cycle 4 because no DP was manufactured.

One patient with appendiceal carcinoma (103-01-005) demonstrated prolonged disease stabilization and remained alive 4 years after treatment initiation. The patient received subsequent chemotherapy after radiographic progression during extended APN401 treatment; therefore, the prolonged clinical course cannot be attributed to APN401 alone. Another patient with recurrent/metastatic head and neck squamous cell carcinoma (HNSCC) (104-02-005) discontinued treatment after two cycles following clinical progression and withdrawal in the patient’s best interest; however, follow-up imaging approximately 170 days after the first infusion demonstrated a reduction in target lesions and resolution of previously detected lesions. No intervening anticancer therapy was administered during this interval. Because iRECIST criteria were not prospectively implemented, this delayed radiographic finding cannot be classified as pseudoprogression or definitively attributed to APN401.

### Immune biomarker analyses

Exploratory immune biomarker analyses were performed to characterize systemic immune changes associated with APN401 treatment and to evaluate immune features observed in patients with disease stabilization. Given the limited sample size, these analyses were descriptive and hypothesis-generating and were not intended to establish statistical significance. Longitudinal analyses were performed using paired samples collected at baseline (pre-cycle 1), post-cycle 1, and post-cycle 2. Baseline samples were obtained before APN401 manufacturing and therefore reflected the untreated immune state.

### Treatment-associated immune activation

Comparison of baseline and post-cycle 1 samples demonstrated early modulation of multiple immune parameters (Figure 4A), including changes in circulating CD8^+^ T cell populations, memory-associated T cell subsets, activation-associated markers, and soluble immune mediators. Post-cycle 2 analyses demonstrated continued immune remodeling across lymphoid and myeloid compartments (Figures 4B and 4C), including changes in CD8^+^ T cell abundance, CD8^+^/CD4^+^ T cell ratios, B cell populations, monocyte subsets, and myeloid-derived suppressor cell (MDSC)-associated parameters. Longitudinal analyses also demonstrated delayed upregulation of inhibitory receptors including PD-1, TIM-3, and LAG-3 during treatment (Figure S2). Patients with SD demonstrated longitudinal changes in markers associated with cytotoxic T cell activation and systemic immune remodeling, including increased CD137^+^ and TIM-3^+^ CD8^+^ T cells, elevated plasma interferon-γ (IFN-γ), and expansion of circulating B cell populations. In contrast, PD was associated with higher monocyte levels and increased plasma IL-10. These immune changes coincided with expansion of dominant TCRβ clonotypes in selected patients (Figure S3). The antigen specificity of these expanded clonotypes was not determined.

**Figure 4:**
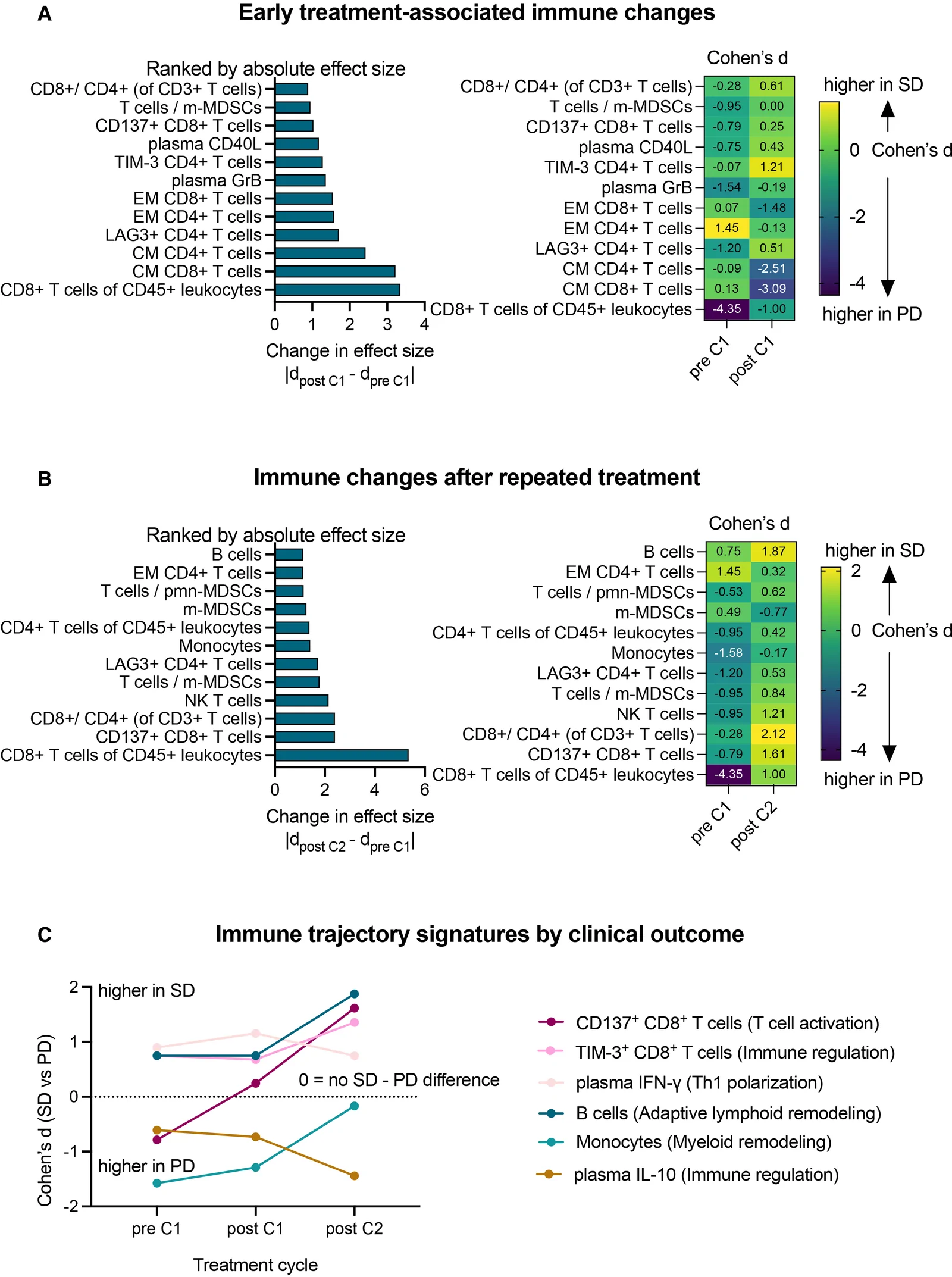
Longitudinal immune marker dynamics during APN401 treatment Peripheral immune markers were analyzed longitudinally in patients with disease stabilization (SD-associated group, *n* = 4) and progressive disease (PD, *n* ≥ 3) in study APN401-104. (A and B) Bars rank immune markers by the absolute change in Cohen’s *d* from baseline (pre-C1) to post-cycle 1 (A) or post-cycle 2 (B); adjacent heatmaps show Cohen’s *d* at each time point. (C) Temporal trajectories of Cohen’s *d* for representative immune markers. Positive values indicate higher levels in SD and negative values higher levels in PD. Immune subsets include lymphoid populations and monocytic (m)- and polymorphonuclear (pmn)-myeloid-derived suppressor cells (MDSCs).

### Baseline immune features associated with clinical outcome

Baseline immune profiles were analyzed to explore pretreatment immune features associated with clinical outcome (Figures 5 and S4). Patients with PD demonstrated higher baseline levels of several T cell subsets including CD8^+^ T cells and plasma granzyme B (GrB), whereas patients with SD demonstrated higher baseline plasma IFN-γ and increased frequencies of selected MDSC subsets. Given the small sample size and exploratory nature of these analyses, these baseline differences should be considered descriptive and hypothesis-generating.

**Figure 5:**
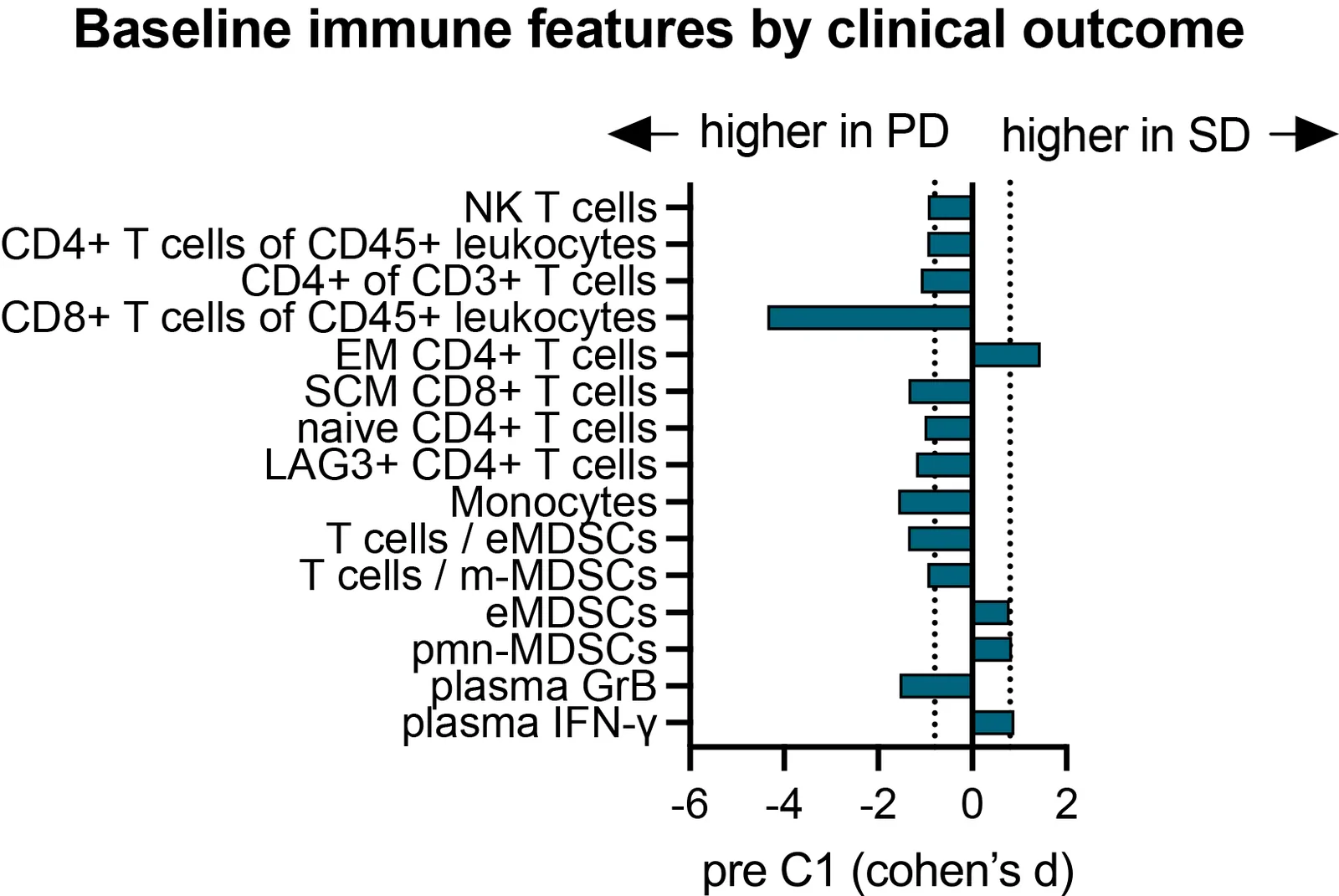
Baseline immune features associated with clinical outcome Baseline immune markers measured prior to APN401 treatment (pre-C1) were compared between patients with disease stabilization (SD, *n* = 4) and progressive disease (PD, *n* = 4) in study APN401-104. Bars represent standardized effect sizes (Cohen’s *d*, SD vs. PD). Positive values indicate markers enriched in SD, whereas negative values indicate markers enriched in PD. The dashed line indicates strong effect size (|Cohen’s *d*| ≥ 0.8). Immune subsets include lymphoid populations and monocytic (m)- and polymorphonuclear (pmn)- myeloid-derived suppressor cell (MDSC) subsets.

### Representative patients with immune correlates

Two patients with extended longitudinal immune monitoring are shown to illustrate heterogeneous immune response kinetics observed during APN401 treatment.

Patient 103-01-005 had metastatic appendiceal carcinoma and had received 11 prior lines of therapy before enrollment. Patient 103-01-005 received six cycles of APN401 at dose level 1 (5 × 10^6^ PBMCs/kg) and achieved SD during the protocol-defined treatment period, with largely stable target lesion measurements on serial imaging (Figure 3). Following study completion, the patient received six additional APN401 cycles through a named-patient use program (total *n* = 12). Although radiographic progression occurred during extended treatment, disease stabilization was reestablished after APN401 discontinuation and subsequent chemotherapy. The patient remained alive and clinically stable at both 2- and 4-year survival follow-up (SuFu) visits. Given the intervening chemotherapy, the contribution of APN401 to the subsequent prolonged clinical course cannot be determined.

Functional characterization of APN401 DPs demonstrated sustained activity across manufacturing batches (Figure S5). Following anti-CD3/CD28 stimulation, DPs consistently showed increased secretion of IL-2, CD137, IFN-γ, and CXCL13 compared with matched non-CBL-B-silenced controls across cycles C1-C5, indicating reproducible enhancement of T cell activation after CBL-B silencing.

Longitudinal immune monitoring revealed dynamic modulation of circulating immune mediators during treatment, including early increases in plasma GrB and transient induction of IFN-γ and soluble CD40L (sCD40L), consistent with enhanced cytotoxic activity and T cell-antigen-presenting cell interactions (Figures 6A and S5). Peripheral immune phenotyping demonstrated early enrichment of circulating CD8^+^ T cells and an increased CD8^+^/CD4^+^ T cell ratio (Figure S3). TCRβ sequencing revealed expansion of multiple dominant clonotypes during treatment, several of which persisted at the 2-year SuFu visit (Figure 6B). Single-cell RNA sequencing localized these clonotypes to CD8^+^ effector and central memory T cell subsets (Figure S6), demonstrating persistence of these clonotypes within CD8^+^ T cell populations, although their tumor specificity was not established.

**Figure 6:**
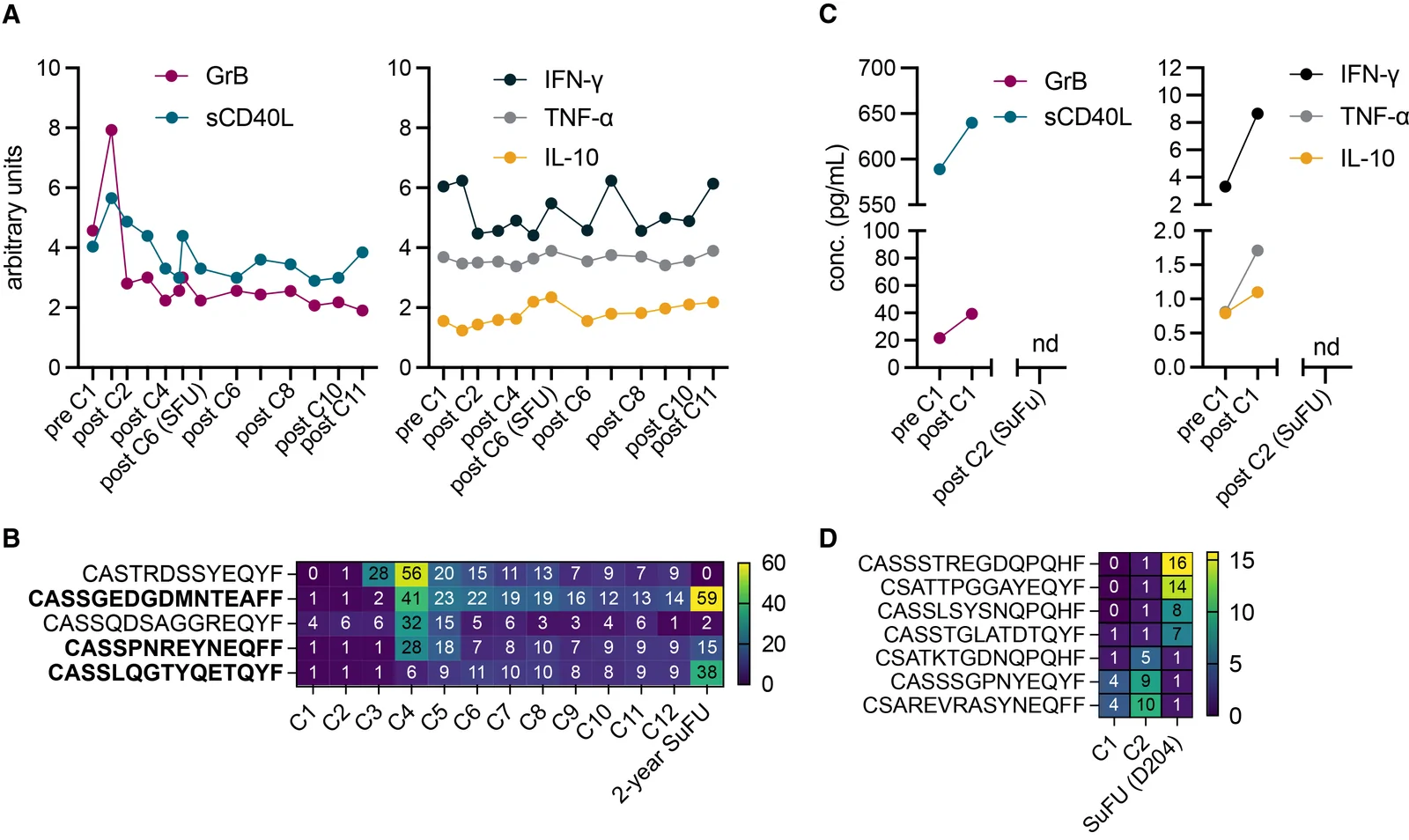
Longitudinal immune monitoring in representative APN401-treated patients (A and B) Patient 103-01-005 (appendiceal carcinoma), who achieved disease stabilization during protocol-defined APN401 treatment and underwent longitudinal immune monitoring through survival follow-up. (A) Longitudinal plasma levels of interferon-γ (IFN- γ), tumor necrosis factor-α (TNF- α), granzyme B (GrB), soluble CD40L (sCD40L), and IL-10, expressed in arbitrary units, at baseline (pre-C1) and subsequent treatment and follow-up time points. (B) TCRβ repertoire profiles across treatment cycles C1-C12 and at 2-year survival follow-up (SuFu). (C and D) Patient 104-02-005 (recurrent/metastatic head and neck squamous cell carcinoma), who discontinued APN401 following clinical progression and withdrawal in the patient’s best interest and subsequently showed a reduction in target-lesion measurements during post-treatment follow-up. (C) Plasma concentrations of IFN- γ, TNF-α, GrB, sCD40L, and IL-10 at baseline (pre-C1) and after cycle 1 (post-C1), expressed in pg/mL; concentrations at SuFu were not determined. (D) TCRβ repertoire profiles from cycles 1 and 2 and at SuFu (day 204 after screening). For TCRβ analyses, each row represents an individual clonotype, and color intensity indicates relative clonotype abundance across time points. Clonotypes with bold labels were phenotyped by single-cell RNA sequencing (Figure S6). nd, not determined.

Patient 104-02-005 had recurrent/metastatic HNSCC and had received three prior lines of systemic therapy. This patient received two APN401 cycles at the (RP2D; 4.5 × 10^7^ PBMCs/kg) before treatment discontinuation following clinical progression and withdrawal in the patient’s best interest. No intervening anticancer therapy was administered between APN401 discontinuation and follow-up imaging performed 170 days after the first infusion, which demonstrated a reduction in target lesions (Figures 2 and 3). Because iRECIST was not prospectively implemented, this delayed radiographic finding cannot be classified as pseudoprogression or definitively attributed to APN401.

Plasma analysis revealed marked induction of immune activation markers after a single treatment cycle, including GrB, sCD40L, and IFN-γ (Figure 6C). Peripheral immune phenotyping demonstrated enrichment of circulating CD4^+^ and CD8^+^ T cells (Figure S3). In parallel, TCRβ sequencing identified expansion of dominant clonotypes following treatment, with several clones increasing from baseline and becoming highly enriched at survival follow-up (SuFu; day 204 after screening) (Figures 6D and S3). These clonal expansions coincided with CD8^+^ T cell enrichment and preceded the delayed reduction in target lesions observed on positron emission tomography (PET)-computed tomography (CT) imaging. The tumor specificity of the expanded clonotypes was not established, and their temporal association with the subsequent radiographic finding does not demonstrate a causal relationship.

Together, these cases illustrate heterogeneous longitudinal immune changes observed following APN401 treatment. In patient 103-01-005, immune activation and persistent T cell clonotypes were observed during longitudinal follow-up, whereas in patient 104-02-005, early immune changes and clonal T cell expansion preceded the later reduction in target lesion measurements. Given the intervening chemotherapy in patient 103-01-005 and the limitations of interpreting post-treatment imaging in patient 104-02-005, these observations are descriptive and do not establish a causal relationship between APN401-associated immune changes and clinical outcome.

Collectively, APN401 demonstrated feasible decentralized manufacturing, favorable tolerability, and exploratory evidence of systemic immune modulation in patients with advanced solid tumors. Although objective responses were not observed, disease stabilization and treatment-associated immune changes were identified in a subset of patients.

## Discussion

Immunotherapy has transformed cancer treatment, yet many patients with advanced solid tumors fail to achieve durable benefit because of immunosuppressive tumor microenvironments, antigen heterogeneity, and progressive T cell dysfunction.^1^^,^^2^^,^^3^^,^^4^^,^^5^ These limitations highlight the need for complementary strategies that enhance immune responses beyond established surface checkpoint pathways. Targeting intracellular immune checkpoints represents a complementary approach by modulating activation thresholds downstream of antigen receptor signaling.^9^ CBL-B is a key negative regulator of T cell activation that physiologically increases the threshold for antigen-receptor signaling and contributes to the maintenance of peripheral immune tolerance.^9^^,^^10^ Accordingly, permanent loss of CBL-B function enhances immune-cell activation and antitumor immunity but has also been associated with increased susceptibility to autoimmunity in experimental models.^10^^,^^11^^,^^12^ More recent unbiased genome-wide CRISPR screening in primary human T cells independently identified CBL-B among intracellular regulators whose disruption conferred resistance across multiple immunosuppressive conditions relevant to the tumor microenvironment, further supporting CBL-B as a therapeutic intracellular checkpoint.^17^ Transient modulation may therefore provide an opportunity to enhance immune-cell responsiveness while avoiding permanent genetic disruption of this physiologically important regulatory pathway. APN401 was developed on this principle as an autologous PBMC-based therapy incorporating transient siRNA-mediated CBL-B silencing. Manufacturing using the EPiC platform was consistently feasible, enabling rapid (∼6 h), decentralized, point-of-care production. APN401 was administered as a fresh, non-cryopreserved PBMC product, avoiding an intervening cryopreservation and thawing step within this rapid manufacturing workflow. In contrast to conventional autologous cell therapies limited by manufacturing complexity, capacity constraints, and prolonged vein-to-vein times,^18^^,^^19^ this approach supports repeated dosing and may preserve cell fitness by avoiding extended *ex vivo* culture.^6^^,^^7^^,^^8^ The reproducibility across sites supports its scalability as a practical delivery strategy. Allogeneic cell therapies aim to overcome logistical constraints through off-the-shelf availability and standardized manufacturing but face challenges including host-mediated rejection, limited persistence, and the need for genetic modification to control alloreactivity.^20^ In heterogeneous solid tumors, autologous approaches may additionally preserve endogenous tumor-reactive immune repertoires. In this context, APN401 combines preservation of the endogenous immune repertoire with rapid point-of-care manufacturing.

APN401 demonstrated a favorable safety profile, with no DLTs, cytokine release syndrome, ICANS, or grade ≥3 TRAEs. Compared with tumor-infiltrating lymphocyte (TIL) and engineered T cell therapies, which are frequently associated with substantial toxicities,^6^^,^^7^^,^^8^ these findings suggest that transient intracellular checkpoint silencing may provide a comparatively manageable safety profile, although cross-trial comparisons remain limited. The use of unfractionated PBMCs preserves immune-cell populations beyond T cells, and CBL-B is known to negatively regulate activation signaling in several leukocyte subsets, including NK cells, B cells, and myeloid cells.^9^^,^^13^ However, the functional contribution of these non-T cell populations to APN401 activity was not specifically assessed in the present studies. Along these lines, APN401 does not specifically enrich for tumor-reactive lymphocytes, and the trafficking and tissue distribution of the infused cell populations were not assessed; therefore, attenuation of tumor-directed activity by non-tumor-reactive cell populations cannot be excluded.

Although no objective responses were observed, SD occurred in 5 of 10 RECIST-evaluable patients (50.0%), corresponding to 29.4% of all 17 treated patients. Notably, three of five patients with SD had received prior immune checkpoint inhibitor therapy. The absence of objective responses cannot be attributed to a specific mechanism in these studies; in particular, the contribution of transient siRNA-mediated CBL-B silencing and persistence of modified cells could not be assessed because their kinetics were not directly quantified. Patients with SD nevertheless demonstrated systemic immune modulation, including CD8^+^ T cell enrichment, induction of cytotoxic mediators, and expansion of dominant TCRβ clonotypes,^21^ despite the absence of objective tumor regression. Separately, patient 104-02-005, who had discontinued APN401 following clinical progression and withdrawal in the patient’s best interest, showed a reduction in target lesions on follow-up imaging approximately 170 days after the first infusion without intervening anticancer therapy. The significance of this delayed radiographic finding remains uncertain because iRECIST was not prospectively implemented.^22^

Exploratory treatment-associated immune changes were consistent with systemic immune modulation. Patients with SD showed increases in markers associated with cytotoxic T cell activation, including CD137^+^ and TIM-3^+^ CD8^+^ T cells, plasma IFN-γ, and GrB, together with increased circulating B cell populations. GrB is a major effector protease of cytotoxic lymphocytes,^23^ while CD137 can identify activated antigen-reactive T cells^24^^,^^25^ and CD40L contributes to T cell-antigen-presenting cell interactions.^26^ Increased circulating B cells may also be relevant given the association of tumor-infiltrating B cells and tertiary lymphoid structures with favorable immunotherapy outcomes,^27^ although the significance of circulating B cell changes remains uncertain. In patient 103-01-005, CXCL13 was robustly induced following anti-CD3/CD28 stimulation of longitudinal APN401 DPs; CXCL13 mediates CXCR5-dependent B cell recruitment and organization, including within tertiary lymphoid structures.^28^ However, CXCL13 was assessed in DPs from this patient only, and a relationship with the treatment-associated increase in circulating B cells cannot be established.

The longitudinal increase in inhibitory receptors, including PD-1, TIM-3, and LAG-3, may reflect physiological feedback following immune activation, although an evolving dysfunctional or exhausted T cell state cannot be excluded without functional validation.^29^^,^^30^^,^^31^ Baseline immune profiles also differed between outcome groups: patients with PD had higher circulating CD8^+^ T cell frequencies and plasma GrB, whereas patients with SD had higher IFN-γ and selected MDSC subsets. These findings should not be interpreted as predictive biomarkers. Higher CD8^+^ T cell and GrB levels in PD may reflect a pre-existing but functionally insufficient or dysregulated immune response,^29^^,^^30^^,^^31^ whereas IFN-γ has pleiotropic roles in antitumor immunity and adaptive immune regulation.^32^ The higher baseline MDSC levels in patients with SD appear counterintuitive given their generally immunosuppressive role; however, MDSCs are heterogeneous, and circulating abundance may not reflect their functional state within the tumor microenvironment.^33^ The biological significance of these baseline differences therefore remains uncertain. Increased IL-10 observed during treatment in patients with PD may reflect counter-regulatory immune signaling; however, given the context-dependent functions of IL-10, its cellular source and biological significance cannot be inferred from circulating levels alone.^34^

Longitudinal observations in individual patients further illustrated heterogeneous immune response kinetics. In patient 103-01-005, dominant TCRβ clonotypes persisted during and beyond APN401 treatment,^21^ including within CD8^+^ effector- and central-memory populations, consistent with preclinical evidence that CBL-B deficiency can support sustained antitumor T cell responses and long-lasting antitumor memory.^12^ In patient 104-02-005, early systemic immune changes and clonal T cell expansion preceded the later reduction in target lesion measurements. The antigen specificity of the expanded clonotypes was not established in either patient; therefore, although these observations are consistent with systemic immune modulation following transient CBL-B silencing, they do not establish a causal relationship between the observed immune changes and clinical outcome.

Several limitations of these studies are inherent to their phase 1 design, including the small sample size, heterogeneous tumor histologies, limited number of response-evaluable patients, and exploratory biomarker analyses not powered for statistical inference. The absence of objective responses limits conclusions regarding clinical activity, and the delayed radiographic observation in patient 104-02-005 remains difficult to interpret because iRECIST was not prospectively implemented. The duration of CBL-B silencing in APN401 cells after reinfusion and the persistence of the modified cells could not be directly determined because this would require specific tracking of infused cells to distinguish them from endogenous circulating PBMCs. Such labeling would require additional manipulation of the DP with the potential to affect cellular function and potency and was therefore not implemented. Furthermore, the studies did not include an *in vivo* control cohort receiving similarly processed autologous PBMCs without CBL-B-targeting siRNA; therefore, contributions of leukapheresis, electroporation, and PBMC reinfusion to the observed systemic immune changes cannot be excluded.

Future studies should determine whether the observed immune modulation translates into meaningful clinical benefit and whether baseline immune features can inform patient selection. The exploratory findings provide a rationale for evaluating repeated dosing and combination approaches with immune checkpoint inhibitors, while their potential to improve clinical activity remains to be established.^1^^,^^2^^,^^3^ Pharmacologic targeting of CBL-B is also being explored, further supporting the translational relevance of this pathway.^35^^,^^36^

In summary, these phase 1 studies demonstrate the feasibility of decentralized, same day point-of-care manufacturing and repeated administration of autologous CBL-B-silenced PBMCs, together with a favorable safety profile. Exploratory immune changes observed during treatment provide biological rationale for further investigation of transient intracellular checkpoint modulation in larger, controlled studies.

## Materials and methods

### Study design and participants

The open-label multicenter phase 1 studies APN401-103 (European Union Clinical Trials Register: EU-CT 2020–002680-55) and APN401-104 (EU-CT 2023–504281-34/ClinicalTrials.gov: NCT06172894) evaluated the safety, feasibility, and RP2D of APN401 in patients with advanced treatment-refractory solid tumors. Both studies were approved by institutional ethics committees and conducted in accordance with the Declaration of Helsinki. All patients provided written informed consent. Eligible patients were ≥18 years of age with histologically confirmed advanced solid tumors, Eastern Cooperative Oncology Group (ECOG) performance status 0–2, measurable disease according to RECIST v.1.1^16^ and ≥2 prior lines of systemic therapy. Dose escalation followed a conventional 3 + 3 design in APN401-103 and a BOIN design in APN401-104.

### Treatment and dose escalation

Patients received escalating doses of APN401 ranging from dose level 1 (5 × 10^6^ PBMCs/kg) to dose level 2 (1.5 × 10^7^ PBMCs/kg) and dose level 3 (4.5 × 10^7^ PBMCs/kg). Treatment was administered for up to six cycles at 3-week intervals. Each treatment cycle consisted of leukapheresis on day 1 followed by APN401 infusion on day 2. Subsequent treatment cycles were administered in the absence of disease progression, unacceptable toxicity, and active infection, based on investigator assessment, provided that the manufactured product met release specifications and no criteria for early study termination were met. No lymphodepleting or immunosuppressive conditioning regimen was administered before APN401 infusion.

### Endpoints and assessments

The primary endpoints were safety, tolerability, manufacturing feasibility, and determination of the RP2D. Secondary endpoints included preliminary clinical activity assessed by BOR according to RECIST v.1.1^16^ and characterization of systemic immune modulation associated with APN401 treatment. Safety was monitored through clinical assessments and AE reporting, with DLTs evaluated during the 21-day observation period following the first infusion. Clinical activity was evaluated by BOR, duration of disease stabilization, and survival follow-up where available. BOR was defined as the best response observed from treatment initiation until disease progression, initiation of new anticancer therapy, or study discontinuation. Patients were considered evaluable for BOR if they had at least one post-baseline tumor assessment or discontinued because of disease progression. Exploratory pharmacodynamic analyses were performed on longitudinal plasma and PBMC samples collected before treatment cycles and included flow cytometry-based phenotyping, soluble mediator analysis, TCR repertoire sequencing, and single-cell transcriptomics. Detailed assay methodologies are provided in the supplemental methods.

### Manufacturing and quality control

APN401 DPs were manufactured from autologous PBMCs collected by leukapheresis and transiently silenced for CBL-B using the EPiC manufacturing platform. PBMCs were electroporated with good manufacturing practice (GMP)-grade CBL-B-targeting siRNA incorporating 2′-O-methyl modifications designed to enhance DP potency; the resulting DP was formulated for infusion. A detailed description of the EPiC platform architecture is shown in Figure 1A and provided in the supplemental methods. APN401 was administered as a fresh, non-cryopreserved autologous PBMC product without an intervening cryopreservation and thawing step. APN401 batch release included pre-administration quality control testing (appearance, identity, viability, purity, quantity, and endotoxin). Additional testing for sterility, activity, and potency was performed post-administration using retained samples. Activity and potency testing using anti-CD3/CD28 stimulation assays is described in the supplemental methods. DP quality control results are shown in Figure S1.

### Statistical analysis

As a phase 1 dose-escalation study, sample size was not based on formal statistical power calculations. Safety and clinical outcomes were summarized descriptively. Exploratory biomarker analyses were conducted to evaluate treatment-associated immune changes and differences between clinical outcome groups. These analyses were descriptive and hypothesis-generating and were not intended to establish statistical significance. Effect size-based approaches were used to characterize observed differences, with longitudinal analyses performed across predefined time points. Detailed statistical methodologies are provided in the supplemental methods.

## Data and code availability

The data generated in this study are available from the corresponding authors upon reasonable request. Individual participant data are not publicly available due to patient privacy and confidentiality considerations.

## Acknowledgments

The authors acknowledge financial support from the 10.13039/501100004955Austrian Research Promotion Agency (FFG) under grant agreement no. FO999905116. The authors thank the patients and their families for participation in this study, as well as the clinical and technical staff at all participating sites for their contributions to study conduct and sample processing.

## Author contributions

Conceptualization, A.D., N.W., T.B., T.F., S.B., and R. Gugenberger; methodology, A.D., N.W., T.B., A.B., R. Gugenberger, E.A., F.B., M.U., J.B., A.S., B.P., S. Seidl, H.M., S. Stranner, M.K., A.H., and S.B.; investigation, N.W., T.F., M.B., D.W., G.P., R. Greil, A.P.-S., B.S., S.B., E.A., F.B., M.U., J.B., A.S., B.P., S. Seidl, H.M., S. Stranner, M.K., A.H., and S.B.; data curation, A.D., E.A., F.B., M.U., J.B., A.S., B.P., S. Seidl, H.M., M.K., A.H., S.B., and R. Gugenberger; formal analysis, A.D., R. Gugenberger, A.B., A.H., S.B., and T.B.; resources, N.W., T.F., M.B., D.W., G.P., and R. Greil; funding acquisition, S.B.; project administration, A.D., S.B., and A.B.; supervision, A.D., N.W., T.F. and T.B.; visualization, A.D. and T.B.; writing – original draft, A.D.; writing – review and editing, A.D., N.W., T.B., T.F., M.B., D.W., G.P., R. Greil, S.B., S. Stranner, M.K., S.B., A.B., and R. Gugenberger All authors reviewed and approved the final manuscript and agree to be accountable for all aspects of the work.

## Declaration of interests

Authors affiliated with invIOs GmbH are or have been employees of the company and may hold equity or stock options. invIOs GmbH is developing APN401 and the EPiC platform described in this study. These authors report patents and/or patent applications related to the technology described herein. T.F. reports honoraria or advisory roles from MSD, Merck, BMS, BI, Roche, Sanofi, Amgen, Takeda, Johnson & Johnson, Beigene, Pierre Fabre, Pfizer, Pharma Mar, Astra Zeneca, Abbvie, Regeneron and invIOs GmbH. N.W. reports honoraria from BMS Celgene, Kite Gilead, Miltenyi Biotec, Terumo BCT, Therakos, Vertex, and research support from Sanofi Genzyme.

## Declaration of generative AI and AI-assisted technologies in the manuscript preparation process

During the preparation of this work, the authors used ChatGPT (OpenAI) to assist with drafting, language refinement, editing, and improving the clarity of the manuscript. The authors reviewed, edited, and validated all AI-generated content and take full responsibility for the final content of the publication. No artificial intelligence tool was used to generate, analyze, interpret, or verify study data, nor to make scientific conclusions.

## Supplemental information

Document S1. Figures S1–S6 and Tables S1–S3

Document S2. Article plus supplemental information
