# Supplementary material for: Phase 1 evaluation of APN401, an autologous CBL-B-silenced PBMC therapy, in advanced solid tumors
Source: Mol Ther Oncol. 2026 Oct 5;34(4):201357. doi: 10.1016/j.omton.2026.201357 (PMC13717735; doi:10.1016/j.omton.2026.201357)
Supplement: Supplementary file 1 [file mmc1.pdf]

OMTON, Volume *34*

Supplemental information

Phase 1 evaluation of APN401, an autologous CBL-B-silenced PBMC therapy, in advanced solid tumors

Alexander Dohnal, Nina Worel, Thomas Bogenrieder, Thorsten Fuereder, Martin Burian, Dominik Wolf, Gerald Prager, Richard Greil, Anja Peterbauer-Scherb, Beate Schrenk, Stefan Bunka, Ernesto Abila, Felix Batrina, Maria Urban, Julia Buchberger, Alice Schwarzboeck, Bernhard Peball, Sandra Seidl, Hannes Muehleisen, Stefan Stranner, Mario Kuttke, Angela Halfmann, Sarah Bischof, Andreas Birbach, and Romana Gugenberger

Supplemental Figures

APN401 Batch-Manufacturing

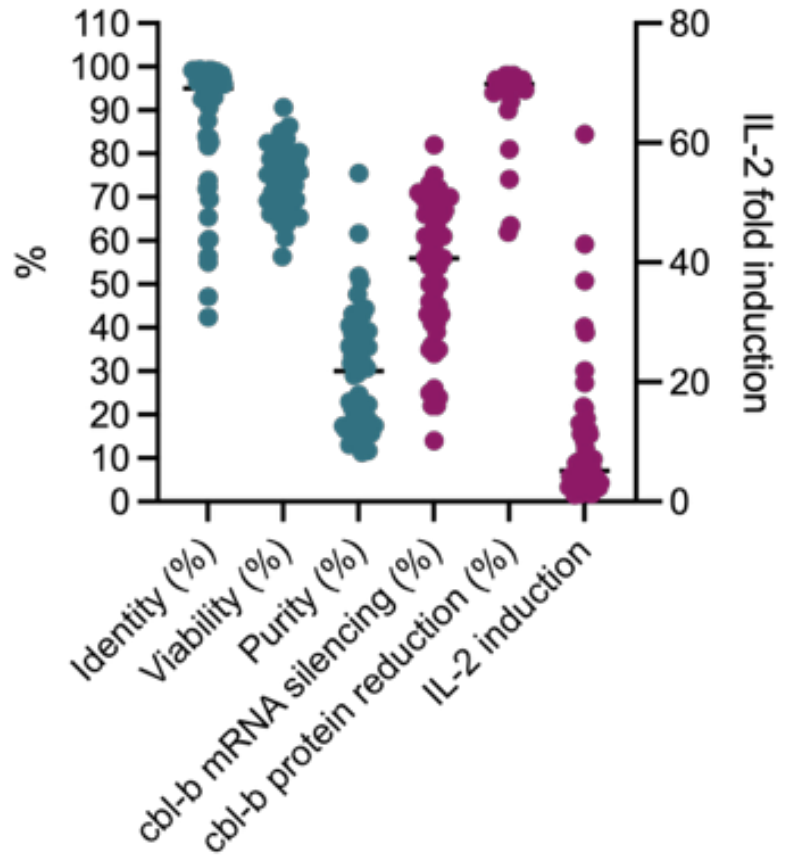

Figure S1. Release Testing and Functional Characterization of Manufactured APN401 Drug Product Batches. *Fifty-six APN401 DP batches underwent pre-infusion release testing for appearance, identity, viability, purity, quantity, and endotoxin. Post-infusion functional characterization following anti-CD3/CD28 stimulation assessed CBL-B silencing at the mRNA and protein levels relative to matched no-pulse controls (NPCs), as well as IL-2 secretion as a potency readout. Median values of tested batches are shown.*

Biomarker Assessment

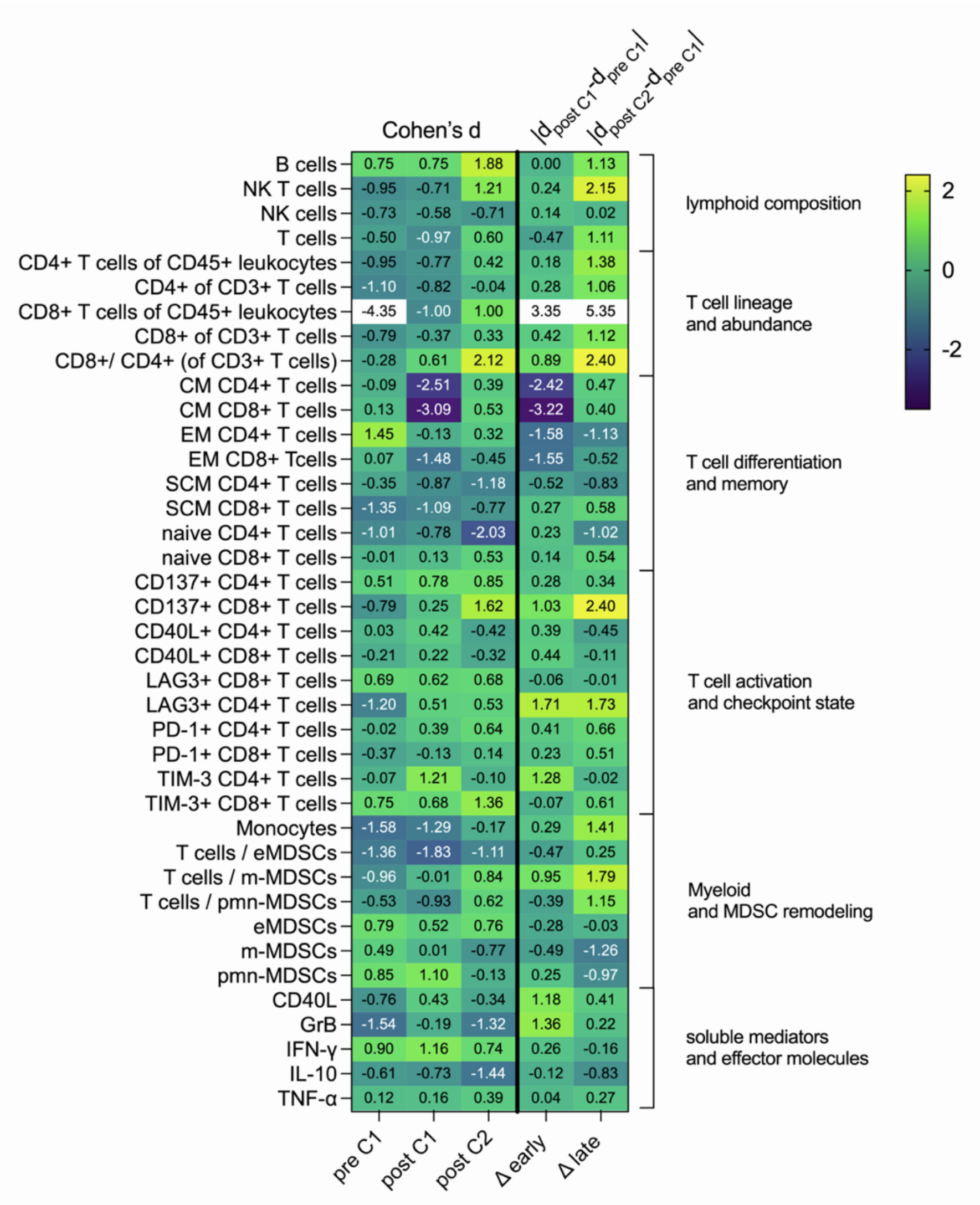

Figure S2. Systemic Immune Remodeling across APN401 Treatment Cycles. *Heatmap summarizing peripheral blood immune parameters at baseline (pre C1), post-cycle 1 (post C1), and post-cycle 2 (post C2), together with longitudinal changes from baseline. Standardized effect sizes (Cohen's d) compare patients with stable disease (SD) and progressive disease (PD). For longitudinal analyses, Cohen's d was calculated by comparing within-patient changes from baseline between the SD and PD groups. Positive effect sizes indicate higher values or greater increases in SD, whereas negative effect sizes indicate higher values or greater increases in PD. Immune parameters were assessed by multiparameter flow cytometry and plasma immunoassays. Absolute Cohen's d values of 0.2, 0.5, and 0.8 were interpreted descriptively as small, moderate, and large effects, respectively. Analyses included evaluable patients with paired samples at the indicated time points. Immune subsets included lymphoid populations and early-stage (eMDSC), monocytic (M-MDSC), and polymorphonuclear (PMN-MDSC) myeloid-derived suppressor cell subsets.*

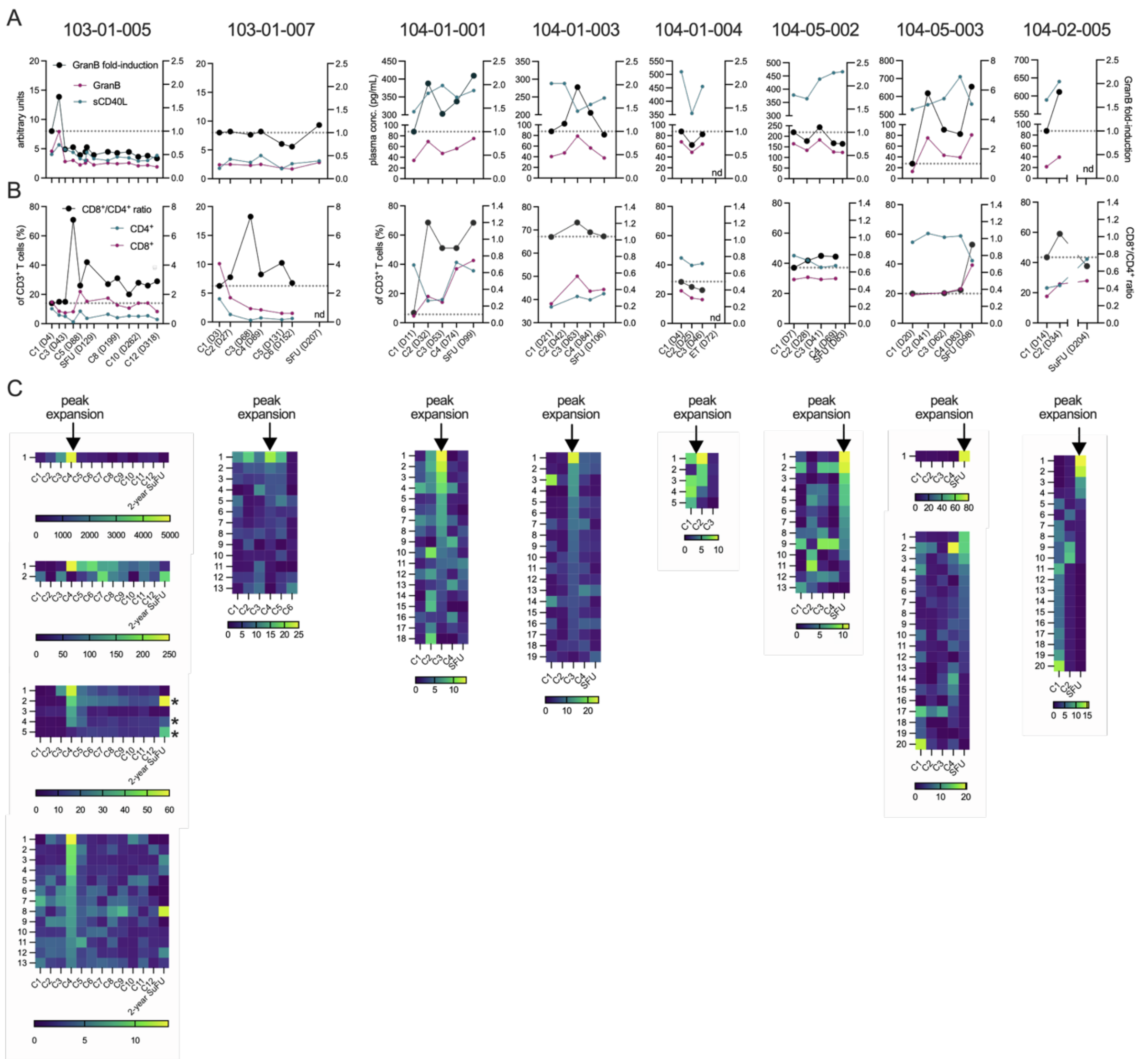

Figure S3. Longitudinal Immune Monitoring in Selected APN401-Treated Patients. *Exploratory immune monitoring parameters were evaluated longitudinally in patients with sufficient follow-up to assess immune kinetics. Peripheral blood samples were collected prior to each APN401 treatment cycle, at safety follow-up (SFU), and at survival follow-up (SuFu), where applicable. (A) Plasma concentrations of soluble immune mediators measured longitudinally using Olink® multiplex proteomics assays and/or electrochemiluminescence-based immunoassays. (B) Longitudinal frequencies of circulating $CD4^+$ and $CD8^+$ T cells and $CD8^+/CD4^+$ T-cell ratios determined by multiparameter flow cytometry. (C) Longitudinal dynamics of TCRβ clonotypes assessed by TCRβ repertoire sequencing during treatment and follow-up. Only clonotypes showing ≥5-fold enrichment during treatment are displayed and are shown relative to the lowest observed read count. Clonotypes also identified by single-cell RNA sequencing are indicated (*), where applicable.*

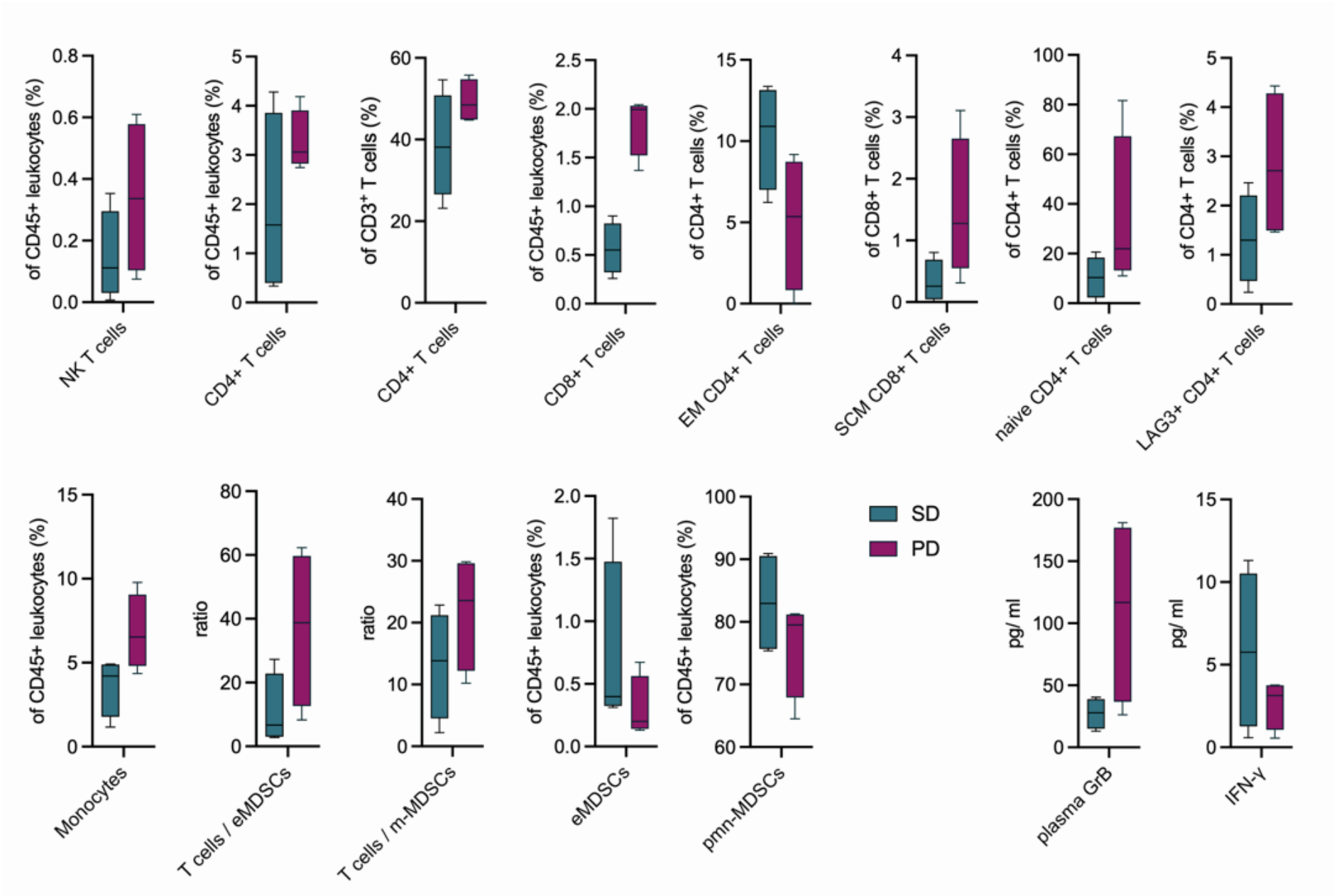

Figure S4. Baseline Peripheral Immune Features Associated with Stable Disease or Progressive Disease. *Baseline peripheral immune parameters were compared descriptively between patients with disease stabilization (SD, teal) and progressive disease (PD, purple). Box plots show immune cell subset frequencies measured by multiparameter flow cytometry, T-cell/MDSC ratios, and plasma concentrations of soluble mediators including GrB and IFN-γ. Boxes represent median and interquartile range, with whiskers indicating minimum and maximum values (SD, n = 4; PD, n = 4).*

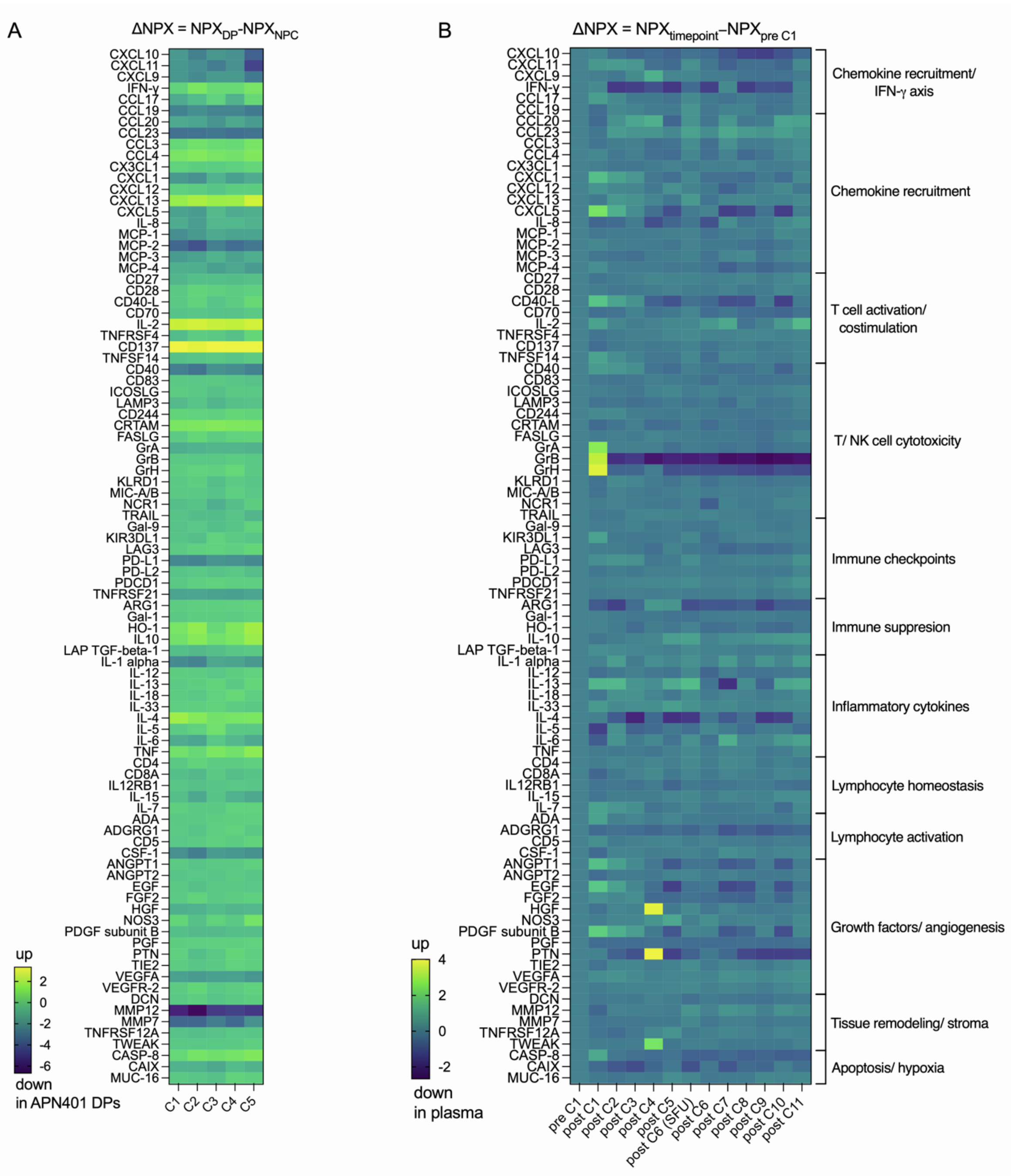

Figure S5. Longitudinal Proteomic Profiling of APN401 Drug Products and Plasma from Patient 103-01-005. *Heatmaps show longitudinal changes in immune-related proteins measured using the Olink® Immuno-Oncology panel. (A) Protein levels in culture supernatants from anti-CD3/CD28–stimulated APN401 DPs across manufacturing cycles (C1–C5) relative to matched no-pulse controls (NPCs). (B) Longitudinal plasma protein levels following APN401 administration (post C1 to post C11) relative to baseline (pre C1). The color scale indicates relative upregulation or downregulation of protein abundance. Normalized Protein eXpression (NPX) values represent log2-scaled normalized protein expression.*

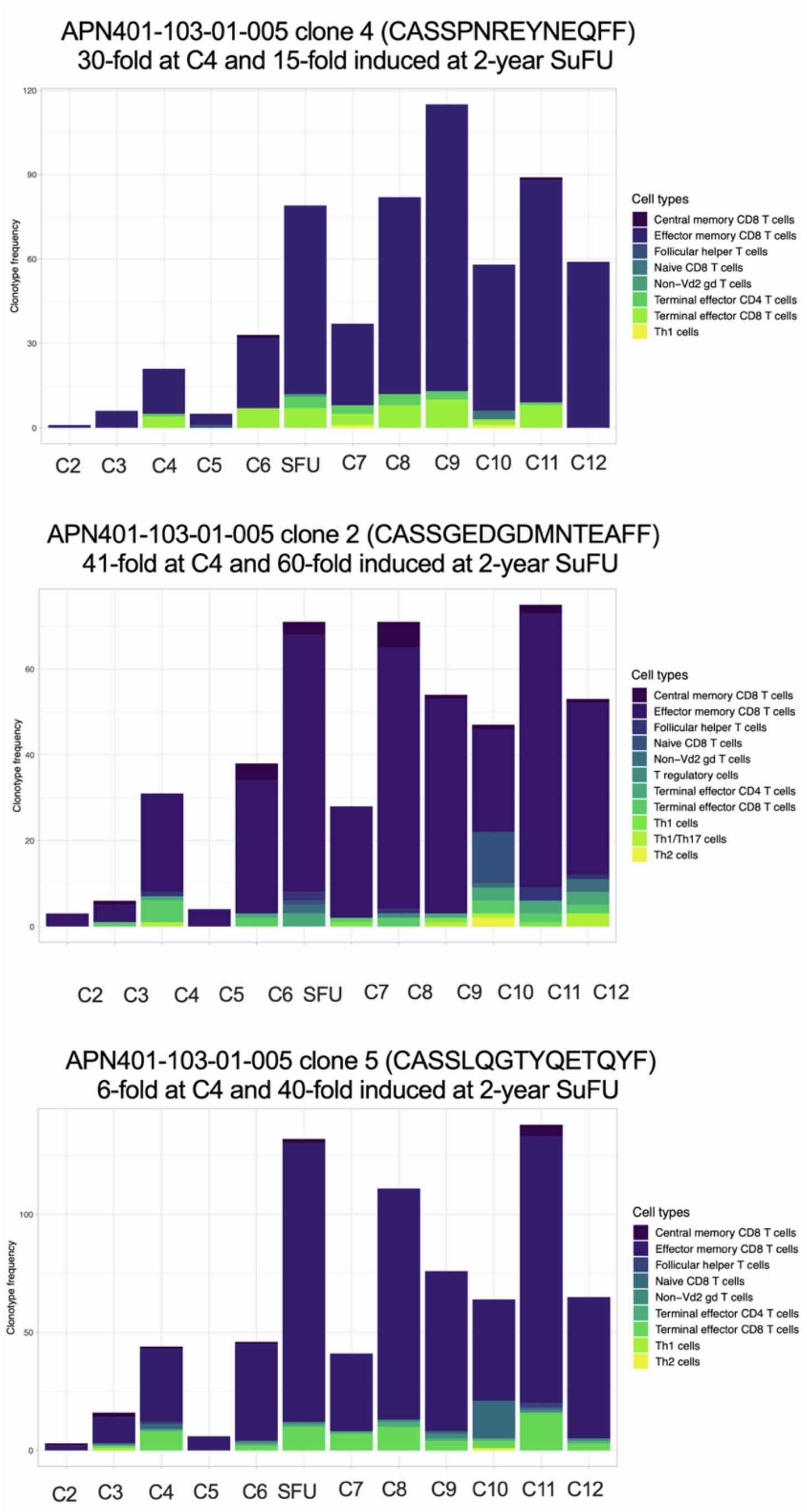

Figure S6. Phenotypic Characterization of Expanded TCRβ Clonotypes in Patient 103-01-005. *Phenotypic profiles of selected expanded TCRβ clonotypes in patient 103-01-005 are shown. Clonotypes detected by single-cell RNA sequencing of PBMCs were matched to corresponding clonotypes identified by TCRβ repertoire sequencing of APN401 DPs. Stacked bar plots show the phenotypic composition of matched clonotypes across treatment cycles and follow-up assessments, based on cell-type annotation derived from single-cell transcriptomic analysis. Fold induction in clonotype abundance relative to baseline is indicated for selected time points. All data are presented descriptively.*

Supplemental Tables

Table S1. Representativeness of Study Participants

Category	*Considerations*
Cancer type(s)/stage(s)/condition	*Patients with advanced, treatment-refractory solid tumors across multiple histologies. Not intended to represent any specific tumor epidemiology.*
Sex	*10 male, 7 female; not representative of tumor-specific distributions.*
Age	*Broad adult age range; representativeness limited by the small sample size and heterogeneous tumor types.*
Race/ethnicity	*All patients were White; racial and ethnic diversity was limited.*
Geography	*Conducted at four Austrian centers; limited global generalizability.*
Other considerations	*Selection based on eligibility for leukapheresis and early-phase trial participation.*
Overall representativeness	*Reflects a treatment-refractory Phase I population; limited generalizability due to sample size, tumor heterogeneity, and geographic restriction.*

Table S2. Patient Characteristics, Treatment and Clinical Response

Cohort	*Subject ID*	*Tumor type*	*Prior lines*	*Dose level*	*Safety population*	*Treatment cycles*	*BOR-evaluable*	*RECIST BOR*	*Early termination/ reason*
APN401 -103	*103-01-001*	*HNSCC*	*≥2*	*1*	*No*	*0*	*No*	*na*	*/*
APN401 -103	*103-01-002*	*HNSCC*	*≥2*	*1*	*No*	*0*	*No*	*na*	*/*
APN401 -103	*103-01-003*	*HNSCC*	*≥3*	*1*	*Yes*	*3*	*Yes*	*PD*	*/*
APN401 -103	*103-01-004*	*Colorectal carcinoma*	*≥2*	*1*	*Yes*	*3*	*No*	*ET*	*OOS*
APN401 -103	*103-01-005*	*Appendiceal carcinoma*	*11*	*1*	*Yes*	*6+6**	*Yes*	*SD*	*/*
APN401 -103	*103-01-006*	*Pancreatic carcinoma*	*≥2*	*2*	*Yes*	*2*	*No*	*ET*	*Best-interest withdrawal*
APN401 -103	*103-01-007*	*HNSCC*	*≥2*	*2*	*Yes*	*6*	*Yes*	*SD*	*/*
APN401 -103	*103-01-008*	*Pancreatic carcinoma*	*≥2*	*2*	*Yes*	*1*	*No*	*ET*	*Best-interest withdrawal*
APN401 -104	*104-01-001*	*SCLC*	*4§*	*2*	*Yes*	*4*	*Yes*	*SD*	*/*
APN401 -104	*104-01-002*	*NSCLC*	*8§*	*2*	*Yes*	*1*	*No*	*ET*	*OOS*
APN401 -104	*104-01-003*	*Colorectal carcinoma*	*6§*	*2*	*Yes*	*4*	*Yes*	*SD*	*/*
APN401 -104	*104-01-004*	*Cholangio carcinoma*	*5*	*2*	*Yes*	*3*	*Yes*	*PD*	*/*
APN401 -104	*104-02-001*	*HNSCC*	*3*	*3*	*Yes*	*2*	*No*	*ET*	*Patient withdrawal*
APN401 -104	*104-02-002*	*HNSCC*	*3*	*3*	*Yes*	*2*	*No*	*ET*	*Best-interest withdrawal*
APN401 -104	*104-02-005*	*HNSCC*	*3*	*3*	*Yes*	*2*	*No*	*ET&*	*Best-interest withdrawal*
APN401 -104	*104-04-001*	*Melanoma*	*4§*	*3*	*Yes*	*1*	*Yes*	*PD*	*/*
APN401 -104	*104-05-001*	*Uveal melanoma*	*4*	*3*	*Yes*	*2*	*Yes*	*PD*	*/*
APN401 -104	*104-05-002*	*Uveal melanoma*	*3§*	*3*	*Yes*	*4*	*Yes*	*PD*	*/*
APN401 -104	*104-05-003*	*Uveal melanoma*	*5§*	*3*	*Yes*	*3*	*Yes*	*SD*	*/*

BOR, best overall response; ET, early termination; HNSCC, head and neck squamous cell carcinoma; NSCLC, non-small cell lung cancer; OOS, out of specification; PD, progressive disease; RECIST, Response Evaluation Criteria in Solid Tumors; SCLC, small cell lung cancer; SD, stable disease.

** Six APN401 cycles were administered during protocol-defined study treatment, followed by six additional cycles through a named-patient use program (12 cycles total).*

§ Received ≥1 prior line of immune checkpoint inhibitor therapy.

& Delayed reduction in target lesion measurements observed following early treatment discontinuation.

Table S3: Best Overall Response in the RECIST-Evaluable Population

BOR n (%)	*DL1 (n = 2)*	*DL2 (n = 4)*	*DL3 (RP2D) (n = 4)*	*Overall (n = 10)*
SD	*1 (50.0%)*	*3 (75.0%)*	*1 (25.0%)*	***5 (50.0%)***
PD	*1 (50.0%)*	*1 (25.0%)*	*3 (75.0%)*	***5 (50.0%)***
ORR	*0 (0.0%)*	*0 (0.0%)*	*0 (0.0%)*	*0 (0.0%)*
95% CI	*(0.0%, 84.2%)*	*(0.0%, 60.2%)*	*(0.0%, 60.2%)*	*(0.0%, 30.8%)*
DCR	*1 (50.0%)*	*3 (75.0%)*	*1 (25.0%)*	*5 (50.0%)*
95% CI	*(1.3%, 98.7%)*	*(19.4%, 99.4%)*	*(0.6%, 80.6%)*	*(18.7%, 81.3%)*

BOR, best overall response; CI, confidence interval; CR, complete response; DCR, disease control rate; DL, dose level; ORR, objective response rate; PD, progressive disease; PR, partial response; RECIST, Response Evaluation Criteria in Solid Tumors; RP2D, recommended Phase II dose; SD, stable disease. DCR was defined as CR + PR + SD; ORR was defined as CR + PR. The RECIST-evaluable population included 10 of 17 treated patients; seven patients were not evaluable for BOR because of early treatment termination.

Supplemental Material and Methods

APN401 Manufacturing, Drug Product Supply, and Release Testing

APN401 drug products (DPs) were manufactured at two GMP-compliant manufacturing sites supplying four clinical centers in Austria. To enable administration within the defined product shelf life, all clinical sites were located within approximately 5 hours' driving distance of the respective manufacturing facility. Leukapheresis products were collected at the clinical sites and, after meeting predefined starting-material specifications, were processed the following day using the EPiC® (Enhancement Platform for Immune Cells) manufacturing platform. Manufacturing was completed within approximately 6 hours, including pre-infusion release testing.

Manufacturing Process

Leukapheresis-derived peripheral blood mononuclear cells (PBMCs) were rebuffered into electroporation buffer (MaxCyte, Inc.) using spinning membrane filtration (LOVO®, Fresenius Kabi).

Good Manufacturing Practice (GMP)-grade Casitas B-lineage lymphoma proto-oncogene B (CBL-B)-targeting siRNA incorporating 2'-O-methyl modifications designed to enhance DP potency was introduced into PBMCs by electroporation using the MaxCyte GTx® system in accordance with the manufacturer's instructions.

Following electroporation, cells were rested for 30–60 minutes at room temperature and subsequently rebuffered for final formulation using spinning membrane filtration.

This modular configuration supports reproducible manufacturing across clinical sites and enables rapid production compatible with repeated dosing schedules.

APN401 siRNA characterization

The CBL-B-targeting siRNA used for APN401 is a chemically modified 21-mer RNA duplex incorporating 2'-O-methyl modifications. During product development, modified and unmodified siRNA variants were evaluated, with the modified siRNA demonstrating increased DP potency. The selected modified siRNA

was additionally evaluated for potential off-target effects. Transcriptome-level analyses did not identify evidence of sequence-specific off-target silencing under the tested conditions. Ribosome-occupancy analysis showed no significant differences indicative of broader effects on translational efficiency, and eCLIP-based analysis detected no binding of either the sense or antisense strand to off-target transcripts. Together, these analyses showed no detectable off-target binding or sequence-specific off-target silencing under the experimental conditions tested.

Pre-Infusion Release Testing

Pre-administration quality control testing was performed at the manufacturing site immediately before shipment and infusion. Release criteria included appearance, identity, quantity, viability, purity, and endotoxin.

- ***Appearance:*** *Visual inspection of the DP within the container closure system was performed against a standardized background and color reference, with aggregate presence and size recorded.*
- ***Identity and Quantity:*** *Determined by complete blood count (CBC) with differential. PBMCs were defined as the sum of lymphocytes and monocytes. Identity was defined as the percentage of PBMCs among total leukocytes (lymphocytes, monocytes, granulocytes), and quantity was calculated based on CBC results, target PBMC dose per kilogram, and patient body weight.*
- ***Viability:*** *Assessed by flow cytometry using antibodies against CD45, CD3, CD14, CD19, and CD56, with 7-aminoactinomycin D (7-AAD) as a viability dye. Antibodies included anti-CD3 (clone SK7, BD Biosciences, RRID: AB_2811220), anti-CD45 (clone 2D1, BD Biosciences, Cat# 655873, RRID: AB_2870390), anti-CD14 (clone M5E2, BD Biosciences, Cat# 345784, RRID: AB_2868810), anti-CD19 (clone 4G7, BD Biosciences, Cat# 345776, RRID: AB_2868804), and anti-CD56 (clone NCAM16.2, BD Biosciences, Cat# 345811, RRID: AB_2868832).*

- ***Purity:*** *Defined using the same flow cytometric panel as the proportion of viable CD45⁺ PBMCs relative to all viable cellular events, including granulocytes and events reflecting red blood cells or platelets.*
- ***Endotoxin:*** *Measured in accordance with Ph. Eur. 2.6.14 and USP <85> using a chromogenic Limulus amebocyte lysate assay (Endosafe® nexgen-PTS™, Charles River Laboratories).*

Post-Infusion Functional Characterization

Functional characterization was performed post-administration using retained DP samples. Because CBL-B function is most relevant following T-cell receptor (TCR) activation, functional characterization was performed after anti-CD3/CD28 stimulation rather than by comparing unstimulated CBL-B expression immediately after manufacturing. DP and matched no-pulse control (NPC) samples collected prior to electroporation were stimulated with anti-CD3/CD28 monoclonal antibodies (UltraLEAF anti-human CD3 [clone OKT3, RRID: AB_11147370] and UltraLEAF anti-human CD28 [clone 28.2, RRID: AB_11150591], BioLegend) for 1 hour and cultured for a total of 41–45 hours at 37°C and 5% CO_2 under identical conditions.

Supernatants and cell lysates were shipped on dry ice to a central laboratory (VelaLabs GmbH, Vienna, Austria) for analysis.

- ***Activity:*** *Defined as downregulation of CBL-B protein and messenger RNA (mRNA) in stimulated DPs relative to matched NPC samples, assessed by electrochemiluminescence-based immunoassay (protein) and quantitative reverse transcription polymerase chain reaction (RT-PCR) (mRNA).*
- ***Potency:*** *Defined as upregulation of IL-2 secretion in DP culture supernatants relative to NPC controls, measured using a commercially available electrochemiluminescence immunoassay (V-PLEX Plus Proinflammatory Panel 1, Meso Scale Diagnostics).*
- ***Sterility:*** *Assessed in accordance with Ph. Eur. 2.6.1 and USP <71> using validated membrane filtration or direct inoculation methods.*

Sample Collection and Processing

Peripheral blood samples were collected prior to each treatment cycle and ***at safety follow-up (SFU) and survival follow-up (SuFu), where applicable,*** *for exploratory pharmacodynamic and immune monitoring analyses. PBMCs and plasma were isolated using BD Vacutainer® CPT™ tubes with sodium heparin according to the manufacturer's instructions. PBMCs were cryopreserved in CryoStor® CS10, and plasma samples were stored at −80°C until analysis. Whole blood collected in lithium heparin tubes was additionally processed for direct flow cytometric evaluation following red blood cell lysis.*

Soluble Immune Mediator Profiling

Plasma samples were analyzed for soluble immune mediators, including soluble CD40L (sCD40L), granzyme B (GrB), interferon-γ (IFN-γ), IL-2, IL-10, tumor necrosis factor-α (TNF-α), and additional cytokines. Analyses were performed at VelaLabs GmbH (Vienna, Austria) using Olink® multiplex proteomics assays and Meso Scale Diagnostics electrochemiluminescence-based immunoassays. Cytokine concentrations were summarized descriptively and evaluated longitudinally relative to baseline.

Flow Cytometry–Based Immune Phenotyping

Immune phenotyping was performed using multiparameter flow cytometry on a CytoFLEX LX flow cytometer (Beckman Coulter, Germany). Antibody panels (BioLegend, Germany) were used to characterize major lymphoid and myeloid subsets, as well as activation-associated markers and inhibitory receptor expression. Markers included CD45, CD3, CD4, CD8, CD45RA, CD45RO, CCR7, CD62L, CD27, CD14, CD19, CD56, CD11b, CD15, CD33, HLA-DR, CD40L, CD137, PD-1, TIM-3, LAG-3, and related targets. Myeloid-derived suppressor cell (MDSC) subsets were defined according to established phenotypic criteria (Bronte et al., 2016): monocytic MDSCs (M-MDSCs), $CD11b^{+}CD14^{+}HLA\text{-}DR^{low}/CD15^{-}$; polymorphonuclear MDSCs (PMN-MDSCs), $CD14^{-}CD11b^{+}CD15^{+}$; and early-stage MDSCs (eMDSCs), lineage-neagtive (Lin^{-})($CD3^{-}CD14^{-}CD15^{-}CD19^{-}CD56^{-}$)$HLA\text{-}DR^{-}CD33^{+}$. Flow cytometry data were

analyzed using standardized gating strategies to define immune cell subsets and quantify population frequencies based on predefined gating hierarchies.

T-Cell Receptor Sequencing and Clonotype Analysis

T-cell receptor β (TCRβ) repertoire profiling was performed by CeGaT GmbH (Tübingen, Germany) to assess treatment-associated clonal dynamics. Sequencing was conducted using the Illumina® TCR Beta-SR Panel. Productive TCRβ clonotypes were identified, and clonal dynamics were evaluated longitudinally by tracking changes in clonotype frequency over time. Clonotype diversity and persistence were assessed descriptively.

Single-Cell RNA Sequencing

Single-cell ribonucleic acid (RNA) sequencing was performed on selected cryopreserved PBMC samples by Lexogen GmbH (Vienna, Austria) to characterize systemic immune changes associated with APN401 treatment. Single-cell 5′ gene-expression and T-cell V(D)J libraries were generated using the Chromium Next GEM Single Cell 5′ platform (10x Genomics), with a target recovery of 10,000 cells per sample. Libraries were sequenced on Illumina platforms. Primary processing, including demultiplexing, gene-expression quantification, and V(D)J analysis, was performed using Cell Ranger. Downstream quality control, normalization, dimensionality reduction, clustering, and integration were performed using Seurat. Cell-type annotation was performed using SingleR with the Human Primary Cell Atlas and Monaco Immune Data reference datasets. TCR clonotype information derived from V(D)J sequencing was integrated with the single-cell transcriptomic data to characterize the phenotypic identity and longitudinal persistence of expanded T-cell clonotypes.

Statistical Interpretation of Biomarker Analyses

Immune and clinical parameters were analyzed descriptively, and exploratory comparisons were performed to characterize treatment-associated immune changes and differences between clinical outcome groups. Effect size–based approaches were used to quantify the magnitude of observed

differences and prioritize signals for descriptive follow-up in the small cohort setting. Cohen's d was used as a standardized descriptive measure of between-group differences, with positive values indicating higher levels in patients with stable disease (SD) and negative values indicating higher levels in patients with progressive disease (PD). Absolute Cohen's d values of 0.2, 0.5, and 0.8 were considered small, moderate, and large effects, respectively. Baseline analyses compared pretreatment immune parameters between patients with SD and PD. Longitudinal analyses compared within-patient changes from baseline (pre-cycle 1) to post-cycle 1 and post-cycle 2 between the SD and PD groups. Because these analyses were exploratory and hypothesis-generating rather than confirmatory, no adjustment for multiple comparisons was applied, and effect sizes were not interpreted as evidence of statistical significance. Statistical analyses were performed using GraphPad Prism version 11.0 (RRID: SCR_002798).

***Supplemental Reference**:*

Bronte V, Brandau S, Chen SH, et al. Recommendations for myeloid-derived suppressor cell nomenclature and characterization standards. Nat Commun. 2016;7:12150. doi:10.1038/ncomms12150.
