# Supplementary material for: Phase 1 evaluation of APN401, an autologous CBL-B-silenced PBMC therapy, in advanced solid tumors
Source: Mol Ther Oncol. 2026 Oct 5;34(4):201357. doi: 10.1016/j.omton.2026.201357 (PMC13717735; doi:10.1016/j.omton.2026.201357)
Supplement: Supplementary file 2 [file mmc2.pdf]

Molecular Therapy
Oncology
Original Article

Phase 1 evaluation of APN401, an autologous CBL-B-silenced PBMC therapy, in advanced solid tumors

Alexander Dohnal,[1,8] Nina Worel,[2,8] Thomas Bogenrieder,[1] Thorsten Fuereder,[3] Martin Burian,[4] Dominik Wolf,[5] Gerald Prager,[3] Richard Greil,[6] Anja Peterbauer-Scherb,[7] Beate Schrenk,[2] Stefan Bunka,[1,2] Ernesto Abila,[1,2] Felix Batrina,[2] Maria Urban,[1] Julia Buchberger,[1] Alice Schwarzboeck,[1] Bernhard Peball,[1] Sandra Seidl,[1] Hannes Muehleisen,[1] Stefan Stranner,[1] Mario Kuttke,[1] Angela Halfmann,[1] Sarah Bischof,[1] Andreas Birbach,[1] and Romana Gugenberger[1]

[1]InvIOs GmbH, Campus Vienna Biocenter 5, 1030 Vienna, Austria; [2]Medical University of Vienna, Department of Transfusion Medicine and Cell Therapy, Waehringer Guertel 18-20, 1090 Vienna, Austria; [3]Medical University of Vienna, Department of Medicine I, Division of Oncology, Waehringer Guertel 18-20, 1090 Vienna, Austria; [4]Ordensklinikum Linz GmbH, Barmherzige Schwestern, Seilerstaette 4, 4020 Linz, Austria; [5]Medical University Innsbruck, Department of Internal Medicine V, Hematology and Oncology, Anichstrasse 35, 6020 Innsbruck, Austria; [6]Salzburg Cancer Research Institute, Center for Clinical Cancer and Immunology Trials, Paracelsus Medical University Salzburg, Muellner Hauptstrasse 48, 5020 Salzburg, Austria; [7]Austrian Red Cross, Blood Transfusion Service for Upper Austria, Krankenhaustrasse 7, 4010 Linz, Austria

Intracellular immune checkpoints regulate activation thresholds in cytotoxic immune cells. Casitas B-lineage lymphoma proto-oncogene B (CBL-B) is an E3 ubiquitin ligase that negatively regulates antigen receptor signaling and T cell activation. APN401 is an autologous peripheral blood mononuclear cell (PBMC) therapy incorporating transient small interfering RNA (siRNA)-mediated CBL-B silencing. Two phase 1 studies evaluated escalating doses of APN401 in patients with advanced treatment-refractory solid tumors. APN401 was manufactured from leukapheresis-derived PBMCs using the EPiC point-of-care platform. Seventeen patients received APN401 across three dose levels. APN401 demonstrated a favorable safety profile, with no dose-limiting toxicities, cytokine release syndrome, immune effector cell-associated neurotoxicity syndrome, or grade ≥3 treatment-related adverse events. Manufacturing was reproducible across sites. Although no objective responses were observed, disease stabilization occurred in 5 of 10 Response Evaluation Criteria in Solid Tumors (RECIST)-evaluable patients (50.0%), corresponding to 29.4% of all 17 treated patients. Exploratory analyses identified treatment-associated systemic immune changes, including enrichment of circulating $CD8^+$ T cells, induction of cytotoxic mediators, and expansion of dominant T cell receptor β (TCRβ) clonotypes. These changes were most pronounced in patients with stable disease but remain exploratory and hypothesis-generating. These findings support further evaluation of APN401 in larger, controlled studies.

INTRODUCTION

Immunotherapy has transformed cancer treatment, with immune checkpoint inhibitors producing durable clinical benefit across multiple tumor types. However, many patients with advanced malignancies fail to achieve sustained responses due to immunosuppressive tumor microenvironments, antigen heterogeneity, and progressive dysfunction of tumor-reactive T cells.[1–5] Additional immunotherapeutic approaches, including cellular therapies, have demonstrated activity in selected settings but remain limited in solid tumors by impaired tumor infiltration, antigen escape, and manufacturing complexity.[6–8] These challenges highlight the need for complementary strategies that enhance intrinsic immune cell function.

Intracellular signaling pathways regulating activation thresholds represent an emerging target space for immune modulation. Casitas B-lineage lymphoma proto-oncogene B (CBL-B), an E3 ubiquitin ligase, is a key negative regulator of antigen receptor signaling that increases the threshold for T cell activation.[9,10] Preclinical studies have shown that CBL-B inhibition enhances T cell activation and promotes antitumor immunity.[11,12] Furthermore, CBL-B regulates both adaptive and innate immune-cell function, including T and natural killer (NK) cells, providing a rationale for therapeutic CBL-B inhibition across multiple immune-cell compartments.[9,13]

Received 11 June 2026; accepted 2 October 2026;
https://doi.org/10.1016/j.omton.2026.201357.

[8]These authors contributed equally

Correspondence: Alexander Dohnal, InvIOs GmbH, Campus Vienna Biocenter 5, 1030 Vienna, Austria.
E-mail: correspondence@invios.com
Correspondence: Nina Worel, Medical University of Vienna, Department of Transfusion Medicine and Cell Therapy, Waehringer Guertel 18-20, 1090 Vienna, Austria.
E-mail: nina.worel@meduniwien.ac.at

© 2026 The Authors. Published by Elsevier Inc. on behalf of The American Society of Gene and Cell Therapy.
This is an open access article under the CC BY license (http://creativecommons.org/licenses/by/4.0/).

To translate this concept clinically, APN401 was developed as an autologous peripheral blood mononuclear cell (PBMC)-based therapy incorporating transient small interfering RNA (siRNA)-mediated silencing of CBL-B. Early phase 1 clinical evaluation of APN401 in patients with advanced solid tumors demonstrated the feasibility of *ex vivo* siRNA-mediated CBL-B silencing in autologous PBMCs and tolerability of APN401 administration.[14,15] These findings provided the basis for evaluating repeated APN401 administration and a rapid point-of-care manufacturing approach in subsequent clinical development. Along these lines, APN401 was manufactured using the enhancement platform for immune cells (EPiC), enabling same-day production of modified, autologous PBMC products from leukapheresis-derived cells.

Here, we report results from two phase 1 studies evaluating APN401 in a broad population of patients with advanced treatment-refractory solid tumors, reflecting the safety and feasibility objectives of early-phase development rather than patient selection based on established tumor-specific sensitivity to CBL-B inhibition in immune cells. Hence, the studies focused on safety, manufacturing feasibility, and exploratory immune activity associated with transient intracellular checkpoint modulation.

RESULTS

APN401 manufacturing and proposed mechanism of action

The APN401 manufacturing workflow and proposed mechanism of action are summarized in Figure 1. APN401 is generated from autologous leukapheresis-derived PBMCs using the EPiC point-of-care manufacturing platform (Figure 1A). Following PBMC purification, unfractionated PBMCs are electroporated with CBL-B-targeting siRNA, formulated as APN401, subjected to pre-infusion release testing, and reinfused into the patient within a vein-to-vein time of <24 h. Post-infusion functional characterization is performed on retained drug-product (DP) samples following T cell receptor (TCR) stimulation. Following reinfusion, antigen encounter and TCR activation are expected to induce CBL-B expression as part of physiological negative-feedback signaling. The transfected CBL-B-specific siRNA limits this activation-induced CBL-B expression, thereby transiently reducing negative regulation of TCR signaling and enhancing T cell activation, cytokine production, proliferation, and cytotoxic function (Figure 1B).

Patients and treatments

Nineteen patients were screened across APN401-103 and APN401-104, and 17 patients received at least one APN401 infusion and comprised the pooled safety population (Table 1; Tables S1 and S2). Patients represented a heterogeneous population of advanced treatment-refractory solid tumors and had received at least two prior lines of systemic therapy. In APN401-104, patients received a median of 4 prior lines of therapy (range, 3–8), and 6 patients had prior exposure to immune checkpoint inhibitors. Patients received escalating doses ranging from 5×10^6 to 4.5×10^7 PBMCs/kg and completed a median of 3 treatment cycles (range, 1–6). Dose escalation in APN401-103 evaluated dose levels 1 (5×10^6 PBMCs/kg) and 2 (1.5×10^7 PBMCs/kg), whereas APN401-104 evaluated dose levels 2 and 3 followed by expansion at dose level 3 (4.5×10^7 PBMCs/kg), which was subsequently selected as the recommended phase 2 dose (RP2D). Treatment discontinuation primarily reflected disease progression or clinical deterioration in this heavily pretreated population. Additional reasons included withdrawal in the patient's best interest (n = 4), patient withdrawal (n = 1) of consent, and out-of-specification (OOS, n = 2) manufacturing batches identified before infusion (Table S2). Overall, 51 of 56 manufactured batches met pre-infusion release specifications (Figure S1). APN401 was manufactured and administered as a fresh, non-cryopreserved cell product without an intervening cryopreservation and thawing step. Of the five OOS batches, two did not meet the predefined identity specification and three did not meet the predefined quantity specification. These events did not result in modification of the APN401 manufacturing process but informed subsequent narrowing of the acceptance specification for the leukapheresis starting material. Post-electroporation DP viability was 76.4% ± 7.2% of viable PBMCs, and functional characterization demonstrated reduction of *CBLB* mRNA by 56.0% ± 14.2% and reduction of CBL-B protein expression by 91.4% ± 10.7% relative to matched controls.

Safety and tolerability

All treated patients (n = 17) were included in the safety population. APN401 demonstrated a favorable safety profile, with no dose-limiting toxicities (DLTs), cytokine release syndrome, immune effector cell-associated neurotoxicity syndrome (ICANS), grade ≥3 treatment-related adverse events (TRAEs), treatment-related serious adverse events (AEs), or treatment-related deaths. AEs reported in ≥3 patients are summarized in Table 2. Most events were grade 1–2 and attributable to study procedures or underlying disease. TRAEs primarily included transient infusion-associated symptoms such as fatigue, pyrexia, nausea, dizziness, and headache. No patients discontinued treatment because of treatment-related toxicity.

Determination of the RP2D

Three dose levels were evaluated: dose level 1 (n = 3), dose level 2 (n = 7), and dose level 3 (n = 7) (Table S2). No DLTs occurred at any dose level. Following Bayesian optimal interval (BOIN) analysis and isotonic regression adjustment, the estimated probability of toxicity remained below the prespecified target toxicity rate of 25% across all dose levels. Based on the favorable tolerability profile, dose level 3 (4.5×10^7 PBMCs/kg) was selected as the RP2D. A maximum tolerated dose was not reached within the dose range evaluated. RP2D selection was based on the phase 1 dose-escalation and safety assessment and was not based on clinical response rates.

Clinical activity

Best overall response (BOR) according to RECIST v.1.1[16] was evaluable in 10 of 17 treated patients (Tables S3 and S2). Seven patients were not evaluable for BOR because of early treatment termination, including two following OOS manufacturing batches, four best-interest withdrawals, and one patient withdrawal. No objective responses were observed. Stable disease (SD) occurred in 5 of 10

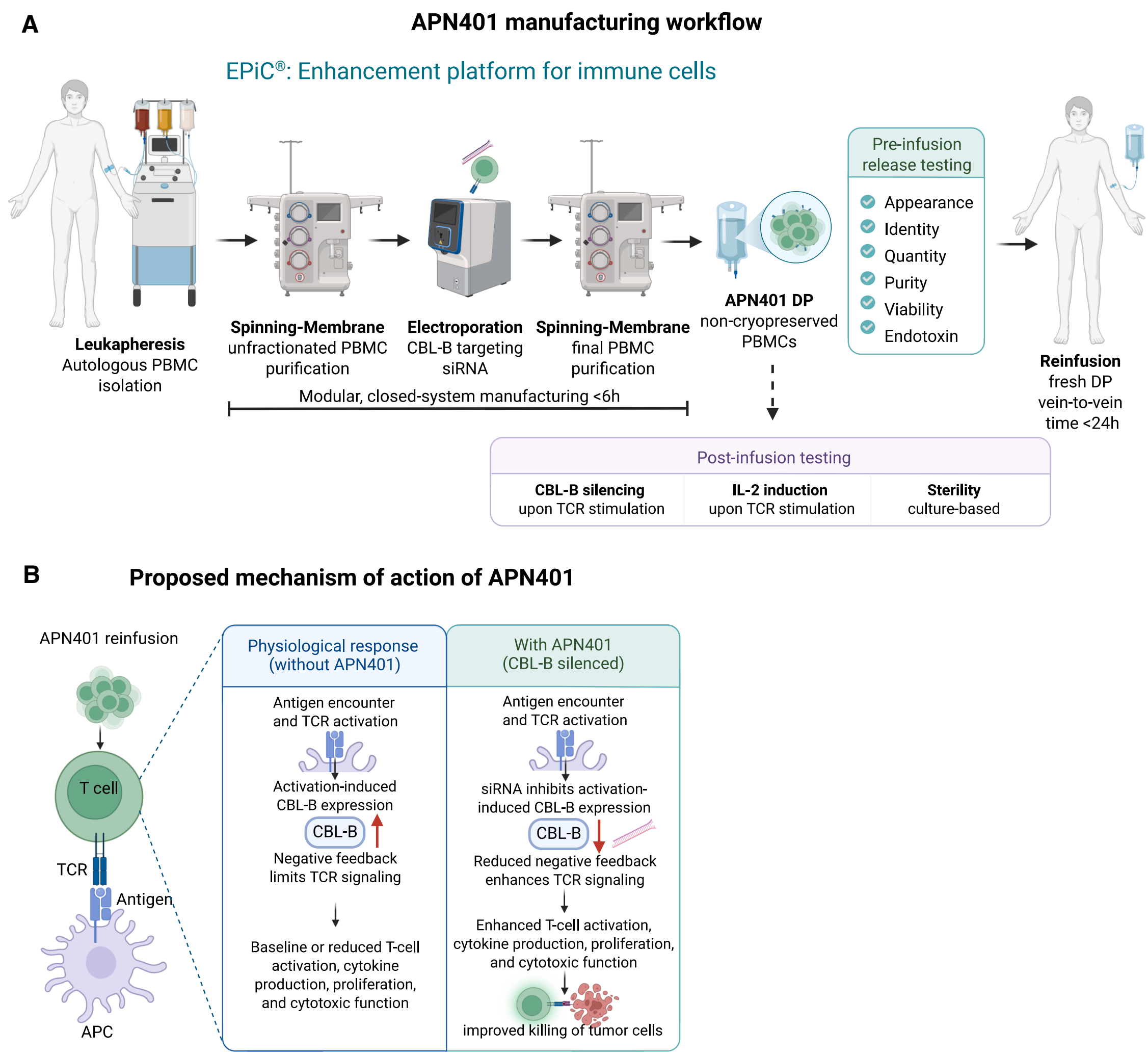

Figure 1. APN401 manufacturing workflow and proposed mechanism of action

(A) APN401 is manufactured from autologous peripheral blood mononuclear cells (PBMCs) collected by leukapheresis. Following closed-system PBMC purification, unfractionated PBMCs are electroporated with CBL-B-targeting siRNA, followed by removal of cell debris and residual reagents and final formulation of APN401. The drug-product (DP) undergoes pre-infusion release testing for appearance, identity, quantity (cell count), purity, viability, and endotoxin before reinfusion, with a vein-to-vein time of <24 h. Sterility and functional characterization of retained DP samples is performed post-infusion and includes assessment of *CBLB* mRNA knockdown and CBL-B protein downregulation and IL-2 induction following T cell receptor (TCR) stimulation. (B) Proposed mechanism of action of APN401. Following reinfusion, antigen encounter, and TCR activation are expected to induce CBL-B expression as part of physiological negative-feedback signaling. CBL-B-specific siRNA introduced during APN401 manufacturing limits activation-induced CBL-B expression, thereby transiently reducing negative regulation of TCR signaling and enhancing T cell activation, cytokine production, proliferation, and cytotoxic function, which may improve recognition and killing of tumor cells.

APC, antigen-presenting cell; CBL-B, Casitas B-lineage lymphoma proto-oncogene B; siRNA, small interfering RNA; TCR, T cell receptor. Created in BioRender. Kuttke, M. (2026) https://BioRender.com/qzshtzm.

evaluable patients, corresponding to a disease control rate (DCR) of 50.0%, or 29.4% when calculated across all 17 treated patients, whereas the remaining RECIST-evaluable patients experienced progressive disease (PD). Disease stabilization was most frequently observed at dose level 2. Given the small number of RECIST-evaluable patients within each dose level, these findings do not establish a dose-response relationship for clinical activity. Tumor response kinetics and treatment exposure are summarized in Figures 2 and 3. Most treatment discontinuations and deaths were attributable to disease progression.

One patient with appendiceal carcinoma (103-01-005) demonstrated prolonged disease stabilization and remained alive 4 years after treatment initiation. The patient received subsequent chemotherapy after radiographic progression during extended APN401 treatment; therefore, the prolonged clinical course cannot be attributed to

Table 1. Patient demographics and disease characteristics

Parameter	APN401-103	APN401-104
**Number of patients receiving APN401 (*n*)**	6	11
**Age (years)**		
Minimum-maximum	59–71	50–79
Mean (SD)	64.7 (4.23)	62.5 (8.21)
Median	65.5	60.0
**Race/ethnicity (*n*)**		
White	6	11
Asian	0	0
Other	0	0
**Sex (*n*)**		
Male	4	6
Female	2	5
**ECOG performance status at baseline (*n*)**		
0–2 (eligibility criterion)	6	11
**Previous lines of systemic therapy**		
≥2 prior lines (eligibility criterion)	6 (100.0)	11 (100.0)
**Tumor type (*n*)**		
Colorectal carcinoma	1	1
Head and neck squamous cell carcinoma (HNSCC)	2	3
Pancreatic carcinoma	2	0
Appendiceal carcinoma	1	0
Non-small-cell lung cancer (NSCLC)	0	1
Small-cell lung cancer (SCLC)	0	1
Cholangiocarcinoma	0	1
Uveal melanoma	0	3
Melanoma	0	1

Table 2. Adverse events reported in ≥3 patients

Adverse event	Patients, *n* (%)
Implant site hematoma	11 (64.7%)
Fatigue	10 (58.8%)
Abdominal pain	6 (35.3%)
General physical health deterioration	5 (29.4%)
Ascites	4 (23.5%)
Constipation	4 (23.5%)
Nausea	4 (23.5%)
Dyspepsia	4 (23.5%)
Implant site hemorrhage	3 (17.6%)
Implant site pain	3 (17.6%)
Pyrexia	3 (17.6%)
Arthralgia	3 (17.6%)
Dyspnea	3 (17.6%)
Myalgia	3 (17.6%)
Anemia	3 (17.6%)

Adverse events are shown irrespective of relationship to APN401.

APN401 alone. Another patient with recurrent/metastatic head and neck squamous cell carcinoma (HNSCC) (104-02-005) discontinued treatment after two cycles following clinical progression and withdrawal in the patient's best interest; however, follow-up imaging approximately 170 days after the first infusion demonstrated a reduction in target lesions and resolution of previously detected lesions. No intervening anticancer therapy was administered during this interval. Because iRECIST criteria were not prospectively implemented, this delayed radiographic finding cannot be classified as pseudoprogression or definitively attributed to APN401.

Immune biomarker analyses

Exploratory immune biomarker analyses were performed to characterize systemic immune changes associated with APN401 treatment and to evaluate immune features observed in patients with disease stabilization. Given the limited sample size, these analyses were descriptive and hypothesis-generating and were not intended to establish statistical significance. Longitudinal analyses were performed using paired samples collected at baseline (pre-cycle 1), post-cycle 1, and post-cycle 2. Baseline samples were obtained before APN401 manufacturing and therefore reflected the untreated immune state.

Treatment-associated immune activation

Comparison of baseline and post-cycle 1 samples demonstrated early modulation of multiple immune parameters (Figure 4A), including changes in circulating $CD8^+$ T cell populations, memory-associated T cell subsets, activation-associated markers, and soluble immune mediators. Post-cycle 2 analyses demonstrated continued immune remodeling across lymphoid and myeloid compartments (Figures 4B and 4C), including changes in $CD8^+$ T cell abundance, $CD8^+/CD4^+$ T cell ratios, B cell populations, monocyte subsets, and myeloid-derived suppressor cell (MDSC)-associated parameters. Longitudinal analyses also demonstrated delayed upregulation of inhibitory receptors including PD-1, TIM-3, and LAG-3 during treatment (Figure S2). Patients with SD demonstrated longitudinal changes in markers associated with cytotoxic T cell activation and systemic immune remodeling, including increased $CD137^+$ and $TIM\text{-}3^+$ $CD8^+$ T cells, elevated plasma interferon-γ (IFN-γ), and expansion of circulating B cell populations. In contrast, PD was associated with higher monocyte levels and increased plasma IL-10. These immune changes coincided with expansion of dominant TCRβ clonotypes in selected patients (Figure S3). The antigen specificity of these expanded clonotypes was not determined.

Baseline immune features associated with clinical outcome

Baseline immune profiles were analyzed to explore pretreatment immune features associated with clinical outcome (Figures 5 and S4). Patients with PD demonstrated higher baseline levels of several T cell subsets including $CD8^+$ T cells and plasma granzyme B

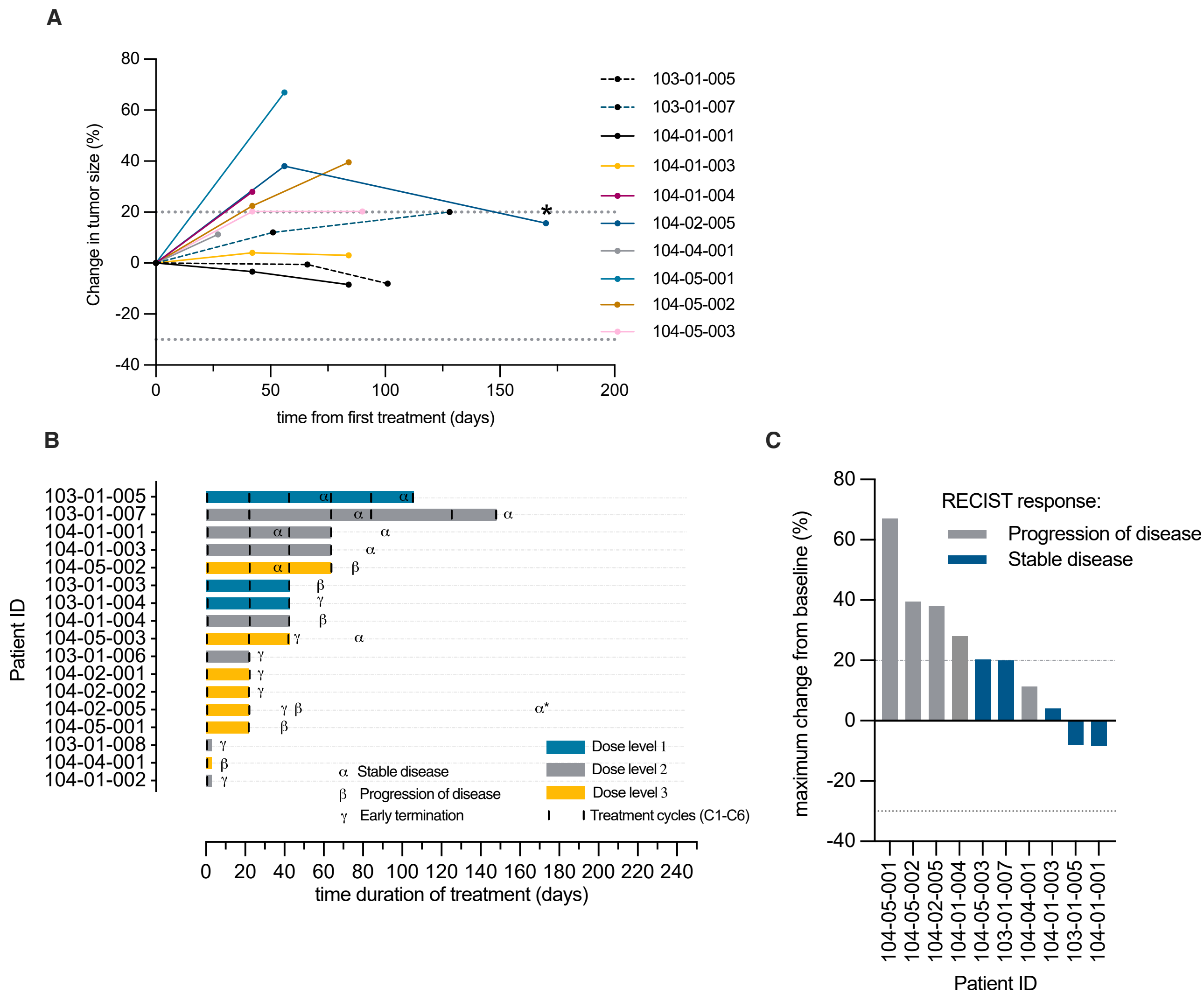

Figure 2. Tumor response and treatment exposure following APN401 therapy

(A) Longitudinal percentage change in target-lesion size from baseline in patients with available target-lesion measurements (n = 10). Dashed lines indicate RECIST v.1.1 thresholds for partial response (−30%) and progressive disease (+20%). (B) Swimmer plot showing treatment exposure and clinical course in the safety population (n = 17). Each bar represents an individual patient and indicates the duration of APN401 treatment. Symbols indicate stable disease (α), progressive disease (β), or early termination (γ). *Reduction in target lesion measurements observed during follow-up. (C) Maximum percentage change in target-lesion size from baseline in patients with available target-lesion measurements (n = 10). Each bar represents an individual patient; colors indicate stable disease or progressive disease according to RECIST v.1.1. BOR, best overall response; RECIST, Response Evaluation Criteria in Solid Tumors.

(GrB), whereas patients with SD demonstrated higher baseline plasma IFN-γ and increased frequencies of selected MDSC subsets. Given the small sample size and exploratory nature of these analyses, these baseline differences should be considered descriptive and hypothesis-generating.

Representative patients with immune correlates

Two patients with extended longitudinal immune monitoring are shown to illustrate heterogeneous immune response kinetics observed during APN401 treatment.

Patient 103-01-005 had metastatic appendiceal carcinoma and had received 11 prior lines of therapy before enrollment. Patient 103-01-005 received six cycles of APN401 at dose level 1 (5×10^6 PBMCs/kg) and achieved SD during the protocol-defined treatment period, with largely stable target lesion measurements on serial imaging (Figure 3). Following study completion, the patient received six additional APN401 cycles through a named-patient use program (total n = 12). Although radiographic progression occurred during extended treatment, disease stabilization was reestablished after APN401 discontinuation and subsequent chemotherapy. The patient remained alive and clinically stable at both 2- and 4-year survival

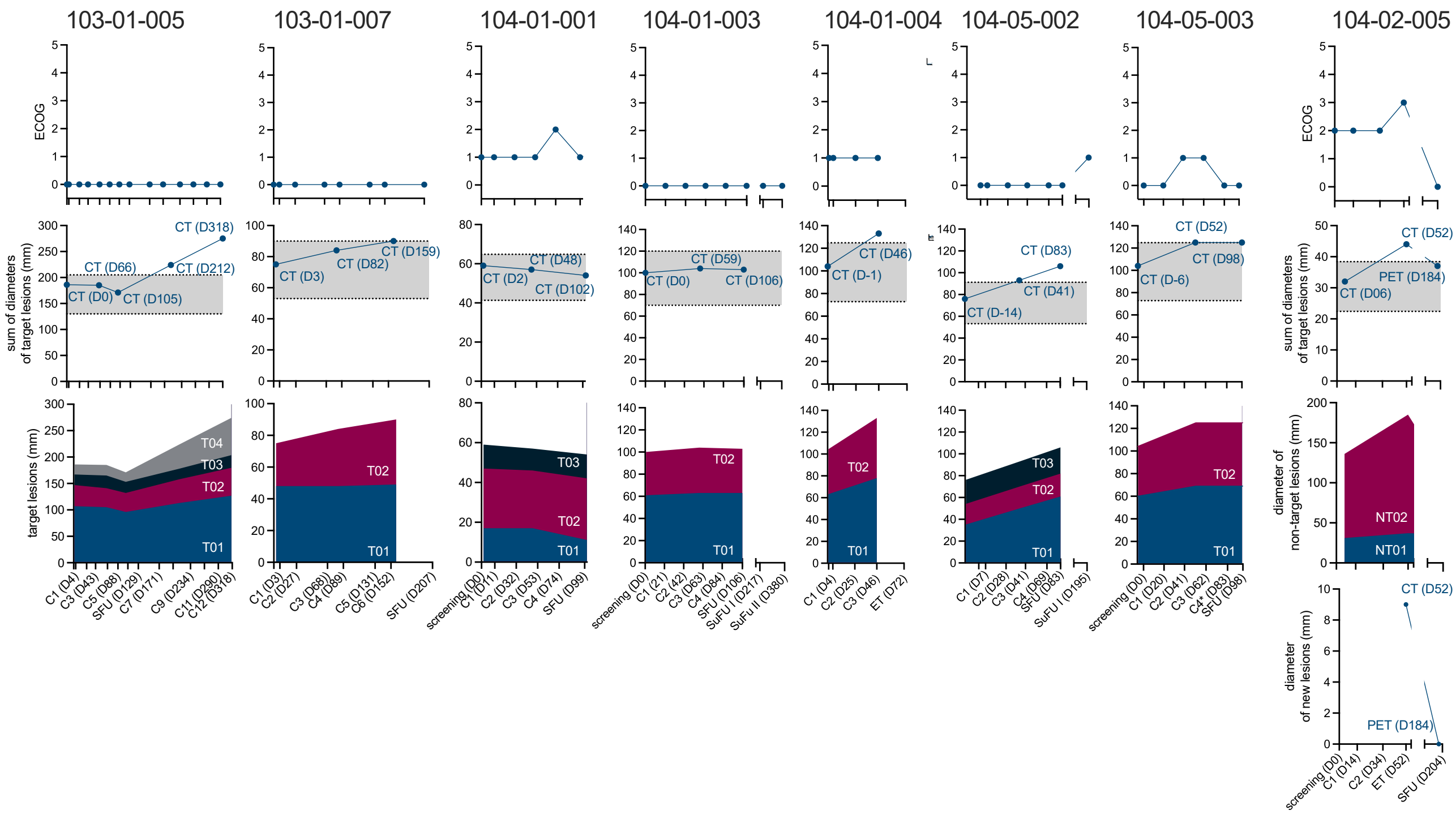

Figure 3. Clinical and radiographic assessments in selected APN401-treated patients

Clinical status and radiographic tumor assessments were evaluated longitudinally in selected patients with sufficient follow-up to assess longitudinal tumor kinetics. ECOG performance status and target lesion measurements were collected at screening, during treatment cycles, and during follow-up assessments. Stacked plots show the sum of target lesion (T) measurements over time. Non-target lesions (NT) and new lesions are indicated where applicable. Tumor assessments were performed according to RECIST v.1.1 criteria. Patient 104-05-003 did not receive cycle 4 because no DP was manufactured.

follow-up (SuFu) visits. Given the intervening chemotherapy, the contribution of APN401 to the subsequent prolonged clinical course cannot be determined.

Functional characterization of APN401 DPs demonstrated sustained activity across manufacturing batches (Figure S5). Following anti-CD3/CD28 stimulation, DPs consistently showed increased secretion of IL-2, CD137, IFN-γ, and CXCL13 compared with matched non-CBL-B-silenced controls across cycles C1-C5, indicating reproducible enhancement of T cell activation after CBL-B silencing.

Longitudinal immune monitoring revealed dynamic modulation of circulating immune mediators during treatment, including early increases in plasma GrB and transient induction of IFN-γ and soluble CD40L (sCD40L), consistent with enhanced cytotoxic activity and T cell-antigen-presenting cell interactions (Figures 6A and S5). Peripheral immune phenotyping demonstrated early enrichment of circulating $CD8^+$ T cells and an increased $CD8^+/CD4^+$ T cell ratio (Figure S3). TCRβ sequencing revealed expansion of multiple dominant clonotypes during treatment, several of which persisted at the 2-year SuFu visit (Figure 6B). Single-cell RNA sequencing localized these clonotypes to $CD8^+$ effector and central memory T cell subsets (Figure S6), demonstrating persistence of these clonotypes within $CD8^+$ T cell populations, although their tumor specificity was not established.

Patient 104-02-005 had recurrent/metastatic HNSCC and had received three prior lines of systemic therapy. This patient received two APN401 cycles at the (RP2D; 4.5×10^7 PBMCs/kg) before treatment discontinuation following clinical progression and withdrawal in the patient's best interest. No intervening anticancer therapy was administered between APN401 discontinuation and follow-up imaging performed 170 days after the first infusion, which demonstrated a reduction in target lesions (Figures 2 and 3). Because iRECIST was not prospectively implemented, this delayed radiographic finding cannot be classified as pseudoprogression or definitively attributed to APN401.

Plasma analysis revealed marked induction of immune activation markers after a single treatment cycle, including GrB, sCD40L, and IFN-γ (Figure 6C). Peripheral immune phenotyping demonstrated enrichment of circulating $CD4^+$ and $CD8^+$ T cells (Figure S3). In parallel, TCRβ sequencing identified expansion of dominant clonotypes following treatment, with several clones increasing from baseline and becoming highly enriched at survival follow-up (SuFu; day 204 after screening) (Figures 6D and S3). These clonal expansions coincided with $CD8^+$ T cell

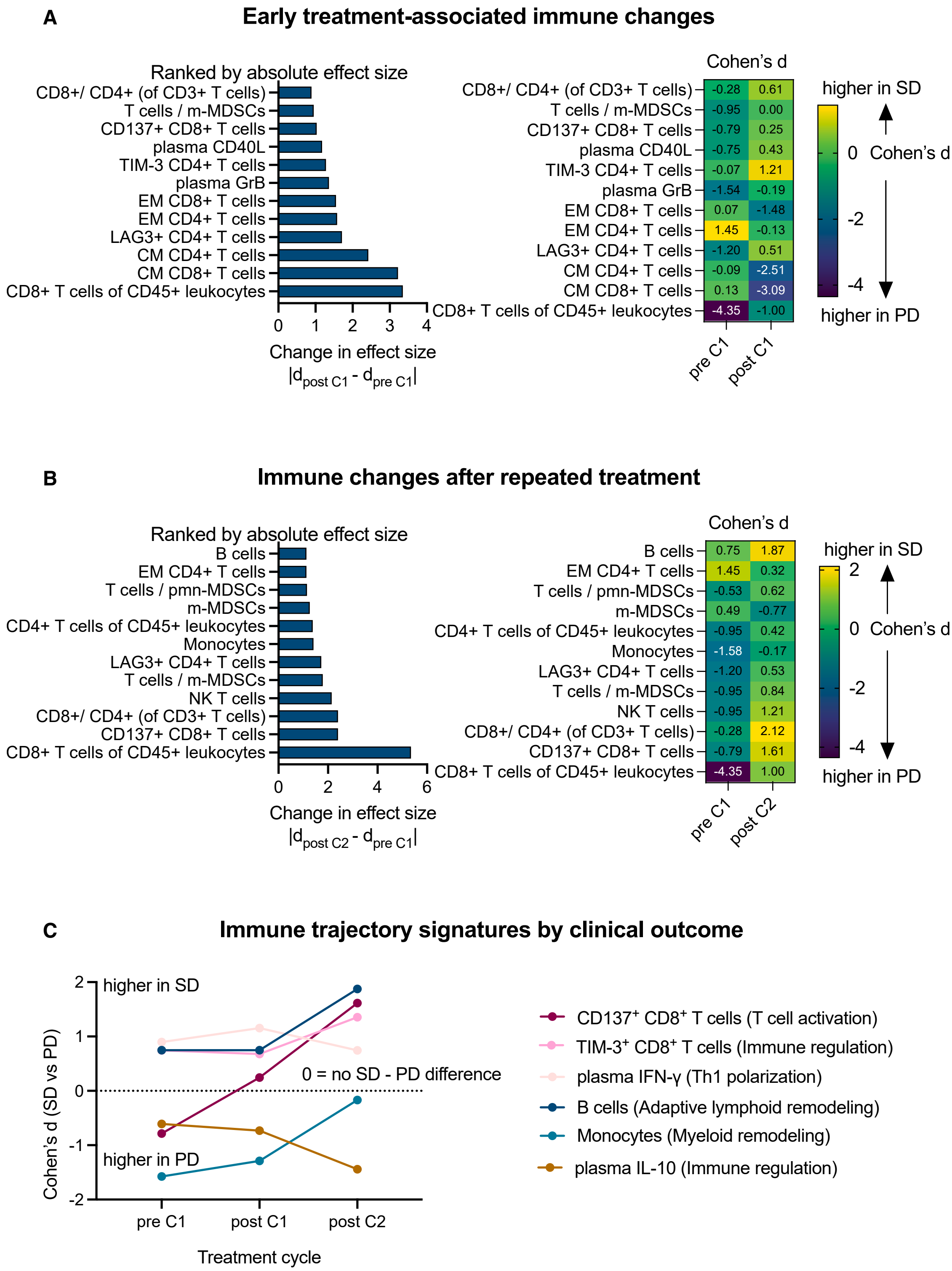

Figure 4. Longitudinal immune marker dynamics during APN401 treatment

Peripheral immune markers were analyzed longitudinally in patients with disease stabilization (SD-associated group, $n = 4$) and progressive disease (PD, $n \geq 3$) in study APN401-104. (A and B) Bars rank immune markers by the absolute change in Cohen's *d* from baseline (pre-C1) to post-cycle 1 (A) or post-cycle 2 (B); adjacent heatmaps show Cohen's *d* at each time point. (C) Temporal trajectories of Cohen's *d* for representative immune markers. Positive values indicate higher levels in SD and negative values higher levels in PD. Immune subsets include lymphoid populations and monocytic (m)- and polymorphonuclear (pmn)-myeloid-derived suppressor cells (MDSCs).

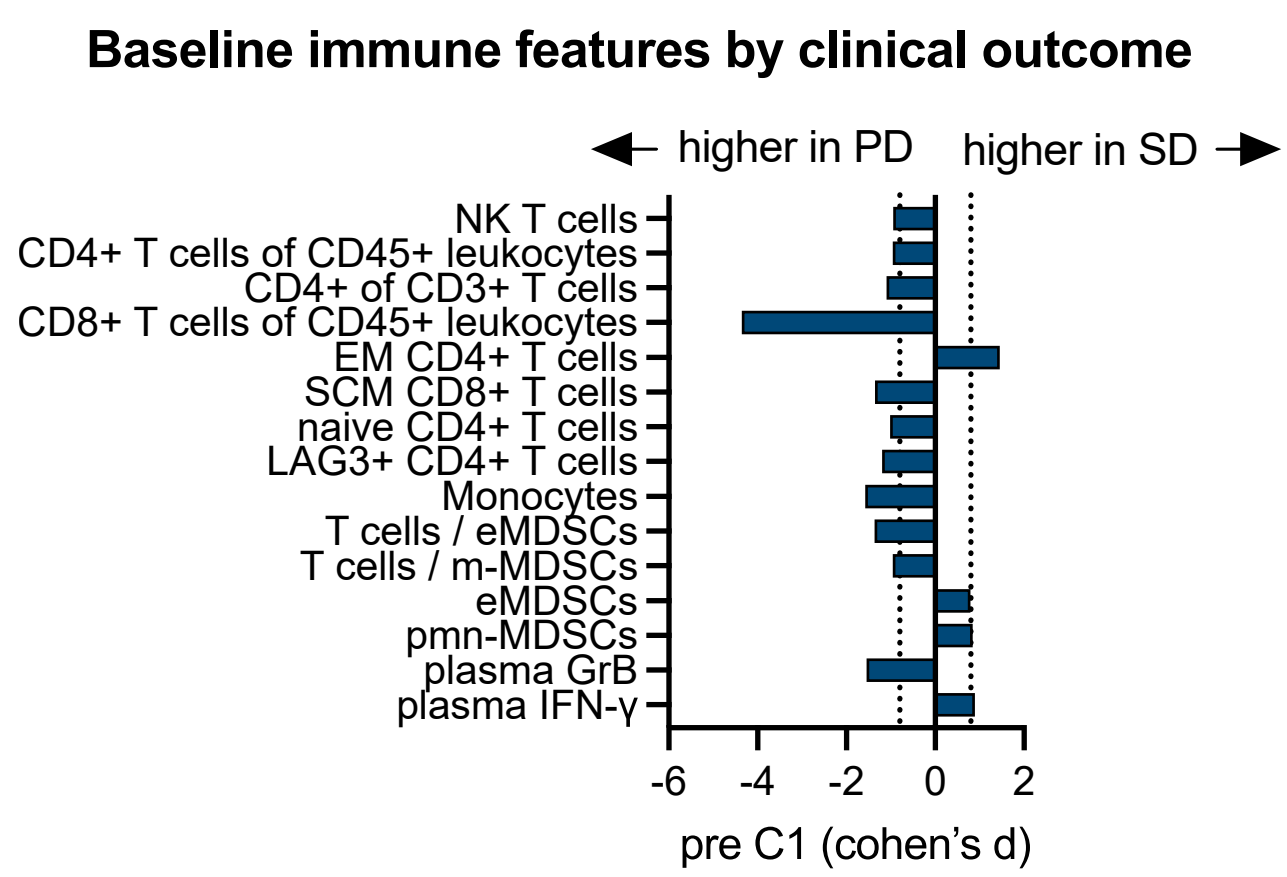

Figure 5. Baseline immune features associated with clinical outcome

Baseline immune markers measured prior to APN401 treatment (pre-C1) were compared between patients with disease stabilization (SD, n = 4) and progressive disease (PD, n = 4) in study APN401-104. Bars represent standardized effect sizes (Cohen's d, SD vs. PD). Positive values indicate markers enriched in SD, whereas negative values indicate markers enriched in PD. The dashed line indicates strong effect size (|Cohen's d| $\geq$ 0.8). Immune subsets include lymphoid populations and monocytic (m)- and polymorphonuclear (pmn)- myeloid-derived suppressor cell (MDSC) subsets.

enrichment and preceded the delayed reduction in target lesions observed on positron emission tomography (PET)-computed tomography (CT) imaging. The tumor specificity of the expanded clonotypes was not established, and their temporal association with the subsequent radiographic finding does not demonstrate a causal relationship.

Together, these cases illustrate heterogeneous longitudinal immune changes observed following APN401 treatment. In patient 103-01-005, immune activation and persistent T cell clonotypes were observed during longitudinal follow-up, whereas in patient 104-02-005, early immune changes and clonal T cell expansion preceded the later reduction in target lesion measurements. Given the intervening chemotherapy in patient 103-01-005 and the limitations of interpreting post-treatment imaging in patient 104-02-005, these observations are descriptive and do not establish a causal relationship between APN401-associated immune changes and clinical outcome.

Collectively, APN401 demonstrated feasible decentralized manufacturing, favorable tolerability, and exploratory evidence of systemic immune modulation in patients with advanced solid tumors. Although objective responses were not observed, disease stabilization and treatment-associated immune changes were identified in a subset of patients.

DISCUSSION

Immunotherapy has transformed cancer treatment, yet many patients with advanced solid tumors fail to achieve durable benefit because of immunosuppressive tumor microenvironments, antigen heterogeneity, and progressive T cell dysfunction.[1–5] These limitations highlight the need for complementary strategies that enhance immune responses beyond established surface checkpoint pathways. Targeting intracellular immune checkpoints represents a complementary approach by modulating activation thresholds downstream of antigen receptor signaling.[9] CBL-B is a key negative regulator of T cell activation that physiologically increases the threshold for antigen-receptor signaling and contributes to the maintenance of peripheral immune tolerance.[9,10] Accordingly, permanent loss of CBL-B function enhances immune-cell activation and antitumor immunity but has also been associated with increased susceptibility to autoimmunity in experimental models.[10–12] More recent unbiased genome-wide CRISPR screening in primary human T cells independently identified CBL-B among intracellular regulators whose disruption conferred resistance across multiple immunosuppressive conditions relevant to the tumor microenvironment, further supporting CBL-B as a therapeutic intracellular checkpoint.[17] Transient modulation may therefore provide an opportunity to enhance immune-cell responsiveness while avoiding permanent genetic disruption of this physiologically important regulatory pathway. APN401 was developed on this principle as an autologous PBMC-based therapy incorporating transient siRNA-mediated CBL-B silencing. Manufacturing using the EPiC platform was consistently feasible, enabling rapid ($\sim$6 h), decentralized, point-of-care production. APN401 was administered as a fresh, non-cryopreserved PBMC product, avoiding an intervening cryopreservation and thawing step within this rapid manufacturing workflow. In contrast to conventional autologous cell therapies limited by manufacturing complexity, capacity constraints, and prolonged vein-to-vein times,[18,19] this approach supports repeated dosing and may preserve cell fitness by avoiding extended *ex vivo* culture.[6–8] The reproducibility across sites supports its scalability as a practical delivery strategy. Allogeneic cell therapies aim to overcome logistical constraints through off-the-shelf availability and standardized manufacturing but face challenges including host-mediated rejection, limited persistence, and the need for genetic modification to control alloreactivity.[20] In heterogeneous solid tumors, autologous approaches may additionally preserve endogenous tumor-reactive immune repertoires. In this context, APN401 combines preservation of the endogenous immune repertoire with rapid point-of-care manufacturing.

APN401 demonstrated a favorable safety profile, with no DLTs, cytokine release syndrome, ICANS, or grade $\geq$3 TRAEs. Compared with tumor-infiltrating lymphocyte (TIL) and engineered T cell therapies, which are frequently associated with substantial toxicities,[6–8] these findings suggest that transient intracellular checkpoint silencing may provide a comparatively manageable safety profile, although cross-trial comparisons remain limited. The use of unfractionated PBMCs preserves immune-cell populations beyond T cells, and CBL-B is known to negatively regulate activation signaling in several leukocyte subsets, including NK cells, B cells, and myeloid cells.[9,13] However, the functional contribution of these non-T cell populations to APN401 activity was not specifically assessed in the present studies. Along these lines, APN401

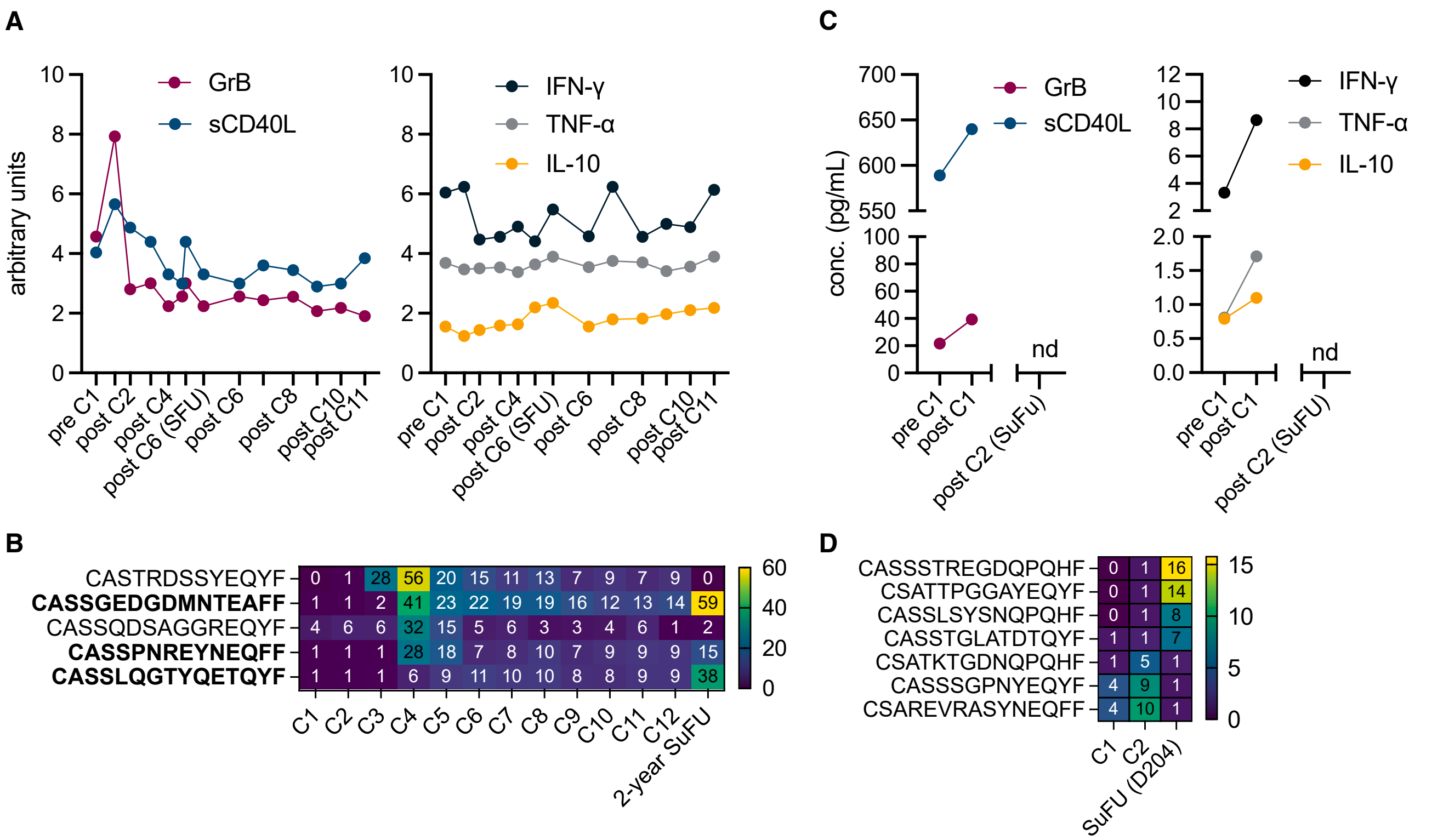

Figure 6. Longitudinal immune monitoring in representative APN401-treated patients

(A and B) Patient 103-01-005 (appendiceal carcinoma), who achieved disease stabilization during protocol-defined APN401 treatment and underwent longitudinal immune monitoring through survival follow-up. (A) Longitudinal plasma levels of interferon-γ (IFN- γ), tumor necrosis factor-α (TNF- α), granzyme B (GrB), soluble CD40L (sCD40L), and IL-10, expressed in arbitrary units, at baseline (pre-C1) and subsequent treatment and follow-up time points. (B) TCRβ repertoire profiles across treatment cycles C1-C12 and at 2-year survival follow-up (SuFu).

(C and D) Patient 104-02-005 (recurrent/metastatic head and neck squamous cell carcinoma), who discontinued APN401 following clinical progression and withdrawal in the patient's best interest and subsequently showed a reduction in target-lesion measurements during post-treatment follow-up. (C) Plasma concentrations of IFN- γ, TNF-α, GrB, sCD40L, and IL-10 at baseline (pre-C1) and after cycle 1 (post-C1), expressed in pg/mL; concentrations at SuFu were not determined. (D) TCRβ repertoire profiles from cycles 1 and 2 and at SuFu (day 204 after screening). For TCRβ analyses, each row represents an individual clonotype, and color intensity indicates relative clonotype abundance across time points. Clonotypes with bold labels were phenotyped by single-cell RNA sequencing (Figure S6). nd, not determined.

does not specifically enrich for tumor-reactive lymphocytes, and the trafficking and tissue distribution of the infused cell populations were not assessed; therefore, attenuation of tumor-directed activity by non-tumor-reactive cell populations cannot be excluded.

Although no objective responses were observed, SD occurred in 5 of 10 RECIST-evaluable patients (50.0%), corresponding to 29.4% of all 17 treated patients. Notably, three of five patients with SD had received prior immune checkpoint inhibitor therapy. The absence of objective responses cannot be attributed to a specific mechanism in these studies; in particular, the contribution of transient siRNA-mediated CBL-B silencing and persistence of modified cells could not be assessed because their kinetics were not directly quantified. Patients with SD nevertheless demonstrated systemic immune modulation, including $CD8^+$ T cell enrichment, induction of cytotoxic mediators, and expansion of dominant TCRβ clonotypes,[21] despite the absence of objective tumor regression. Separately, patient 104-02-005, who had discontinued APN401 following clinical progression and withdrawal in the patient's best interest, showed a reduction in target lesions on follow-up imaging approximately 170 days after the first infusion without intervening anticancer therapy. The significance of this delayed radiographic finding remains uncertain because iRECIST was not prospectively implemented.[22]

Exploratory treatment-associated immune changes were consistent with systemic immune modulation. Patients with SD showed increases in markers associated with cytotoxic T cell activation, including $CD137^+$ and TIM-3^+ $CD8^+$ T cells, plasma IFN-γ, and GrB, together with increased circulating B cell populations. GrB is a major effector protease of cytotoxic lymphocytes,[23] while CD137 can identify activated antigen-reactive T cells[24,25] and CD40L contributes to T cell-antigen-presenting cell interactions.[26] Increased circulating B cells may also be relevant given the

association of tumor-infiltrating B cells and tertiary lymphoid structures with favorable immunotherapy outcomes,[27] although the significance of circulating B cell changes remains uncertain. In patient 103-01-005, CXCL13 was robustly induced following anti-CD3/CD28 stimulation of longitudinal APN401 DPs; CXCL13 mediates CXCR5-dependent B cell recruitment and organization, including within tertiary lymphoid structures.[28] However, CXCL13 was assessed in DPs from this patient only, and a relationship with the treatment-associated increase in circulating B cells cannot be established.

The longitudinal increase in inhibitory receptors, including PD-1, TIM-3, and LAG-3, may reflect physiological feedback following immune activation, although an evolving dysfunctional or exhausted T cell state cannot be excluded without functional validation.[29–31] Baseline immune profiles also differed between outcome groups: patients with PD had higher circulating $CD8^+$ T cell frequencies and plasma GrB, whereas patients with SD had higher IFN-γ and selected MDSC subsets. These findings should not be interpreted as predictive biomarkers. Higher $CD8^+$ T cell and GrB levels in PD may reflect a pre-existing but functionally insufficient or dysregulated immune response,[29–31] whereas IFN-γ has pleiotropic roles in antitumor immunity and adaptive immune regulation.[32] The higher baseline MDSC levels in patients with SD appear counterintuitive given their generally immunosuppressive role; however, MDSCs are heterogeneous, and circulating abundance may not reflect their functional state within the tumor microenvironment.[33] The biological significance of these baseline differences therefore remains uncertain. Increased IL-10 observed during treatment in patients with PD may reflect counter-regulatory immune signaling; however, given the context-dependent functions of IL-10, its cellular source and biological significance cannot be inferred from circulating levels alone.[34]

Longitudinal observations in individual patients further illustrated heterogeneous immune response kinetics. In patient 103-01-005, dominant TCRβ clonotypes persisted during and beyond APN401 treatment,[21] including within $CD8^+$ effector- and central-memory populations, consistent with preclinical evidence that CBL-B deficiency can support sustained antitumor T cell responses and long-lasting antitumor memory.[12] In patient 104-02-005, early systemic immune changes and clonal T cell expansion preceded the later reduction in target lesion measurements. The antigen specificity of the expanded clonotypes was not established in either patient; therefore, although these observations are consistent with systemic immune modulation following transient CBL-B silencing, they do not establish a causal relationship between the observed immune changes and clinical outcome.

Several limitations of these studies are inherent to their phase 1 design, including the small sample size, heterogeneous tumor histologies, limited number of response-evaluable patients, and exploratory biomarker analyses not powered for statistical inference. The absence of objective responses limits conclusions regarding clinical activity, and the delayed radiographic observation in patient 104-02-005 remains difficult to interpret because iRECIST was not prospectively implemented. The duration of CBL-B silencing in APN401 cells after reinfusion and the persistence of the modified cells could not be directly determined because this would require specific tracking of infused cells to distinguish them from endogenous circulating PBMCs. Such labeling would require additional manipulation of the DP with the potential to affect cellular function and potency and was therefore not implemented. Furthermore, the studies did not include an *in vivo* control cohort receiving similarly processed autologous PBMCs without CBL-B-targeting siRNA; therefore, contributions of leukapheresis, electroporation, and PBMC reinfusion to the observed systemic immune changes cannot be excluded.

Future studies should determine whether the observed immune modulation translates into meaningful clinical benefit and whether baseline immune features can inform patient selection. The exploratory findings provide a rationale for evaluating repeated dosing and combination approaches with immune checkpoint inhibitors, while their potential to improve clinical activity remains to be established.[1–3] Pharmacologic targeting of CBL-B is also being explored, further supporting the translational relevance of this pathway.[35,36]

In summary, these phase 1 studies demonstrate the feasibility of decentralized, same day point-of-care manufacturing and repeated administration of autologous CBL-B-silenced PBMCs, together with a favorable safety profile. Exploratory immune changes observed during treatment provide biological rationale for further investigation of transient intracellular checkpoint modulation in larger, controlled studies.

MATERIALS AND METHODS

Study design and participants

The open-label multicenter phase 1 studies APN401-103 (European Union Clinical Trials Register: EU-CT 2020–002680-55) and APN401-104 (EU-CT 2023–504281-34/ClinicalTrials.gov: NCT06172894) evaluated the safety, feasibility, and RP2D of APN401 in patients with advanced treatment-refractory solid tumors. Both studies were approved by institutional ethics committees and conducted in accordance with the Declaration of Helsinki. All patients provided written informed consent. Eligible patients were $\geq$18 years of age with histologically confirmed advanced solid tumors, Eastern Cooperative Oncology Group (ECOG) performance status 0–2, measurable disease according to RECIST v.1.1[16] and $\geq$2 prior lines of systemic therapy. Dose escalation followed a conventional 3 + 3 design in APN401-103 and a BOIN design in APN401-104.

Treatment and dose escalation

Patients received escalating doses of APN401 ranging from dose level 1 (5×10^6 PBMCs/kg) to dose level 2 (1.5×10^7 PBMCs/kg) and dose level 3 (4.5×10^7 PBMCs/kg). Treatment was administered for up to six cycles at 3-week intervals. Each treatment cycle consisted of leukapheresis on day 1 followed by APN401 infusion on day 2. Subsequent treatment cycles were administered in the absence of disease progression, unacceptable toxicity, and active infection, based on

investigator assessment, provided that the manufactured product met release specifications and no criteria for early study termination were met. No lymphodepleting or immunosuppressive conditioning regimen was administered before APN401 infusion.

Endpoints and assessments

The primary endpoints were safety, tolerability, manufacturing feasibility, and determination of the RP2D. Secondary endpoints included preliminary clinical activity assessed by BOR according to RECIST v.1.1[16] and characterization of systemic immune modulation associated with APN401 treatment. Safety was monitored through clinical assessments and AE reporting, with DLTs evaluated during the 21-day observation period following the first infusion. Clinical activity was evaluated by BOR, duration of disease stabilization, and survival follow-up where available. BOR was defined as the best response observed from treatment initiation until disease progression, initiation of new anticancer therapy, or study discontinuation. Patients were considered evaluable for BOR if they had at least one post-baseline tumor assessment or discontinued because of disease progression. Exploratory pharmacodynamic analyses were performed on longitudinal plasma and PBMC samples collected before treatment cycles and included flow cytometry-based phenotyping, soluble mediator analysis, TCR repertoire sequencing, and single-cell transcriptomics. Detailed assay methodologies are provided in the supplemental methods.

Manufacturing and quality control

APN401 DPs were manufactured from autologous PBMCs collected by leukapheresis and transiently silenced for CBL-B using the EPiC manufacturing platform. PBMCs were electroporated with good manufacturing practice (GMP)-grade CBL-B-targeting siRNA incorporating 2′-O-methyl modifications designed to enhance DP potency; the resulting DP was formulated for infusion. A detailed description of the EPiC platform architecture is shown in Figure 1A and provided in the supplemental methods. APN401 was administered as a fresh, non-cryopreserved autologous PBMC product without an intervening cryopreservation and thawing step. APN401 batch release included pre-administration quality control testing (appearance, identity, viability, purity, quantity, and endotoxin). Additional testing for sterility, activity, and potency was performed post-administration using retained samples. Activity and potency testing using anti-CD3/CD28 stimulation assays is described in the supplemental methods. DP quality control results are shown in Figure S1.

Statistical analysis

As a phase 1 dose-escalation study, sample size was not based on formal statistical power calculations. Safety and clinical outcomes were summarized descriptively. Exploratory biomarker analyses were conducted to evaluate treatment-associated immune changes and differences between clinical outcome groups. These analyses were descriptive and hypothesis-generating and were not intended to establish statistical significance. Effect size-based approaches were used to characterize observed differences, with longitudinal analyses performed across predefined time points. Detailed statistical methodologies are provided in the supplemental methods.

DATA AND CODE AVAILABILITY

The data generated in this study are available from the corresponding authors upon reasonable request. Individual participant data are not publicly available due to patient privacy and confidentiality considerations.

ACKNOWLEDGMENTS

The authors acknowledge financial support from the Austrian Research Promotion Agency (FFG) under grant agreement no. FO999905116. The authors thank the patients and their families for participation in this study, as well as the clinical and technical staff at all participating sites for their contributions to study conduct and sample processing.

AUTHOR CONTRIBUTIONS

Conceptualization, A.D., N.W., T.B., T.F., S.B., and R. Gugenberger; methodology, A.D., N.W., T.B., A.B., R. Gugenberger, E.A., F.B., M.U., J.B., A.S., B.P., S. Seidl, H.M., S. Stranner, M.K., A.H., and S.B.; investigation, N.W., T.F., M.B., D.W., G.P., R. Greil, A.P.-S., B.S., S.B., E.A., F.B., M.U., J.B., A.S., B.P., S. Seidl, H.M., S. Stranner, M.K., A.H., and S.B.; data curation, A.D., E.A., F.B., M.U., J.B., A.S., B.P., S. Seidl, H.M., M.K., A.H., S.B., and R. Gugenberger; formal analysis, A.D., R. Gugenberger, A.B., A.H., S.B., and T.B.; resources, N.W., T.F., M.B., D.W., G.P., and R. Greil; funding acquisition, S.B.; project administration, A.D., S.B., and A.B.; supervision, A.D., N.W., T.F. and T.B.; visualization, A.D. and T.B.; writing – original draft, A.D.; writing – review and editing, A.D., N.W., T.B., T.F., M.B., D.W., G.P., R. Greil, S.B., S. Stranner, M.K., S.B., A.B., and R. Gugenberger All authors reviewed and approved the final manuscript and agree to be accountable for all aspects of the work.

DECLARATION OF INTERESTS

Authors affiliated with invIOs GmbH are or have been employees of the company and may hold equity or stock options. invIOs GmbH is developing APN401 and the EPiC platform described in this study. These authors report patents and/or patent applications related to the technology described herein. T.F. reports honoraria or advisory roles from MSD, Merck, BMS, BI, Roche, Sanofi, Amgen, Takeda, Johnson & Johnson, Beigene, Pierre Fabre, Pfizer, Pharma Mar, Astra Zeneca, Abbvie, Regeneron and invIOs GmbH. N.W. reports honoraria from BMS Celgene, Kite Gilead, Miltenyi Biotec, Terumo BCT, Therakos, Vertex, and research support from Sanofi Genzyme.

DECLARATION OF GENERATIVE AI AND AI-ASSISTED TECHNOLOGIES IN THE MANUSCRIPT PREPARATION PROCESS

During the preparation of this work, the authors used ChatGPT (OpenAI) to assist with drafting, language refinement, editing, and improving the clarity of the manuscript. The authors reviewed, edited, and validated all AI-generated content and take full responsibility for the final content of the publication. No artificial intelligence tool was used to generate, analyze, interpret, or verify study data, nor to make scientific conclusions.

SUPPLEMENTAL INFORMATION

Supplemental information can be found online at https://doi.org/10.1016/j.omton.2026.201357.

REFERENCES

1. Alsaafeen, B.H., Ali, B.R., and Elkord, E. (2025). Resistance mechanisms to immune checkpoint inhibitors: updated insights. Mol. Cancer *24*, 20. https://doi.org/10.1186/s12943-024-02212-7.

2. Huang, L., Zhu, H., and Shi, Y. (2025). Immune checkpoint inhibitors for the treatment of solid tumors and lymphoma in the past 26 years (2000–2025). J. Hematol. Oncol. *18*, 107. https://doi.org/10.1186/s13045-025-01734-x.

3. Zhang, D., Zhao, J., Zhang, Y., Jiang, H., and Liu, D. (2024). Revisiting immune checkpoint inhibitors: new strategies to enhance efficacy and reduce toxicity. Front. Immunol. *15*, 1490129. https://doi.org/10.3389/fimmu.2024.1490129.

4. Binnewies, M., Roberts, E.W., Kersten, K., Chan, V., Fearon, D.F., Merad, M., Coussens, L.M., Gabrilovich, D.I., Ostrand-Rosenberg, S., Hedrick, C.C., et al. (2018). Understanding the tumor immune microenvironment. Nat Med *24*, 541–550. https://doi.org/10.1038/s41591-018-0014-x.

5. Lei, X., Lei, Y., Li, J.K., Du, W.X., Li, R.G., Yang, J., Li, J., Li, F., and Tan, H.B. (2020). Immune cells within the tumor microenvironment. Cancer Lett. *470*, 126–133. https://doi.org/10.1016/j.canlet.2019.11.009.

6. June, C.H., O'Connor, R.S., Kawalekar, O.U., Ghassemi, S., and Milone, M.C. (2018). CAR T-cell immunotherapy for human cancer. Science *359*, 1361–1365. https://doi.org/10.1126/science.aar6711.

7. Neelapu, S.S., Locke, F.L., Bartlett, N.L., Lekakis, L.J., Miklos, D.B., Jacobson, C.A., Braunschweig, I., Oluwole, O.O., Siddiqi, T., Lin, Y., et al. (2017). Axicabtagene ciloleucel CAR T-cell therapy in refractory large B-cell lymphoma. N. Engl. J. Med. *377*, 2531–2544. https://doi.org/10.1056/NEJMoa1707447.

8. Fraietta, J.A., Lacey, S.F., Orlando, E.J., Pruteanu-Malinici, I., Gohil, M., Lundh, S., Boesteanu, A.C., Wang, Y., O'Connor, R.S., Hwang, W.T., et al. (2018). Determinants of response and resistance to CD19 CAR T-cell therapy. Nat Med *24*, 563–571. https://doi.org/10.1038/s41591-018-0010-1.

9. Tang, R., Langdon, W.Y., and Zhang, J. (2019). Regulation of immune responses by E3 ubiquitin ligase Cbl-b. Cell. Immunol. *340*, 103878. https://doi.org/10.1016/j.cellimm.2018.11.002.

10. Bachmaier, K., Krawczyk, C., Kozieradzki, I., Kong, Y.Y., Sasaki, T., Oliveira-dos-Santos, A., Mariathasan, S., Bouchard, D., Wakeham, A., Itie, A., et al. (2000). Negative regulation of lymphocyte activation and autoimmunity by CBL-B. Nature *403*, 211–216. https://doi.org/10.1038/35003228.

11. Chiang, J.Y., Jang, I.K., Hodes, R., and Gu, H. (2007). Ablation of CBL-B provides protection against transplanted and spontaneous tumors. J. Clin. Investig. *117*, 1029–1036. https://doi.org/10.1172/JCI29472.

12. Loeser, S., Loser, K., Bijker, M.S., Rangachari, M., van der Burg, S.H., Wada, T., Beissert, S., Melief, C.J.M., and Penninger, J.M. (2007). Spontaneous tumor rejection by CBL-B-deficient CD8+ T-cells. J. Exp. Med. *204*, 879–891. https://doi.org/10.1084/jem.20061699.

13. Lu, T., Chen, L., Mansour, A.G., Yu, M.J., Brooks, N., Teng, K.Y., Li, Z., Zhang, J., Barr, T., Yu, J., and Caligiuri, M.A. (2021). Cbl-b is upregulated and plays a negative role in activated human NK cells. J. Immunol. *206*, 677–685. https://doi.org/10.4049/jimmunol.2000177.

14. Triozzi, P., Kooshki, M., Alistar, A., Bitting, R., Neal, A., Lametschwandtner, G., and Loibner, H. (2015). Phase I clinical trial of adoptive cellular immunotherapy with APN401 in patients with solid tumors. J. Immunother. Cancer *3*, P175. https://doi.org/10.1186/2051-1426-3-S2-P175.

15. ClinicalTrials.gov (2025). A Phase 1, Open-Label, Dose-Ranging Study to Assess the Safety and Immunologic Activity of APN401. NCT02166255.

16. Eisenhauer, E.A., Therasse, P., Bogaerts, J., Schwartz, L.H., Sargent, D., Ford, R., Dancey, J., Arbuck, S., Gwyther, S., Mooney, M., et al. (2009). New response evaluation criteria in solid tumours: revised RECIST guideline (version 1.1). Eur. J. Cancer *45*, 228–247. https://doi.org/10.1016/j.ejca.2008.10.026.

17. Carnevale, J., Shifrut, E., Kale, N., Nyberg, W.A., Blaeschke, F., Chen, Y.Y., Li, Z., Bapat, S.P., Diolaiti, M.E., O'Leary, P., et al. (2022). RASA2 ablation in T-cells boosts antigen sensitivity and long-term function. Nature *609*, 174–182. https://doi.org/10.1038/s41586-022-05126-w.

18. Agliardi, G., Dias, J., Rampotas, A., Garcia, J., and Roddie, C. (2025). Accelerating and optimising CAR T-cell manufacture to deliver better patient products. Lancet Haematol. *12*, e57–e67. https://doi.org/10.1016/S2352-3026(24)00273-4.

19. Shah, M., Krull, A., Odonnell, L., de Lima, M.J., and Bezerra, E. (2023). Promises and challenges of a decentralized CAR T-cell manufacturing model. Front. Transplant. *2*, 1238535. https://doi.org/10.3389/frtra.2023.1238535.

20. Depil, S., Duchateau, P., Grupp, S.A., Mufti, G., and Poirot, L. (2020). Off-the-shelf allogeneic CAR T-cells: development and challenges. Nat. Rev. Drug Discov. *19*, 185–199. https://doi.org/10.1038/s41573-019-0051-2.

21. Robins, H.S., Campregher, P.V., Srivastava, S.K., Wacher, A., Turtle, C.J., Kahsai, O., Riddell, S.R., Warren, E.H., and Carlson, C.S. (2009). Comprehensive assessment of T-cell receptor beta-chain diversity in αβ T-cells. Blood *114*, 4099–4107. https://doi.org/10.1182/blood-2009-04-217604.

22. Seymour, L., Bogaerts, J., Perrone, A., Ford, R., Schwartz, L.H., Mandrekar, S., Lin, N.U., Litière, S., Dancey, J., Chen, A., et al. (2017). iRECIST: guidelines for response criteria for use in trials testing immunotherapeutics. Lancet Oncol. *18*, e143–e152. https://doi.org/10.1016/S1470-2045(17)30074-8.

23. Voskoboinik, I., Whisstock, J.C., and Trapani, J.A. (2015). Perforin and granzymes: function, dysfunction and human pathology. Nat. Rev. Immunol. *15*, 388–400. https://doi.org/10.1038/nri3839.

24. Wolfl, M., Kuball, J., Ho, W.Y., Nguyen, H., Manley, T.J., Bleakley, M., and Greenberg, P.D. (2007). Activation-induced expression of CD137 permits detection, isolation, and expansion of tumor-reactive T-cells. Blood *110*, 201–210. https://doi.org/10.1182/blood-2006-11-056168.

25. Ye, Q., Song, D.G., Poussin, M., Yamamoto, T., Best, A., Li, C., Coukos, G., and Powell, D.J., Jr. (2014). CD137 accurately identifies tumor-reactive T-cells. Clin. Cancer Res. *20*, 44–55. https://doi.org/10.1158/1078-0432.CCR-13-0945.

26. Beatty, G.L., Li, Y., and Long, K.B. (2024). Clinical and immunologic advances in CD40 agonist cancer therapy. Clin. Cancer Res. *30*, 2079–2088. https://doi.org/10.1158/1078-0432.CCR-23-3209.

27. Helmink, B.A., Reddy, S.M., Gao, J., Zhang, S., Basar, R., Thakur, R., Yizhak, K., Sade-Feldman, M., Blando, J., Han, G., et al. (2020). B cells and tertiary lymphoid structures promote immunotherapy response. Nature *577*, 549–555. https://doi.org/10.1038/s41586-019-1922-8.

28. Kazanietz, M.G., Durando, M., and Cooke, M. (2019). CXCL13 and its receptor CXCR5 in cancer: inflammation, immune response, and beyond. Front. Endocrinol. *10*, 471. https://doi.org/10.3389/fendo.2019.00471.

29. Joller, N., Anderson, A.C., and Kuchroo, V.K. (2024). LAG-3, TIM-3, and TIGIT: distinct functions in immune regulation. Immunity *57*, 206–222. https://doi.org/10.1016/j.immuni.2024.01.010.

30. Qin, S., Xu, L., Yi, M., Yu, S., Wu, K., and Luo, S. (2023). Targeting LAG-3, TIM-3, and TIGIT for cancer immunotherapy. J. Hematol. Oncol. *16*, 95. https://doi.org/10.1186/s13045-023-01499-1.

31. Andrews, L.P., Marciscano, A.E., Drake, C.G., and Vignali, D.A.A. (2017). LAG-3 as a cancer immunotherapy target. Nat. Rev. Cancer *17*, 201–212. https://doi.org/10.1038/nrc.2016.148.

32. Ivashkiv, L.B. (2024). IFNγ: signalling, epigenetics and roles in immunity and cancer immunotherapy. Nat. Rev. Immunol. *24*, 123–139. https://doi.org/10.1038/s41577-023-00926-5.

33. Kumar, V., Patel, S., Tcyganov, E., and Gabrilovich, D.I. (2016). The nature of myeloid-derived suppressor cells in the tumor microenvironment. Trends Immunol. *37*, 208–220. https://doi.org/10.1016/j.it.2016.01.004.

34. Oft, M. (2014). IL-10: master switch from tumor-promoting inflammation to anti-tumor immunity. Cancer Immunol. Res. *2*, 194–199. https://doi.org/10.1158/2326-6066.CIR-13-0214.

35. Augustin, R.C., Bao, R., and Luke, J.J. (2023). Targeting CBL-B in cancer immunotherapy. J. Immunother. Cancer *11*, e006007. https://doi.org/10.1136/jitc-2022-006007.

36. ClinicalTrials.gov (2021). A Study of NX-1607 in Patients with Advanced Malignancies. NCT05107674.

OMTON, Volume *34*

Supplemental information

Phase 1 evaluation of APN401, an autologous CBL-B-silenced PBMC therapy, in advanced solid tumors

Alexander Dohnal, Nina Worel, Thomas Bogenrieder, Thorsten Fuereder, Martin Burian, Dominik Wolf, Gerald Prager, Richard Greil, Anja Peterbauer-Scherb, Beate Schrenk, Stefan Bunka, Ernesto Abila, Felix Batrina, Maria Urban, Julia Buchberger, Alice Schwarzboeck, Bernhard Peball, Sandra Seidl, Hannes Muehleisen, Stefan Stranner, Mario Kuttke, Angela Halfmann, Sarah Bischof, Andreas Birbach, and Romana Gugenberger

Supplemental Figures

APN401 Batch-Manufacturing

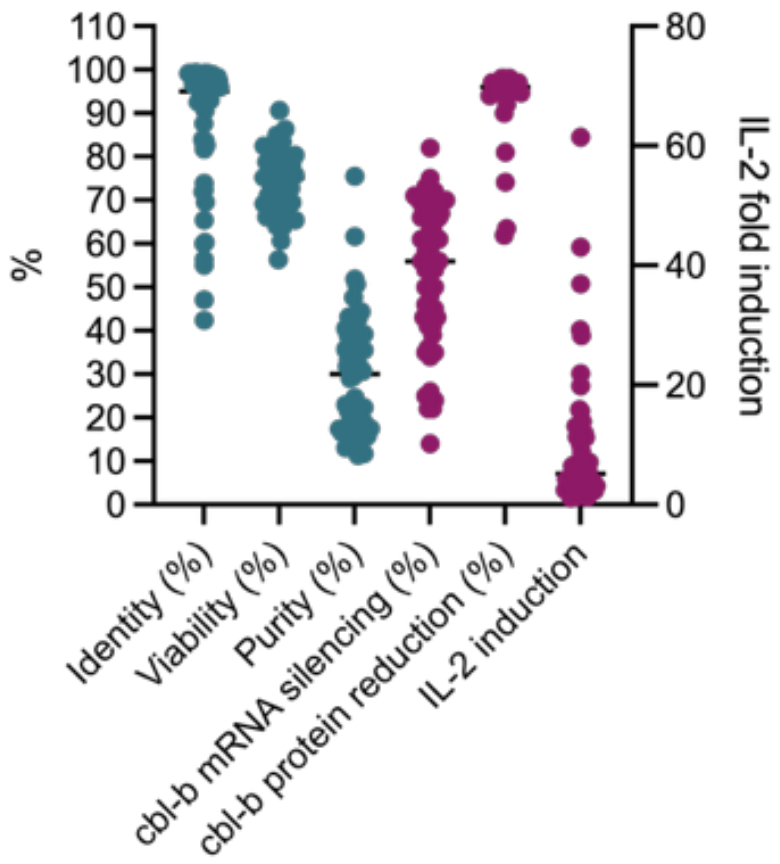

Figure S1. Release Testing and Functional Characterization of Manufactured APN401 Drug Product Batches. *Fifty-six APN401 DP batches underwent pre-infusion release testing for appearance, identity, viability, purity, quantity, and endotoxin. Post-infusion functional characterization following anti-CD3/CD28 stimulation assessed CBL-B silencing at the mRNA and protein levels relative to matched no-pulse controls (NPCs), as well as IL-2 secretion as a potency readout. Median values of tested batches are shown.*

Biomarker Assessment

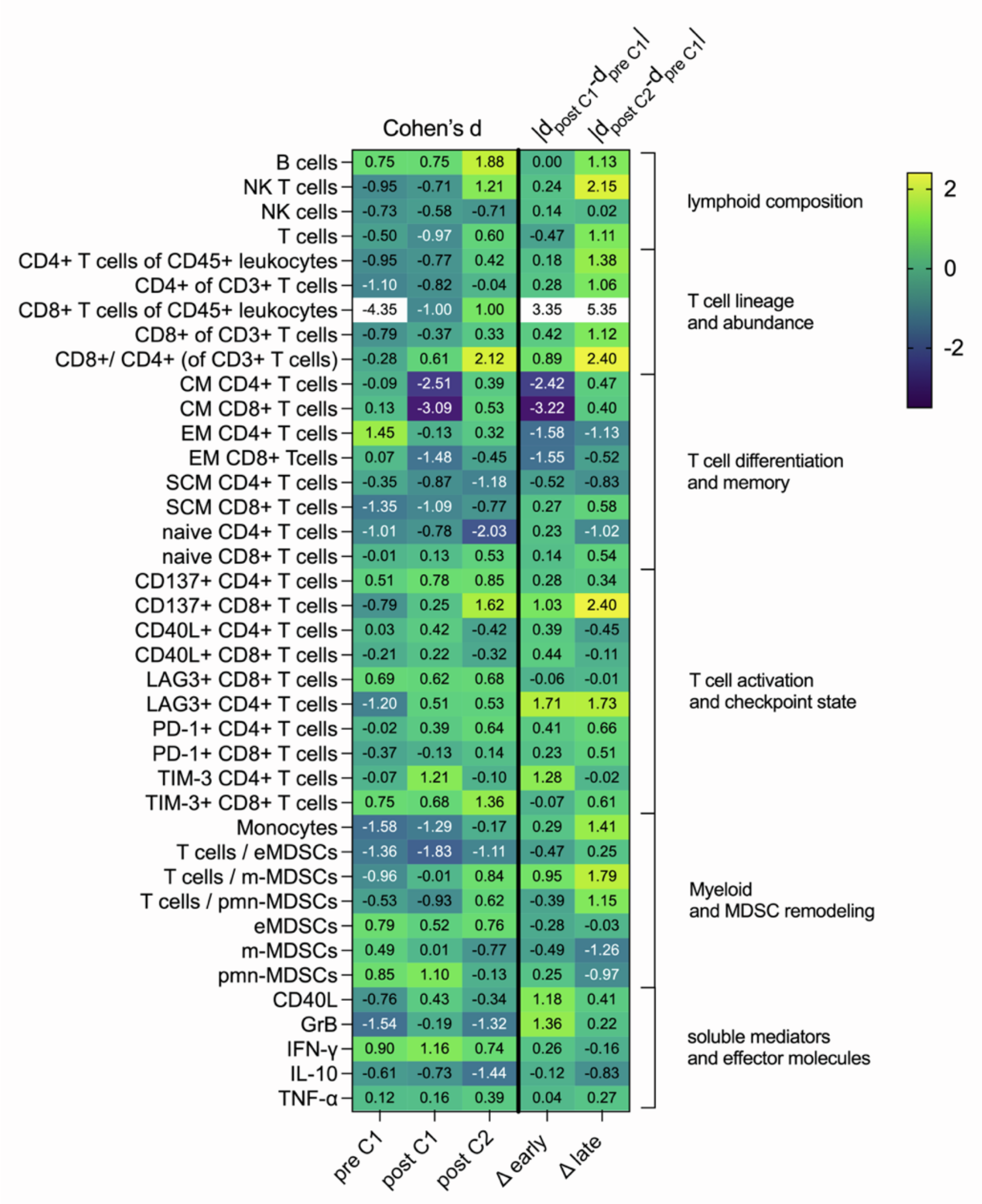

Figure S2. Systemic Immune Remodeling across APN401 Treatment Cycles. *Heatmap summarizing peripheral blood immune parameters at baseline (pre C1), post-cycle 1 (post C1), and post-cycle 2 (post C2), together with longitudinal changes from baseline. Standardized effect sizes (Cohen's d) compare patients with stable disease (SD) and progressive disease (PD). For longitudinal analyses, Cohen's d was calculated by comparing within-patient changes from baseline between the SD and PD groups. Positive effect sizes indicate higher values or greater increases in SD, whereas negative effect sizes indicate higher values or greater increases in PD. Immune parameters were assessed by multiparameter flow cytometry and plasma immunoassays. Absolute Cohen's d values of 0.2, 0.5, and 0.8 were interpreted descriptively as small, moderate, and large effects, respectively. Analyses included evaluable patients with paired samples at the indicated time points. Immune subsets included lymphoid populations and early-stage (eMDSC), monocytic (M-MDSC), and polymorphonuclear (PMN-MDSC) myeloid-derived suppressor cell subsets.*

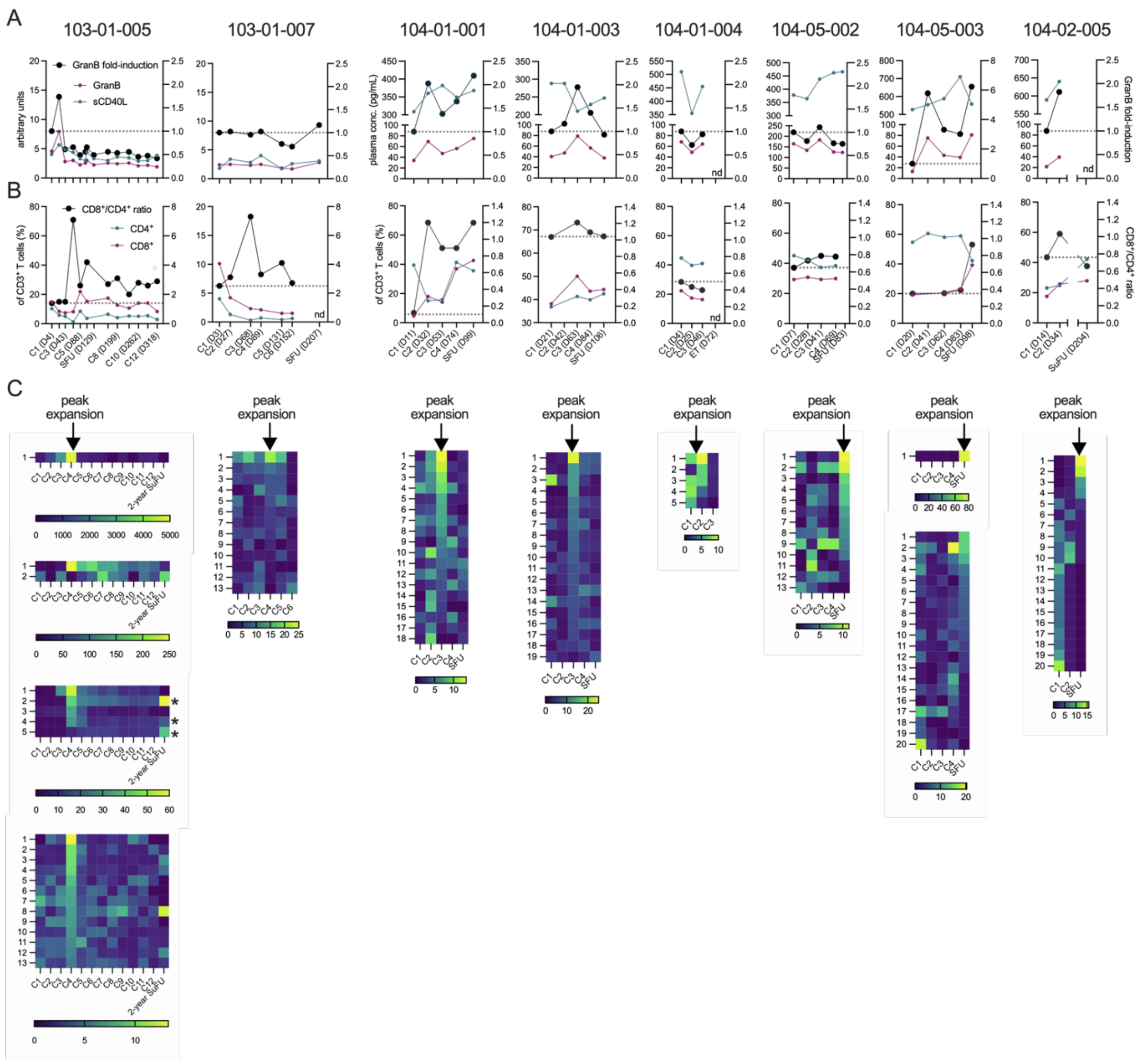

Figure S3. Longitudinal Immune Monitoring in Selected APN401-Treated Patients. *Exploratory immune monitoring parameters were evaluated longitudinally in patients with sufficient follow-up to assess immune kinetics. Peripheral blood samples were collected prior to each APN401 treatment cycle, at safety follow-up (SFU), and at survival follow-up (SuFu), where applicable. (A) Plasma concentrations of soluble immune mediators measured longitudinally using Olink® multiplex proteomics assays and/or electrochemiluminescence-based immunoassays. (B) Longitudinal frequencies of circulating $CD4^+$ and $CD8^+$ T cells and $CD8^+/CD4^+$ T-cell ratios determined by multiparameter flow cytometry. (C) Longitudinal dynamics of TCRβ clonotypes assessed by TCRβ repertoire sequencing during treatment and follow-up. Only clonotypes showing ≥5-fold enrichment during treatment are displayed and are shown relative to the lowest observed read count. Clonotypes also identified by single-cell RNA sequencing are indicated (*), where applicable.*

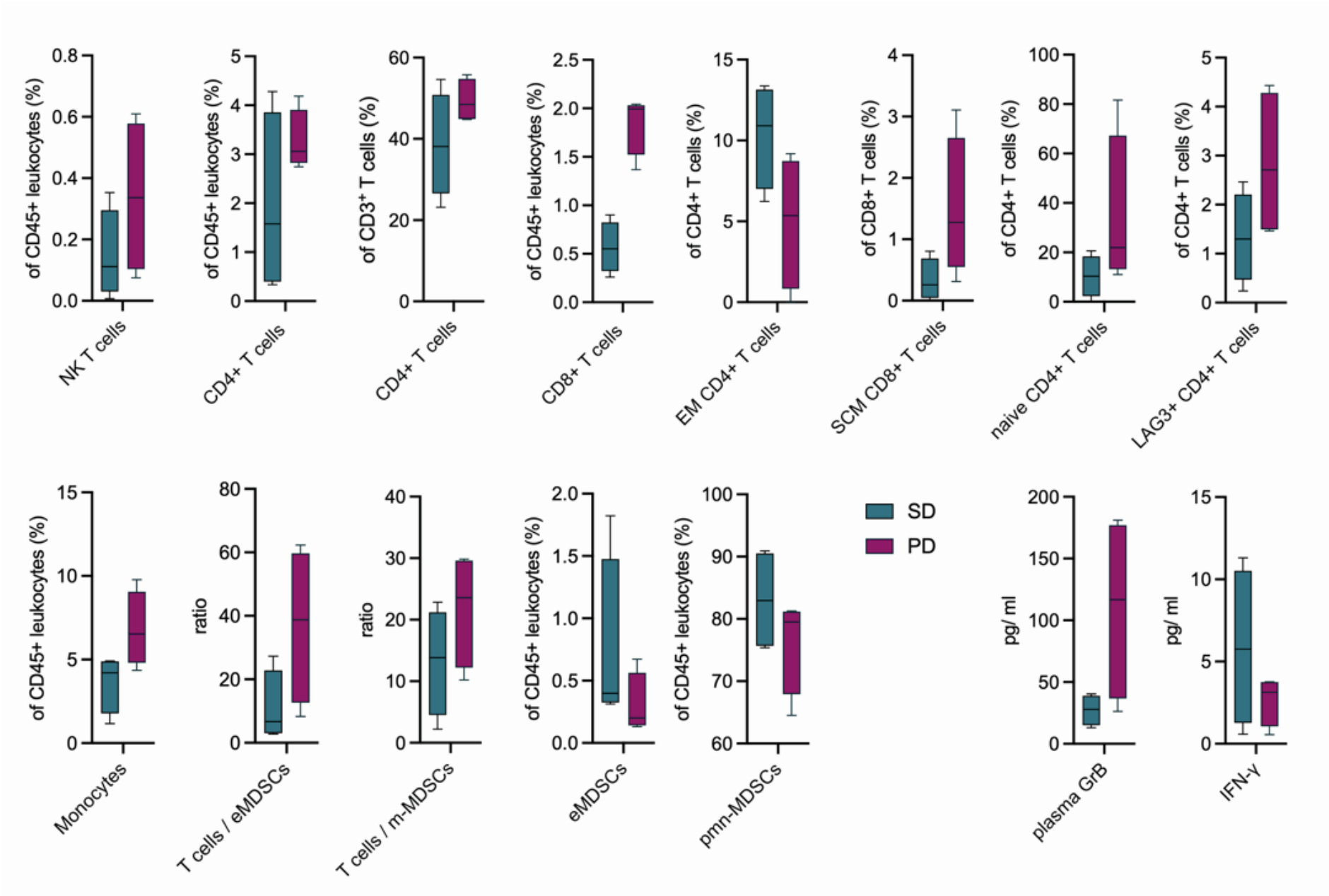

Figure S4. Baseline Peripheral Immune Features Associated with Stable Disease or Progressive Disease. *Baseline peripheral immune parameters were compared descriptively between patients with disease stabilization (SD, teal) and progressive disease (PD, purple). Box plots show immune cell subset frequencies measured by multiparameter flow cytometry, T-cell/MDSC ratios, and plasma concentrations of soluble mediators including GrB and IFN-γ. Boxes represent median and interquartile range, with whiskers indicating minimum and maximum values (SD, n = 4; PD, n = 4).*

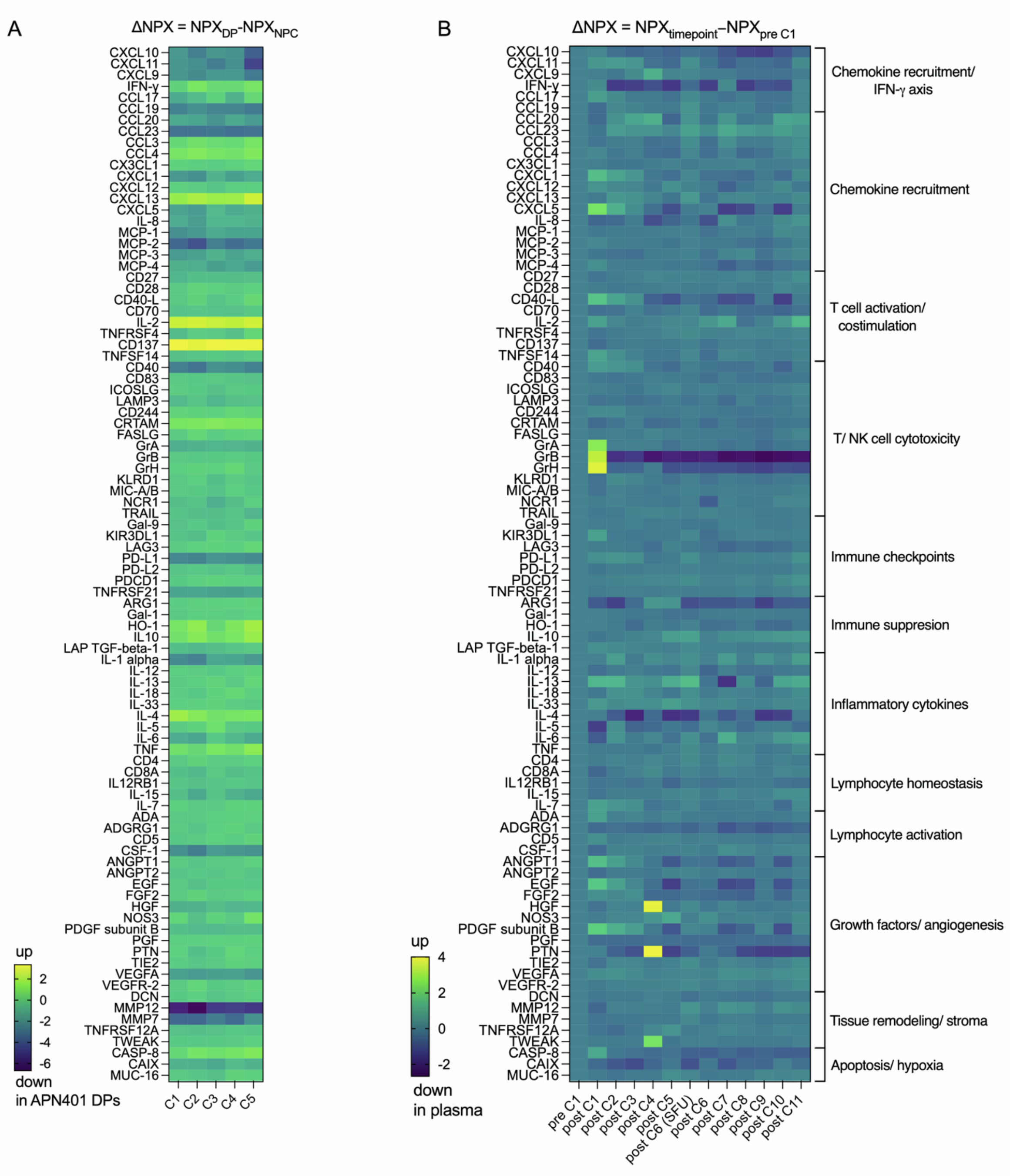

Figure S5. Longitudinal Proteomic Profiling of APN401 Drug Products and Plasma from Patient 103-01-005. *Heatmaps show longitudinal changes in immune-related proteins measured using the Olink® Immuno-Oncology panel. (A) Protein levels in culture supernatants from anti-CD3/CD28–stimulated APN401 DPs across manufacturing cycles (C1–C5) relative to matched no-pulse controls (NPCs). (B) Longitudinal plasma protein levels following APN401 administration (post C1 to post C11) relative to baseline (pre C1). The color scale indicates relative upregulation or downregulation of protein abundance. Normalized Protein eXpression (NPX) values represent log2-scaled normalized protein expression.*

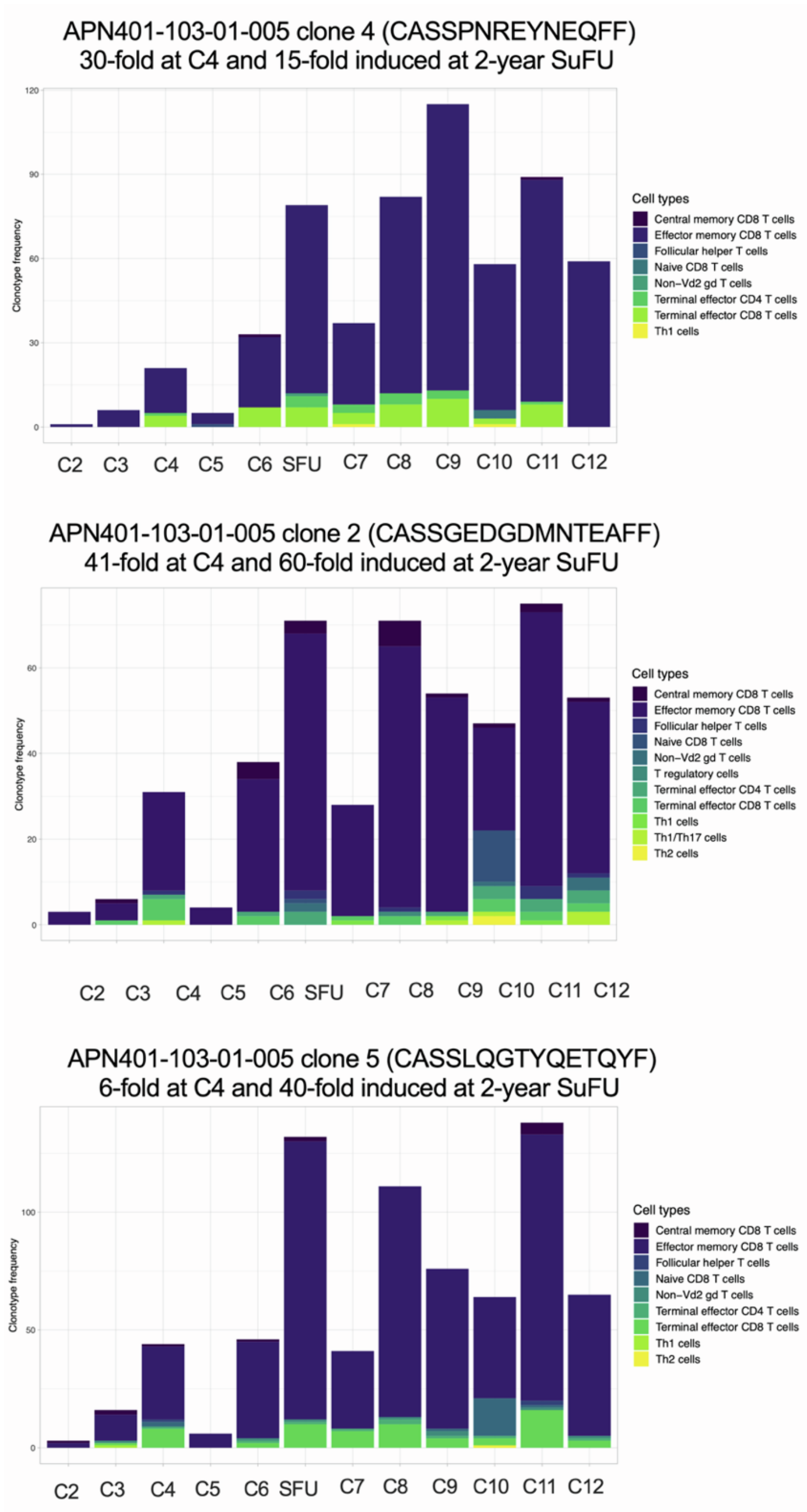

Figure S6. Phenotypic Characterization of Expanded TCRβ Clonotypes in Patient 103-01-005. *Phenotypic profiles of selected expanded TCRβ clonotypes in patient 103-01-005 are shown**.** Clonotypes detected by single-cell RNA sequencing of PBMCs were matched to corresponding clonotypes identified by TCRβ repertoire sequencing of APN401 DPs. Stacked bar plots show the phenotypic composition of matched clonotypes across treatment cycles and follow-up assessments, based on cell-type annotation derived from single-cell transcriptomic analysis. Fold induction in clonotype abundance relative to baseline is indicated for selected time points. All data are presented descriptively.*

Supplemental Tables

Table S1. Representativeness of Study Participants

Category	***Considerations***
Cancer type(s)/stage(s)/condition	*Patients with advanced, treatment-refractory solid tumors across multiple histologies. Not intended to represent any specific tumor epidemiology.*
Sex	*10 male, 7 female; not representative of tumor-specific distributions.*
Age	*Broad adult age range; representativeness limited by the small sample size and heterogeneous tumor types.*
Race/ethnicity	*All patients were White; racial and ethnic diversity was limited.*
Geography	*Conducted at four Austrian centers; limited global generalizability.*
Other considerations	*Selection based on eligibility for leukapheresis and early-phase trial participation.*
Overall representativeness	*Reflects a treatment-refractory Phase I population; limited generalizability due to sample size, tumor heterogeneity, and geographic restriction.*

Table S2. Patient Characteristics, Treatment and Clinical Response

Cohort	Subject ID	Tumor type	Prior lines	Dose level	Safety population	Treatment cycles	BOR-evaluable	RECIST BOR	Early termination/ reason
APN401 -103	103-01-001	HNSCC	≥2	1	No	0	No	na	/
APN401 -103	103-01-002	HNSCC	≥2	1	No	0	No	na	/
APN401 -103	103-01-003	HNSCC	≥3	1	Yes	3	Yes	PD	/
APN401 -103	103-01-004	Colorectal carcinoma	≥2	1	Yes	3	No	ET	OOS
APN401 -103	103-01-005	Appendiceal carcinoma	11	1	Yes	6+6*	Yes	SD	/
APN401 -103	103-01-006	Pancreatic carcinoma	≥2	2	Yes	2	No	ET	Best-interest withdrawal
APN401 -103	103-01-007	HNSCC	≥2	2	Yes	6	Yes	SD	/
APN401 -103	103-01-008	Pancreatic carcinoma	≥2	2	Yes	1	No	ET	Best-interest withdrawal
APN401 -104	104-01-001	SCLC	4§	2	Yes	4	Yes	SD	/
APN401 -104	104-01-002	NSCLC	8§	2	Yes	1	No	ET	OOS
APN401 -104	104-01-003	Colorectal carcinoma	6§	2	Yes	4	Yes	SD	/
APN401 -104	104-01-004	Cholangio carcinoma	5	2	Yes	3	Yes	PD	/
APN401 -104	104-02-001	HNSCC	3	3	Yes	2	No	ET	Patient withdrawal
APN401 -104	104-02-002	HNSCC	3	3	Yes	2	No	ET	Best-interest withdrawal
APN401 -104	104-02-005	HNSCC	3	3	Yes	2	No	ET&	Best-interest withdrawal
APN401 -104	104-04-001	Melanoma	4§	3	Yes	1	Yes	PD	/
APN401 -104	104-05-001	Uveal melanoma	4	3	Yes	2	Yes	PD	/
APN401 -104	104-05-002	Uveal melanoma	3§	3	Yes	4	Yes	PD	/
APN401 -104	104-05-003	Uveal melanoma	5§	3	Yes	3	Yes	SD	/

BOR, best overall response; ET, early termination; HNSCC, head and neck squamous cell carcinoma; NSCLC, non-small cell lung cancer; OOS, out of specification; PD, progressive disease; RECIST, Response Evaluation Criteria in Solid Tumors; SCLC, small cell lung cancer; SD, stable disease.

** Six APN401 cycles were administered during protocol-defined study treatment, followed by six additional cycles through a named-patient use program (12 cycles total).*

§ Received ≥1 prior line of immune checkpoint inhibitor therapy.

& Delayed reduction in target lesion measurements observed following early treatment discontinuation.

Table S3: Best Overall Response in the RECIST-Evaluable Population

BOR n (%)	DL1 (n = 2)	DL2 (n = 4)	DL3 (RP2D) (n = 4)	Overall (n = 10)
**SD**	1 (50.0%)	3 (75.0%)	1 (25.0%)	**5 (50.0%)**
**PD**	1 (50.0%)	1 (25.0%)	3 (75.0%)	**5 (50.0%)**
**ORR**	0 (0.0%)	0 (0.0%)	0 (0.0%)	0 (0.0%)
95% CI	(0.0%, 84.2%)	(0.0%, 60.2%)	(0.0%, 60.2%)	(0.0%, 30.8%)
**DCR**	1 (50.0%)	3 (75.0%)	1 (25.0%)	5 (50.0%)
95% CI	(1.3%, 98.7%)	(19.4%, 99.4%)	(0.6%, 80.6%)	(18.7%, 81.3%)

BOR, best overall response; CI, confidence interval; CR, complete response; DCR, disease control rate; DL, dose level; ORR, objective response rate; PD, progressive disease; PR, partial response; RECIST, Response Evaluation Criteria in Solid Tumors; RP2D, recommended Phase II dose; SD, stable disease. DCR was defined as CR + PR + SD; ORR was defined as CR + PR. The RECIST-evaluable population included 10 of 17 treated patients; seven patients were not evaluable for BOR because of early treatment termination.

Supplemental Material and Methods

APN401 Manufacturing, Drug Product Supply, and Release Testing

APN401 drug products (DPs) were manufactured at two GMP-compliant manufacturing sites supplying four clinical centers in Austria. To enable administration within the defined product shelf life, all clinical sites were located within approximately 5 hours' driving distance of the respective manufacturing facility. Leukapheresis products were collected at the clinical sites and, after meeting predefined starting-material specifications, were processed the following day using the EPiC® (Enhancement Platform for Immune Cells) manufacturing platform. Manufacturing was completed within approximately 6 hours, including pre-infusion release testing.

Manufacturing Process

Leukapheresis-derived peripheral blood mononuclear cells (PBMCs) were rebuffered into electroporation buffer (MaxCyte, Inc.) using spinning membrane filtration (LOVO®, Fresenius Kabi).

Good Manufacturing Practice (GMP)-grade Casitas B-lineage lymphoma proto-oncogene B (CBL-B)-targeting siRNA incorporating 2'-O-methyl modifications designed to enhance DP potency was introduced into PBMCs by electroporation using the MaxCyte GTx® system in accordance with the manufacturer's instructions.

Following electroporation, cells were rested for 30–60 minutes at room temperature and subsequently rebuffered for final formulation using spinning membrane filtration.

This modular configuration supports reproducible manufacturing across clinical sites and enables rapid production compatible with repeated dosing schedules.

APN401 siRNA characterization

The CBL-B-targeting siRNA used for APN401 is a chemically modified 21-mer RNA duplex incorporating 2'-O-methyl modifications. During product development, modified and unmodified siRNA variants were evaluated, with the modified siRNA demonstrating increased DP potency. The selected modified siRNA

was additionally evaluated for potential off-target effects. Transcriptome-level analyses did not identify evidence of sequence-specific off-target silencing under the tested conditions. Ribosome-occupancy analysis showed no significant differences indicative of broader effects on translational efficiency, and eCLIP-based analysis detected no binding of either the sense or antisense strand to off-target transcripts. Together, these analyses showed no detectable off-target binding or sequence-specific off-target silencing under the experimental conditions tested.

Pre-Infusion Release Testing

Pre-administration quality control testing was performed at the manufacturing site immediately before shipment and infusion. Release criteria included appearance, identity, quantity, viability, purity, and endotoxin.

- ***Appearance:*** *Visual inspection of the DP within the container closure system was performed against a standardized background and color reference, with aggregate presence and size recorded.*
- ***Identity and Quantity:*** *Determined by complete blood count (CBC) with differential. PBMCs were defined as the sum of lymphocytes and monocytes. Identity was defined as the percentage of PBMCs among total leukocytes (lymphocytes, monocytes, granulocytes), and quantity was calculated based on CBC results, target PBMC dose per kilogram, and patient body weight.*
- ***Viability:*** *Assessed by flow cytometry using antibodies against CD45, CD3, CD14, CD19, and CD56, with 7-aminoactinomycin D (7-AAD) as a viability dye. Antibodies included anti-CD3 (clone SK7, BD Biosciences, RRID: AB_2811220), anti-CD45 (clone 2D1, BD Biosciences, Cat# 655873, RRID: AB_2870390), anti-CD14 (clone M5E2, BD Biosciences, Cat# 345784, RRID: AB_2868810), anti-CD19 (clone 4G7, BD Biosciences, Cat# 345776, RRID: AB_2868804), and anti-CD56 (clone NCAM16.2, BD Biosciences, Cat# 345811, RRID: AB_2868832).*

- ***Purity:*** *Defined using the same flow cytometric panel as the proportion of viable CD45$^+$ PBMCs relative to all viable cellular events, including granulocytes and events reflecting red blood cells or platelets.*
- ***Endotoxin:*** *Measured in accordance with Ph. Eur. 2.6.14 and USP <85> using a chromogenic Limulus amebocyte lysate assay (Endosafe® nexgen-PTS™, Charles River Laboratories).*

Post-Infusion Functional Characterization

Functional characterization was performed post-administration using retained DP samples. Because CBL-B function is most relevant following T-cell receptor (TCR) activation, functional characterization was performed after anti-CD3/CD28 stimulation rather than by comparing unstimulated CBL-B expression immediately after manufacturing. DP and matched no-pulse control (NPC) samples collected prior to electroporation were stimulated with anti-CD3/CD28 monoclonal antibodies (UltraLEAF anti-human CD3 [clone OKT3, RRID: AB_11147370] and UltraLEAF anti-human CD28 [clone 28.2, RRID: AB_11150591], BioLegend) for 1 hour and cultured for a total of 41–45 hours at 37°C and 5% CO_2 under identical conditions.

Supernatants and cell lysates were shipped on dry ice to a central laboratory (VelaLabs GmbH, Vienna, Austria) for analysis.

- ***Activity:*** *Defined as downregulation of CBL-B protein and messenger RNA (mRNA) in stimulated DPs relative to matched NPC samples, assessed by electrochemiluminescence-based immunoassay (protein) and quantitative reverse transcription polymerase chain reaction (RT-PCR) (mRNA).*
- ***Potency:*** *Defined as upregulation of IL-2 secretion in DP culture supernatants relative to NPC controls, measured using a commercially available electrochemiluminescence immunoassay (V-PLEX Plus Proinflammatory Panel 1, Meso Scale Diagnostics).*
- ***Sterility:*** *Assessed in accordance with Ph. Eur. 2.6.1 and USP <71> using validated membrane filtration or direct inoculation methods.*

Sample Collection and Processing

Peripheral blood samples were collected prior to each treatment cycle and ***at safety follow-up (SFU) and survival follow-up (SuFu), where applicable,*** *for exploratory pharmacodynamic and immune monitoring analyses. PBMCs and plasma were isolated using BD Vacutainer® CPT™ tubes with sodium heparin according to the manufacturer's instructions. PBMCs were cryopreserved in CryoStor® CS10, and plasma samples were stored at −80°C until analysis. Whole blood collected in lithium heparin tubes was additionally processed for direct flow cytometric evaluation following red blood cell lysis.*

Soluble Immune Mediator Profiling

Plasma samples were analyzed for soluble immune mediators, including soluble CD40L (sCD40L), granzyme B (GrB), interferon-γ (IFN-γ), IL-2, IL-10, tumor necrosis factor-α (TNF-α), and additional cytokines. Analyses were performed at VelaLabs GmbH (Vienna, Austria) using Olink® multiplex proteomics assays and Meso Scale Diagnostics electrochemiluminescence-based immunoassays. Cytokine concentrations were summarized descriptively and evaluated longitudinally relative to baseline.

Flow Cytometry–Based Immune Phenotyping

Immune phenotyping was performed using multiparameter flow cytometry on a CytoFLEX LX flow cytometer (Beckman Coulter, Germany). Antibody panels (BioLegend, Germany) were used to characterize major lymphoid and myeloid subsets, as well as activation-associated markers and inhibitory receptor expression. Markers included CD45, CD3, CD4, CD8, CD45RA, CD45RO, CCR7, CD62L, CD27, CD14, CD19, CD56, CD11b, CD15, CD33, HLA-DR, CD40L, CD137, PD-1, TIM-3, LAG-3, and related targets. Myeloid-derived suppressor cell (MDSC) subsets were defined according to established phenotypic criteria (Bronte et al., 2016): monocytic MDSCs (M-MDSCs), $CD11b^{+}CD14^{+}HLA\text{-}DR^{low}/CD15^{-}$; polymorphonuclear MDSCs (PMN-MDSCs), $CD14^{-}CD11b^{+}CD15^{+}$; and early-stage MDSCs (eMDSCs), lineage-neagtive (Lin^{-})($CD3^{-}CD14^{-}CD15^{-}CD19^{-}CD56^{-}$)$HLA\text{-}DR^{-}CD33^{+}$. Flow cytometry data were

analyzed using standardized gating strategies to define immune cell subsets and quantify population frequencies based on predefined gating hierarchies.

T-Cell Receptor Sequencing and Clonotype Analysis

T-cell receptor β (TCRβ) repertoire profiling was performed by CeGaT GmbH (Tübingen, Germany) to assess treatment-associated clonal dynamics. Sequencing was conducted using the Illumina® TCR Beta-SR Panel. Productive TCRβ clonotypes were identified, and clonal dynamics were evaluated longitudinally by tracking changes in clonotype frequency over time. Clonotype diversity and persistence were assessed descriptively.

Single-Cell RNA Sequencing

Single-cell ribonucleic acid (RNA) sequencing was performed on selected cryopreserved PBMC samples by Lexogen GmbH (Vienna, Austria) to characterize systemic immune changes associated with APN401 treatment. Single-cell 5' gene-expression and T-cell V(D)J libraries were generated using the Chromium Next GEM Single Cell 5' platform (10x Genomics), with a target recovery of 10,000 cells per sample. Libraries were sequenced on Illumina platforms. Primary processing, including demultiplexing, gene-expression quantification, and V(D)J analysis, was performed using Cell Ranger. Downstream quality control, normalization, dimensionality reduction, clustering, and integration were performed using Seurat. Cell-type annotation was performed using SingleR with the Human Primary Cell Atlas and Monaco Immune Data reference datasets. TCR clonotype information derived from V(D)J sequencing was integrated with the single-cell transcriptomic data to characterize the phenotypic identity and longitudinal persistence of expanded T-cell clonotypes.

Statistical Interpretation of Biomarker Analyses

Immune and clinical parameters were analyzed descriptively, and exploratory comparisons were performed to characterize treatment-associated immune changes and differences between clinical outcome groups. Effect size–based approaches were used to quantify the magnitude of observed

differences and prioritize signals for descriptive follow-up in the small cohort setting. Cohen's d was used as a standardized descriptive measure of between-group differences, with positive values indicating higher levels in patients with stable disease (SD) and negative values indicating higher levels in patients with progressive disease (PD). Absolute Cohen's d values of 0.2, 0.5, and 0.8 were considered small, moderate, and large effects, respectively. Baseline analyses compared pretreatment immune parameters between patients with SD and PD. Longitudinal analyses compared within-patient changes from baseline (pre-cycle 1) to post-cycle 1 and post-cycle 2 between the SD and PD groups. Because these analyses were exploratory and hypothesis-generating rather than confirmatory, no adjustment for multiple comparisons was applied, and effect sizes were not interpreted as evidence of statistical significance. Statistical analyses were performed using GraphPad Prism version 11.0 (RRID: SCR_002798).

***Supplemental Reference**:*

Bronte V, Brandau S, Chen SH, et al. Recommendations for myeloid-derived suppressor cell nomenclature and characterization standards. Nat Commun. 2016;7:12150. doi:10.1038/ncomms12150.
